# RNase MRP subunit composition and role in 40S ribosome biogenesis

**DOI:** 10.1038/s41594-025-01690-7

**Published:** 2025-10-24

**Authors:** Eric M. Smith, Jimmy Ly, Sofia Haug, Iain M. Cheeseman

**Affiliations:** 1 Whitehead Institute for Biomedical Research, Cambridge, MA USA; 2 Department of Biology, Massachusetts Institute of Technology, Cambridge, MA USA

**Keywords:** RNA metabolism, Ribozymes

## Abstract

RNase MRP and RNase P are evolutionarily related complexes that facilitate rRNA and tRNA biogenesis, respectively. The two enzymes share nearly all protein subunits and have evolutionarily related catalytic RNAs. Notably, RNase P includes a unique subunit, RPP21, whereas no RNase MRP-specific proteins have been found in humans, limiting molecular analyses of RNase MRP function. Here, we identify the RNase MRP-specific proteins, C18orf21 (RMP24) and NEPRO (RMP64). C18orf21/RMP24 and RPP21 display significant structural homology, but we identify specific regions that drive interactions with their respective complexes. By targeting these RNase MRP-specific subunits, our functional analysis reveals that RNase MRP is essential for rRNA processing and preferentially required for 40S ribosome biogenesis. Finally, we determine that disease-associated mutations in RMP64 impair its association with RNase MRP subunits. Together, our findings elucidate the molecular determinants of RNase MRP function and underscore its critical role in ribosome biogenesis and disease.

## Abstract (web summary)

The authors identify the human RNase MRP-specific proteins, RMP24 (C18orf21) and RMP64 (Nepro), define their role in 40S ribosome biogenesis, and reveal how disease-linked RMP64 mutations disrupt complex assembly.

## Main

**Fig. 1:**
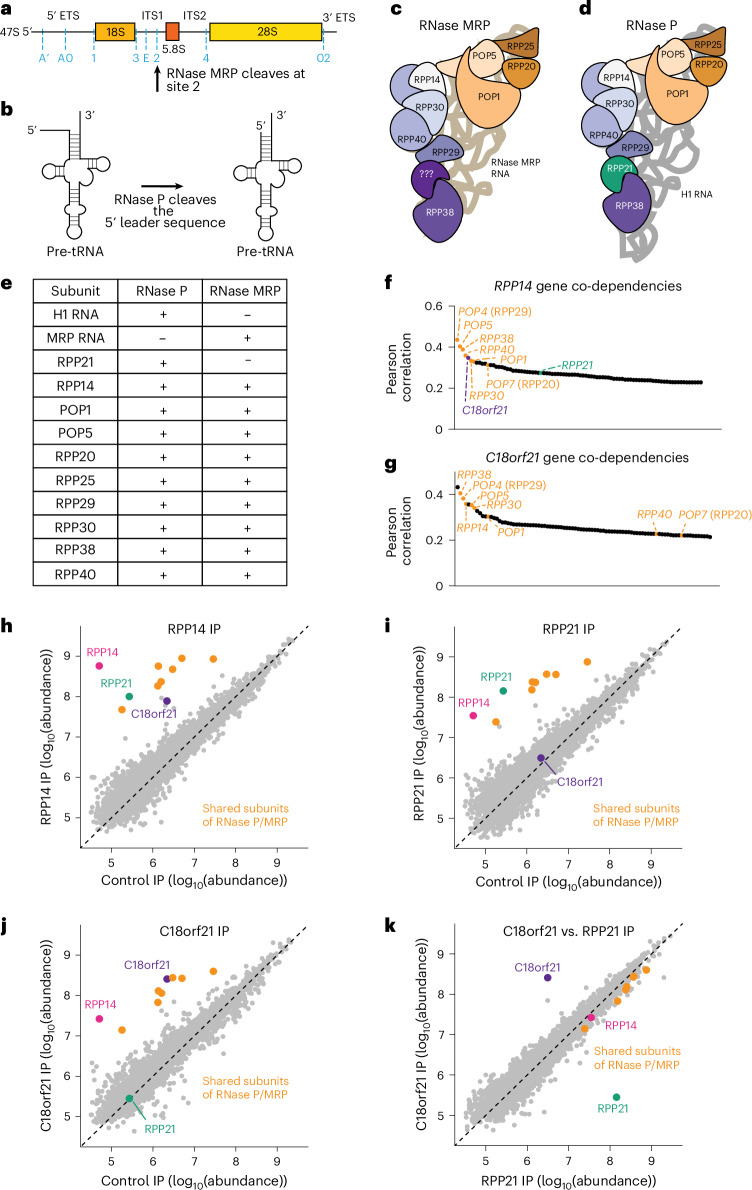
C18orf21 is associated with the shared subunits of the RNase P and RNase MRP complexes. **a**, A schematic of the 47S rRNA transcript. Endonucleolytic cleavage sites in the ETS and ITS are marked in blue. RNase MRP cleaves at site 2 in ITS1. **b**, A schematic of a precursor tRNA before and after the cleavage of the 5′ leader sequence by RNase P. **c**, A cartoon representation of RNase MRP. **d**, A cartoon representation of RNase P. **e**, Subunits that are found in the RNase P complex, the RNase MRP complex or both complexes. **f**, The genes with the highest Pearson correlation coefficients to RPP14. These coefficients are acquired from the CRISPR–Cas9 targeting effect scores in the DepMap database (DepMap Public 24Q2). The shared RNase P and MRP components are shown in orange, C18orf21 is shown in purple and RPP21 is shown in green. **g**, The genes with the highest Pearson correlation of CRISPR–Cas9-based targeting effect scores to C18orf21 in the DepMap database (DepMap Public 23Q4). The shared RNase P and MRP components are shown in orange. **h**, Scatter plot showing the abundance of the proteins detected in RPP14–GFP compared to control GFP IP–MS experiments. The shared components of the RNase P/MRP complexes are highlighted in orange, except RPP14 which is shown in pink. C18orf21 is highlighted in purple and RPP21 is highlighted in green. **i**, The same as in **h** except comparing RPP21–GFP to control GFP immunoprecipitation. **j**, The same as in **h** except comparing C18orf21–GFP to control GFP immunoprecipitations. **k**, The same as in **h** except comparing C18orf21–GFP to RPP21–GFP immunoprecipitations.

Ribosomal RNA (rRNA) is the most abundant cellular RNA molecule^1,2^. The production and maturation of rRNA are critical for ribosome function^3^. In humans, there are four mature rRNAs that are produced from two different non-coding RNA transcripts^3^. The 5S rRNA is transcribed by RNA polymerase III, whereas the 5.8S, 28S and 18S rRNAs are transcribed as a polycistronic transcript by RNA polymerase I (Fig. 1a)^4^. The mature 5.8S, 28S and 18S rRNAs are formed through endonucleolytic cleavage at specific sites in externally (ETS) and internally transcribed spacers (ITS), followed by exonucleolytic action^5–7^. The ribonucleoprotein complex RNase MRP acts as a site-specific endonuclease to cleave pre-rRNA at site 2 in the ITS1 (Fig. 1a)^8,9^. The human RNase MRP complex is composed of a catalytic RNA subunit, termed RNase MRP RNA, and at least nine protein subunits (POP1, POP5, RPP38, RPP30, POP4 (RPP29), RPP25, POP7 (RPP20), RPP14, and RPP40)^10–18^. The human RNase MRP RNA is transcribed from the *RMRP* gene, which is conserved across eukaryotes^13,19,20^. The importance of RNase MRP is highlighted by the fact that substitutions in the RNA component lead to inviable yeast, and knockout of *RMRP* is embryonic lethal in mice^21,22^. Additionally, in humans, mutations in the either the protein subunits or RNA subunit of RNase MRP, or the promoter of *RMRP*, can cause several disorders, including cartilage hair hypoplasia, metaphyseal dysplasia, anauxetic dysplasia, kyphomelic dysplasia and Omenn syndrome^23–29^. These disorders are characterized by growth impairment, skeletal dysplasia and immune deficiencies^23,26^.

RNase MRP is a fascinating example of evolution, in which a core cellular machine was repurposed to carry out a new function. RNase MRP is evolutionarily related to the ribonucleoprotein complex RNase P^20,30–32^. Like RNase MRP, RNase P acts as an endonuclease, but cleaves the 5′ leader sequence of pre-tRNAs (Fig. 1b)^33^. Owing to its essential function in tRNA biogenesis, RNase P is conserved across all three domains of life^34^. RNase MRP is thought to have evolved in eukaryotes as a result of the duplication of the RNase P RNA gene, which gave rise to the gene that encodes the RNase MRP RNA^10,30^. Structural and biochemical studies of *S. cerevisiae* RNase P and RNase MRP (RNase P/MRP) have revealed that the two RNA subunits share catalytic domains and the mechanism of RNA-substrate cleavage, despite differences in RNA sequence^11,12,30,35^. In addition to their similar RNA subunits, RNase P/MRP share common protein subunits (Fig. 1c–e)^10^. In humans, only one protein, RPP21, has been identified as an RNase P-specific protein^36^. Although there are two RNase MRP-specific subunits in *S. cerevisiae*, there are no known homologs to these proteins in metazoans, and no other RNase MRP-specific proteins have been identified^13,37,38^.

Here, we identify the uncharacterized protein C18orf21/RMP24 as an RNase MRP-specific protein subunit. Using structural predictions, we find that C18orf21/RMP24 has an amino-terminal domain that shares many structural features with RPP21. Despite the predicted structural similarities between the two proteins, we found specific regions that confer specificity of C18orf21/RMP24 and RPP21 for RNase MRP and RNase P, and reveal a critical role for C18orf21/RMP24 as a member of the RNase MRP complex in rRNA processing, ribosome biogenesis and cellular fitness. Finally, we leverage the identification of C18orf21/RMP24 to define NEPRO/RMP64 as an additional subunit of the RNase MRP complex. We show that a disease-associated RMP64 substitution causes the protein to have diminished interactions with components of the RNase MRP complex. Together, the results of this work reveal two unique subunits of the RNase MRP complex.

## Results

### C18orf21 associates with the shared subunits of the RNase P/MRP complexes

**Extended Data Fig. 1:**
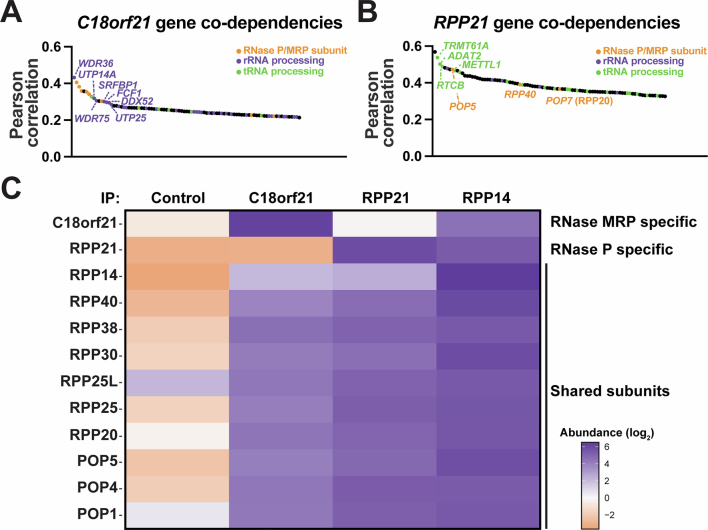
C18orf21 is associated with the shared subunits of the RNase P and RNase MRP complexes. (**a**) A waterfall plot showing the genes with the highest Pearson correlation of CRISPR-Cas9 based targeting effect scores to C18orf21 in the Depmap database (Depmap Public 23Q4). The shared RNase P and MRP components are shown in orange. Genes involved in rRNA processing are shown in purple and genes involved in tRNA processing are shown in green. (**b**) A waterfall plot showing the genes with the highest Pearson correlation of CRISPR-Cas9 based targeting effect scores to RPP21 in the Depmap database (Depmap Public 23Q4). The shared RNase P and MRP components are shown in orange. Genes involved in rRNA processing are shown in purple and genes involved in tRNA processing are shown in green. (**c**) A heat map showing the scaled relative precursor ion abundances of each RNase P/MRP component immunoprecipitated from cell lines ectopically expressing the indicated GFP tagged construct.

To identify protein subunits that distinguish RNase MRP complex from the RNase P complex, we evaluated genetic co-dependencies across cancer cell lines. The use of functional genetics in human cells has enabled effective strategies to identify cofunctional genes, including factors that function as part of the same protein complex^39,40^. To identify previously uncharacterized proteins, we conducted an analysis of gene coessentiality using genome-wide CRISPR–Cas9-based viability screens, performed on >1,000 cells lines as part of the Project Achilles Cancer Dependency map^41^. On the basis of similar gene essentiality requirement patterns across these cell lines, we found that a shared component of the RNase P/MRP complexes, *RPP14*, was genetically correlated with their other shared components. Surprisingly, we also found that *RPP14* had a strong co-dependency with the uncharacterized gene *C18orf21* (Fig. 1f). Reciprocally, we found that *C18orf21* displayed similar genetic-dependency requirements to eight out of nine shared genes between the RNase P/MRP complexes (Fig. 1g). *C18orf21* also displays strongly correlated functional requirements with several genes involved in rRNA production (Extended Data Fig. 1a). In contrast to the strong correlation of *C18orf21* with the components of the RNase P/MRP complexes, the RNase-P-specific component *RPP21* displayed genetic correlations with only three of the shared RNase P/MRP components, as well as correlations with several tRNA-processing factors (Extended Data Fig. 1b). Similarly, recent genome-wide perturbation studies, combined with single-cell sequencing (Perturb-Seq), identified similarities between *C18orf21* and genes associated with rRNA biogenesis on the basis of transcriptome similarities in depleted cells, whereas *RPP21* displayed phenotypic similarities with tRNA-processing genes^42^. Additionally, machine-learning-based protein complex predictions suggest that C18orf21 interacts with the shared components of RNase P/MRP^43,44^.

On the basis of the cofunctional relationships that we observed between *C18orf21* and members of the RNase P/MRP complexes, we next sought to determine whether C18orf21 interacts with any of the shared components in cells. To identify binding partners for C18orf21, we conducted immunoprecipitation–mass spectrometry (IP–MS) experiments in HeLa cells. In particular, we isolated carboxy-terminally-tagged GFP fusion proteins for C18orf21, RPP14, a shared subunit of both RNase P/MRP and the RNase-P-specific protein RPP21. RPP14 immunoprecipitations isolated each of the nine protein subunits that are shared by RNase P and RNase MRP, as well as isolating both RPP21 and C18orf21 (Fig. 1h and Supplementary Table 1). By contrast, we found that RPP21 purifications isolated all nine of the expected binding partners from the RNase P complex, but did not co-immunoprecipitate with C18orf21 (Fig. 1i). Finally, we found that C18orf21 isolated the nine subunits common to both RNase P and RNase MRP, but did not isolate RPP21 (Fig. 1j; see also ref. ^45^). Thus, C18orf21 and RPP21 display mutually exclusive protein interactions, but each protein associates with the shared subunits of the RNase P and RNase MRP complexes (Fig. 1k and Extended Data Fig. 1c).

### C18orf21/RMP24 acts as an RNase-MRP-complex-specific protein in cells

**Fig. 2:**
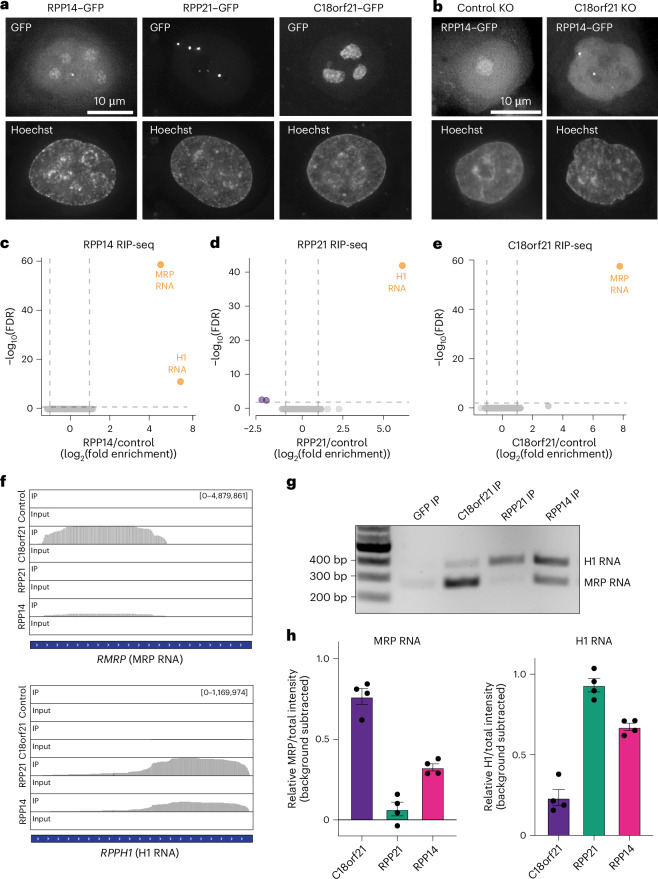
C18orf21 (RMP24) is a subunit of the RNase MRP complex. **a**, Representative Z-projected images from three biological replicates, taken by live-cell imaging, of cells ectopically expressing C-terminally GFP-tagged versions of C18orf21, RPP21 or RPP14. Images were deconvolved, and each set of images is scaled differently to highlight localization of each component. **b**, Representative Z-projected images from three biological replicates taken by live-imaging cells that ectopically express C-terminally GFP-tagged RPP14 and are targeted with either a control guide (*HS1* locus) or a guide targeting *C18orf21*. Images were deconvolved, and each image is scaled differently to highlight the differences in localization that were observed. **c**, Volcano plot showing the log_10_(FDR) on the *y* axis and fold enrichment on the *x* axis obtained from RPP14–GFP compared to control GFP RIP–RNA-seq experiments performed in duplicate. **d**, The same as in **c** except for RPP21–GFP compared to control GFP RIP-seq experiments. **e**, The same as in **c** except for C18orf21–GFP compared to control GFP RIP-seq experiments. **f**, RNA-seq read-coverage plots displaying the RMRP or RPPH1 reads that were enriched in the GFP control, C18orf21, RPP14 and RPP21 immunoprecipitations. **g**, Representative agarose gel stained with ethidium bromide showing the results of RIP–RT–PCR experiments. **h**, Quantitation of the integrated pixel intensity of either the intensity of the RNase MRP RNA band (left) or H1 band (right) with the GFP RIP–RT–PCR background signal subtracted. The *y* axis shows the ratio of the background subtracted integrated intensity of either MRP RNA or H1 RNA to the sum of intensities for both RNA species. The error bars represent the s.e.m. from four replicates.

**Extended Data Fig. 2:**
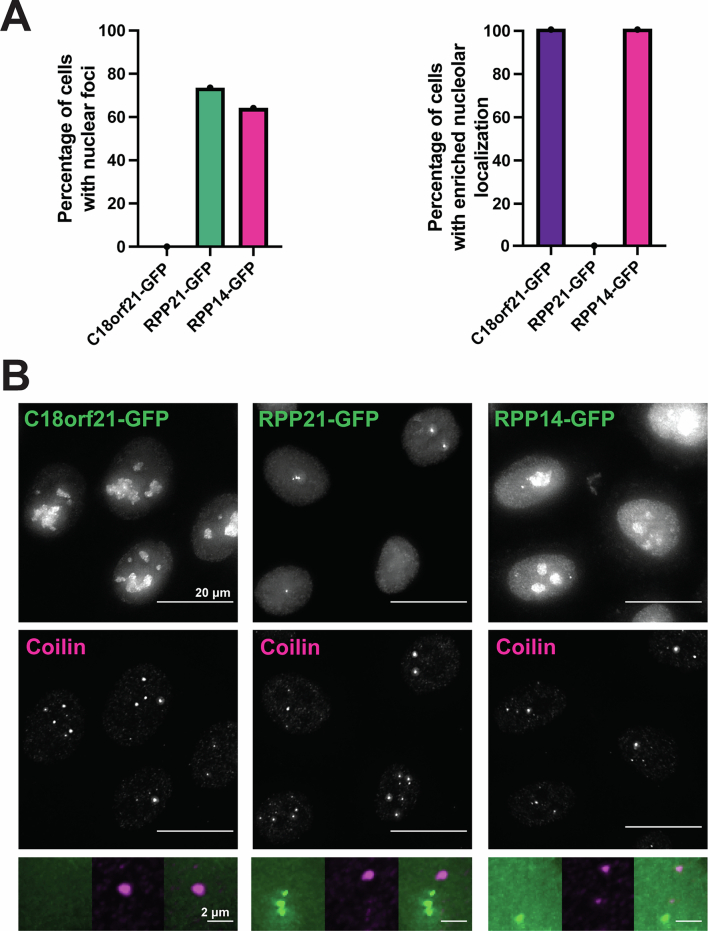
RPP21 and RPP14 localize to nuclear foci that are distinct from Cajal bodies. (**a**) Two bar graphs detailing the localization patterns of C18orf21-GFP, RPP21-GFP, and RPP14-GFP in HeLa cells. The graph on the left shows the percentage of cells that contained nuclear puncta. Nuclear puncta were defined as GFP signal greater than 0.25 µm in size and with an integrated pixel intensity above background. The graph on the right shows the percentage of cells that contain an enriched nucleolar GFP signal. The number of cells counted for each condition were: C18orf21 n=86, RPP14 n=39, and RPP21 n=34 cells. (**b**) Representative co-immunofluorescence images from two biological replicates of cell lines that ectopically express the indicated C-terminally GFP-tagged construct. Prior work suggested that RNase P localizes to Cajal bodies^36,80^. To mark Cajal bodies we co-stained each cell line for Coilin using an anti-coilin antibody (see materials and methods). The bottom inset depicts a magnified view of the merged GFP and Coilin staining. Here we find that C-terminally tagged constructs are not found in Cajal bodies suggesting that RNase P does not localize to these structures.

Our genetic-interaction and IP–MS analyses suggest that C18orf21 and RPP21 display mutually exclusive interactions with RNase P/MRP. We hypothesized that, as RPP21 is specific to RNase P, C18orf21 could be specific to RNase MRP such that both of these proteins interact with shared components, but not each other. To evaluate this model, we next assessed the cellular properties of C18orf21 and RPP21. Earlier work suggested that RNase MRP and RNase P localize to distinct compartments in the nucleus^36,46^. Therefore, we analyzed the cellular localization of C18orf21, the shared RNase P/MRP subunit RPP14 and the RNase-P-specific protein RPP21 as GFP fusions in HeLa cells. We found that RPP14 displayed localization to the nucleus, nuclear puncta and nucleolus, consistent with the combined reported localization for both RNase P and RNase MRP (Fig. 2a and Extended Data Fig. 2a,b). By contrast, RPP21–GFP localized to nuclear foci, but not the nucleolus, consistent with its localization as an RNase-P-specific protein (Fig. 2a and Extended Data Fig. 2). We found that C18orf21 localized only to the nucleolus, but not throughout the nucleoplasm, consistent with a role in promoting the specific activities of RNase MRP (Fig. 2a and Extended Data Fig. 2a). To evaluate whether C18orf21 is required for the nucleolar localization of other RNase MRP components, we monitored RPP14 localization in C18orf21-knockout cells. Eliminating C18orf21 led to a loss of the nucleolar population of RPP14, but did not disrupt RPP14 localization to nuclear foci (Fig. 2b).

Although RNase P and RNase MRP share most protein subunits, they have distinct catalytic RNA components (H1 RNA versus RNase MRP RNA). Because C18orf21 and RPP21 display differential localization and form mutually exclusive complexes with the shared subunits of RNase P/MRP, we sought to determine which RNA C18orf21 associates with in cells. To test whether C18orf21 interacts with H1 RNA or RNase MRP RNA in cells, we performed GFP immunoprecipitations followed by RNA sequencing and reverse transcription PCR (RT–PCR) with ribonucleoprotein immunoprecipitation (RIP) analysis. We found that immunoprecipitation of RPP14–GFP isolated both H1 RNA and RNase MRP RNA, consistent with it being a member of both the RNase P and RNase MRP complexes (Fig. 2c,f–h; Extended Data Fig. 2b and Supplementary Table 2). C18orf21–GFP isolated the RNase MRP RNA, but not the H1 RNA, whereas RPP21–GFP pulled down H1 RNA (Fig. 2d–h). On the basis of the protein and RNA interactions, genetic associations and subcellular localizations, we propose that C18orf21 is a specific subunit of RNase MRP and will refer to C18orf21 as ribonuclease MRP subunit p24 (RMP24).

### RMP24 is structurally related to RPP21

**Fig. 3:**
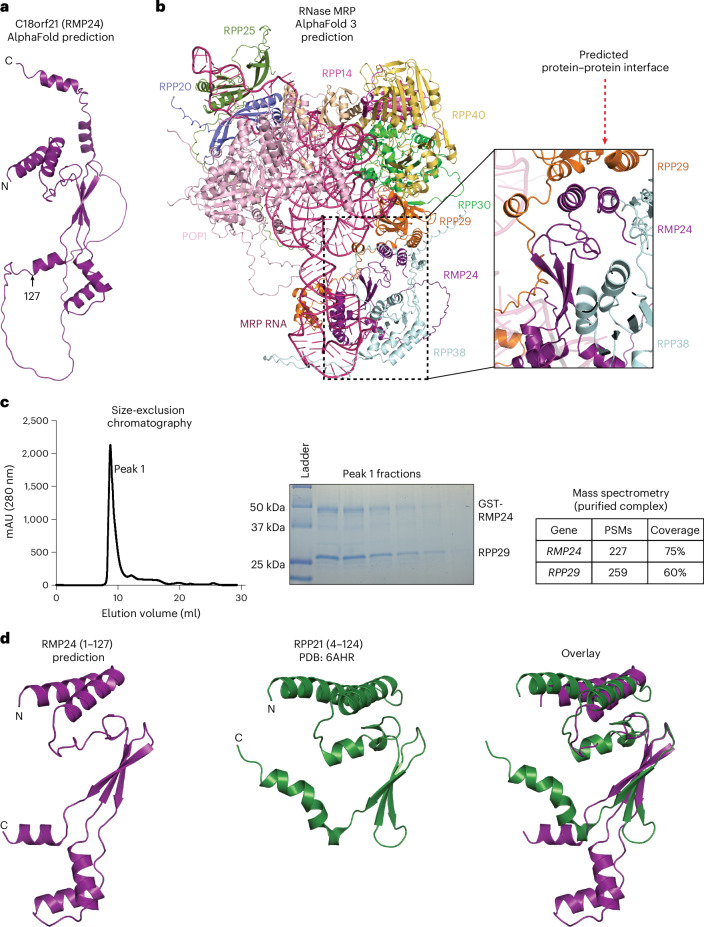
RMP24 is predicted to have structural homology with RPP21. **a**, Cartoon representation of the structure of RMP24, predicted by AlphaFold. **b**, A cartoon representation of the predicted structure of human RNase MRP, created using AlphaFold 3 (ref. ^47^). The magnified view to the right of the full structure highlights the predicted contacts between RMP24 and RPP29. **c**, A representative chromatogram from two biological replicates, resulting from passing the RMP24–RPP29 complex over a Superdex S200-increase column. A Coomassie-stained SDS–PAGE gel shows the peak fractions eluted from the Superdex S200-increase column. A table showing the peptide spectrum matches (PSMs) and coverage of RMP24 and RPP29 obtained from injecting the purified complex on the LC–MS. **d**, An illustration of the predicted structure of RMP24 (residues 1–127) and RPP21 (residues 4–124) from PDB ID 6AHR and an overlay of the two structures^61^.

**Extended Data Fig. 3:**
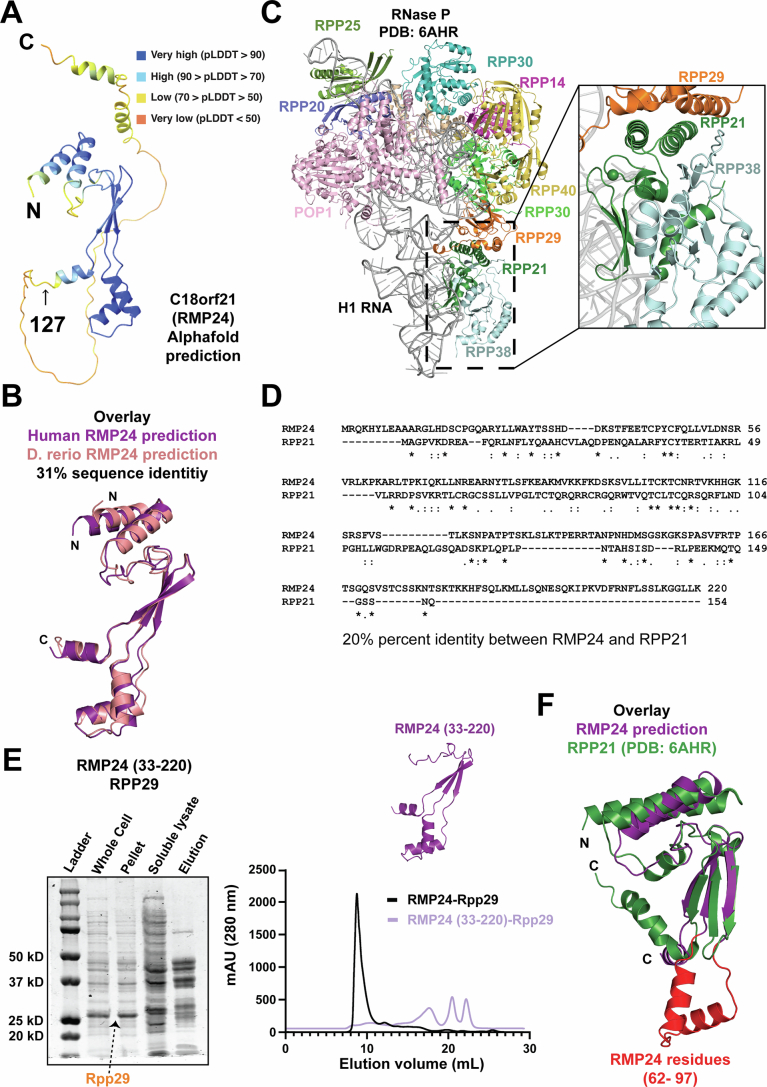
RMP24 is predicted to have structural homology with RPP21. (**a**) The predicted structure of RMP24 shown in a cartoon representation. The structure is colored by the predicted local distance difference test (pLDDT) confidence score acquired from the AlphaFold prediction. (**b**) An overlay of the predicted structures of RMP24 from human (deep purple) and from *Danio Rerio* (Salmon). (**c**) A cartoon representation of the solved structure of human RNase P PDB ID: 6AHR. POP5 is shown in wheat, POP1 is shown in light pink, RPP40 is shown in yellow orange, RPP38 is shown in light blue, RPP30 is shown in teal and green, RPP29 is shown in orange, RPP25 is shown in split pea green, RPP20 is shown in slate blue, RPP21 is shown in forest green, RPP14 is shown in magenta, and H1 RNA is shown in grey. The magnified view to the right of the structure highlights the contacts between RPP21 and RPP29. (**d**) Sequence alignment of human RMP24 and human RPP21 performed in Clustal omega. (**e**) A representative coomassie stained SDS-PAGE gel from two biological replicates SDS-PAGE gel showing co-purifications of RMP24 (33-220)-RPP29 complex, highlighting the insoluble complex in the lysate pellet. A representative chromatogram resulting from passing the indicated complex over a Superdex S200-increase column overlayed with the chromatogram from the RMP24-RPP29 complex purification shown in Fig. 3c. (**f**) An overlay of the predicted structure of RMP24 (deep purple) and the solved structure of human RPP21 (Forest green, PDB ID: 6AHR). Residues 62–97 of RMP24 are highlighted in red to accentuate the difference in structure between RMP24 and RPP21^61^.

To determine the basis for the interaction of RMP24 with subunits of the RNase MRP complex, we next used AlphaFold^47^ to analyze its predicted structural features. AlphaFold confidently predicted that the N-terminal domain (residues 1–127) of RMP24 folds into an elongated structure with three anti-parallel β-strands sandwiched between two sets of α-helices, whereas the RMP24 carboxy-terminal domain is predicted to be largely unstructured (Fig. 3a and Extended Data Fig. 3a). AlphaFold predictions of RMP24 structures from a variety of organisms suggest that this N-terminal domain is structurally conserved, even in cases with low sequence similarity (Extended Data Fig. 3b). Because our data suggest that RMP24 is a member of the RNase MRP complex, we used AlphaFold 3 to predict how RMP24 fits into the larger enzyme complex (Fig. 3b). In this structural model, RMP24 is predicted to be sandwiched between RPP29 and RPP38, with the two N-terminal helices of RMP24 forming a protein–protein interface with RPP29 (Fig. 3b). To test this structural model directly, we tested whether RMP24 interacts with RPP29 in vitro. When expressed on its own in bacteria, the RMP24 protein was insoluble. However, upon coexpression of RMP24 and RPP29 in bacteria, we could isolate a stable RMP24–RPP29 dimer, illustrating a direct interaction between the two proteins (Fig. 3c).

Inspection of the previously solved structure of human RNase P shows that the RNase-P-specific protein RPP21 similarly uses its two N-terminal helices to interact directly with RPP29 (Extended Data Fig. 3c). Alignment of the structure of RPP21 and the predicted structure of RMP24 revealed that the N-terminal domains of RMP24 (residues 1–127) and RPP21 (4–124) share many structural features, despite the fact that RMP24 and RPP21 display only 20% sequence identity (Fig. 3d and Extended Data Fig. 3d). Each protein has a pair of N-terminal helices that form a hydrophobic surface, suggesting that each protein interacts with RPP29 in a similar manner. To further test this model, we coexpressed an RPP29–RMP24 construct, in which this pair of helices was removed (RMP24^33–220^), in bacteria. In contrast to what occurred during coexpression with full-length RMP24, RPP29 was insoluble, preventing isolation of a RMP24^33–220^–RPP29 complex (Extended Data Fig. 3e). This suggests that this region of RMP24 is necessary for interaction with RPP29. In addition to the similar N-terminal α-helices that are present in both proteins, RPP21 and RMP24 both have three anti-parallel β-sheets at their N termini. Specifically, residues 1–61 and 98–115 of the predicted RMP24 structure closely matched the structure of residues 19–108 of RPP21 (Fig. 3d and Extended Data Fig. 3f). Thus, our analysis suggests that RMP24 is related to RPP21, and these two proteins act as mutually exclusive subunits of the RNase MRP/P complexes.

### Specificity for the RNase MRP or RNase P complex is dictated by the N terminus of RMP24 and RPP21

**Fig. 4:**
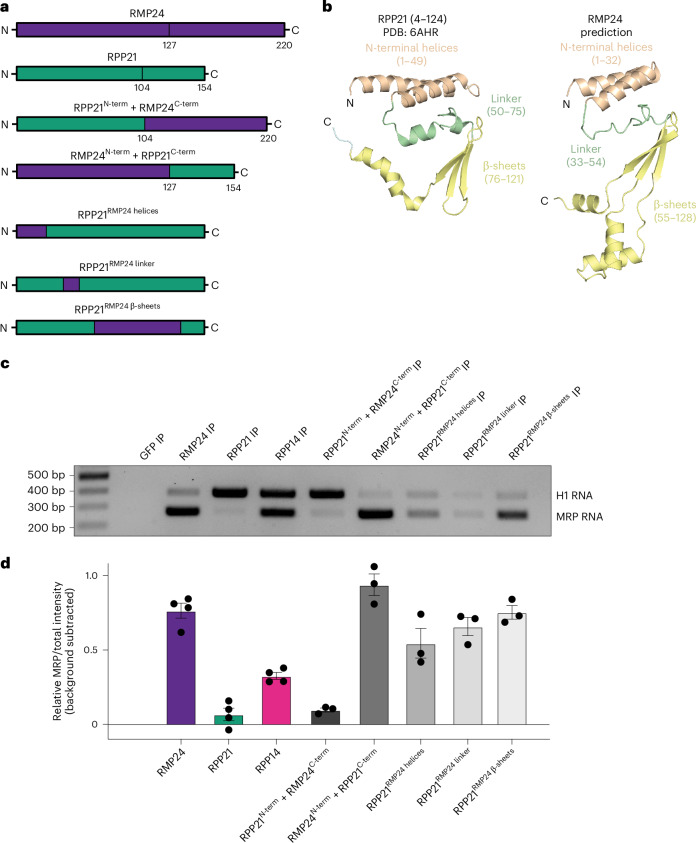
Specificity for RNase MRP or RNase P is dictated by the N termini of RPP21 and RMP24. **a**, Diagram depicting the chimeric constructs used in **c**. **b**, A cartoon representation of the solved structure of human RPP21 (PDB ID: 6AHR) and the predicted structure of RMP24. Each of the three shared structural features is depicted in a different color. **c**, Representative agarose gel stained with ethidium bromide from three biological replicates, showing the results of RIP–RT–PCR experiments. **d**, Quantitation of the integrated pixel intensity of the RNase MRP RNA signal with the GFP signal background subtracted. The *y* axis shows the fraction of the intensity of MRP RNA to the sum of intensities for both H1 RNA and MRP species. The quantitation for the RMP24, RPP21 and RPP14 RIP–RT–PCRs is duplicated from Fig. 2g for comparison with the chimeras. The error bars represent the s.e.m. from four replicates for the RMP24, RPP21 and RPP14 experiments, and three replicates for the chimera experiments.

To understand the molecular determinants that allow RPP21 and RMP24 to share structural features, but act as mutually exclusive subunits of the RNase P and RNase MRP complexes, we generated HeLa cell lines that ectopically expressed C-terminally GFP-tagged chimeras of RPP21 or RMP24 (Fig. 4a,b). Because the major differences in the predicted secondary structure of the two proteins are present in their C-terminal domains, we first generated chimeric proteins in which we fused the N-terminal domain of RPP21 to the C-terminal domain of RMP24 (RPP21^N-term^–RMP24^C-term^) or the N-terminal domain of RMP24 to the C-terminal domain of RPP21 (RMP24^N-term^–RPP21^C-term^) (Fig. 4a). In RIP–RT–PCR experiments, we found that the RPP21^N-term^–RMP24 ^C-term^ construct isolated H1 RNA, whereas the RMP24^N-term^–RPP21^C-term^ construct interacted with RNase MRP RNA (Fig. 4c,d). Despite the predicted similarities in the N-terminal domains of the RPP21 and RMP24 structures, these results suggest that these domains are likely to confer specificity for RNase P versus RNase MRP.

**Extended Data Fig. 4:**
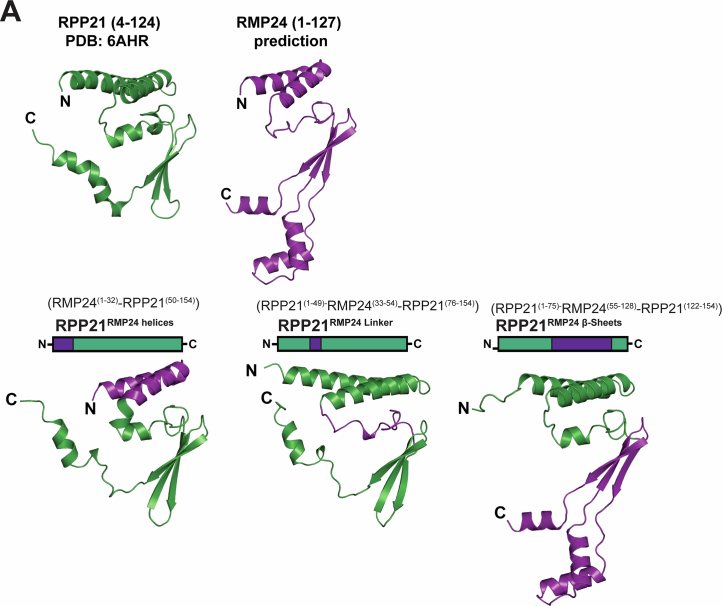
Structural predictions of chimeric RMP24/RPP21 proteins. (**a**) Structural predictions for each chimeric construct used in Fig. 4. Structural elements taken from RMP24 are shown in purple and structural elements taken from RPP21 are shown in green. Each prediction was done with the full-length construct, for simplicity, only the conserved N-terminal domain is depicted.

To define regions in the N-terminal domain of RMP24 that confer RNase MRP complex specificity, we tested RPP21–RMP24 chimeras, in which a structurally homologous feature was swapped from RMP24 onto RPP21 (Fig. 4a,c,d and Extended Data Fig. 4a). Surprisingly, we found that all three RMP24 structural features, when swapped into RPP21, immunoprecipitated both H1 and MRP RNA (Fig. 4c,d). However, the chimeric RPP21 construct in which we swapped the linker region of RPP21 for the linker region of RMP24 (RPP21^RMP24 linker^) was inefficient when pulling down either RNA. We found that the RPP21^RMP24 β-sheets^ construct pulled down more MRP RNA than H1 RNA, similar to the activity of wild-type RMP24. This result suggests that this region in RMP24 is sufficient to confer specificity for MRP RNA (Fig. 4c,d). Together, these results show that substituting any one of the structural features in the N-terminal domain of RPP21 with the corresponding structural feature of RMP24 can confer interaction with RNase MRP RNA.

### RNase MRP preferentially affects 40S ribosome biogenesis

**Fig. 5:**
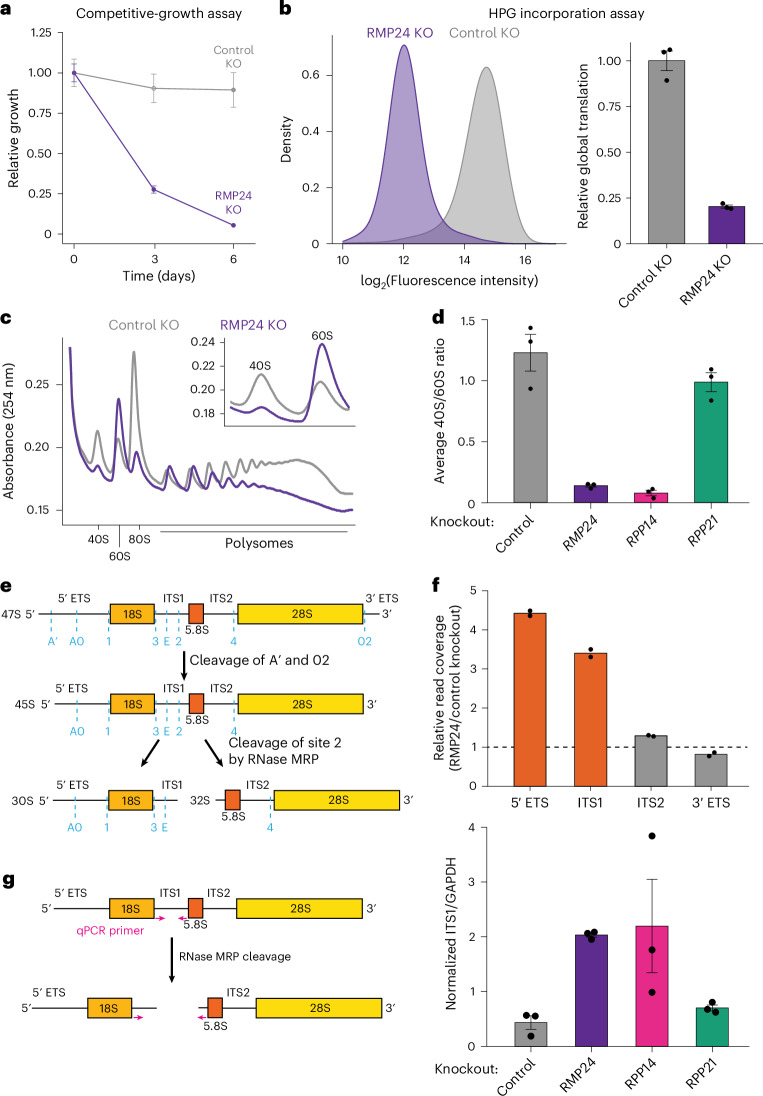
Loss of RMP24 leads to defects in ribosome biogenesis. **a**, A plot showing the relative growth of either RMP24-knockout cells (purple) or control knockout cells (gray) relative to control cells over time. Error bars indicate the s.e.m. from three biological replicates. **b**, A histogram generated by measuring the fluorescence intensity of HPG incorporated cells by flow cytometry (left). The average HPG signal in each condition is quantified. The error bars represent the s.e.m. from three biological replicates, and statistics were derived from Student’s *t*-test (*P* = 0.0001). **c**, An overlay of the polysome profiles obtained from the fractionation of cell lysates separated on a 10–50% sucrose gradient. The inset on the top right shows a magnified view of the 40S and 60S traces. **d**, Quantification of the ratio of 40S to 60S ribosomes obtained from polysome gradients, with the indicated gene knocked out by CRISPR–Cas9 targeting. The error bars represent the s.e.m. from three biological replicates. **e**, Schematic outline of the primary rRNA maturation pathway in HeLa cells^3^. **f**, Quantitation of RNA-seq data showing the relative read coverage of different domains of the 45S rRNA transcript obtained from RNA isolated from RMP24-knockout cells and HS1-knockout control cells. Each point represents a single biological replicate. **g**, Left, schematic showing the qPCR scheme to determine the abundance of uncleaved ITS1. Right, quantitation of the ITS1 signal normalized to the GAPDH signal from qPCR experiments performed on RNA extracted from the indicated knockout cell lines. The error bars represent the s.e.m. from three biological replicates. Two-sided Student’s *t*-tests were used for statistical analysis.

**Extended Data Fig. 5:**
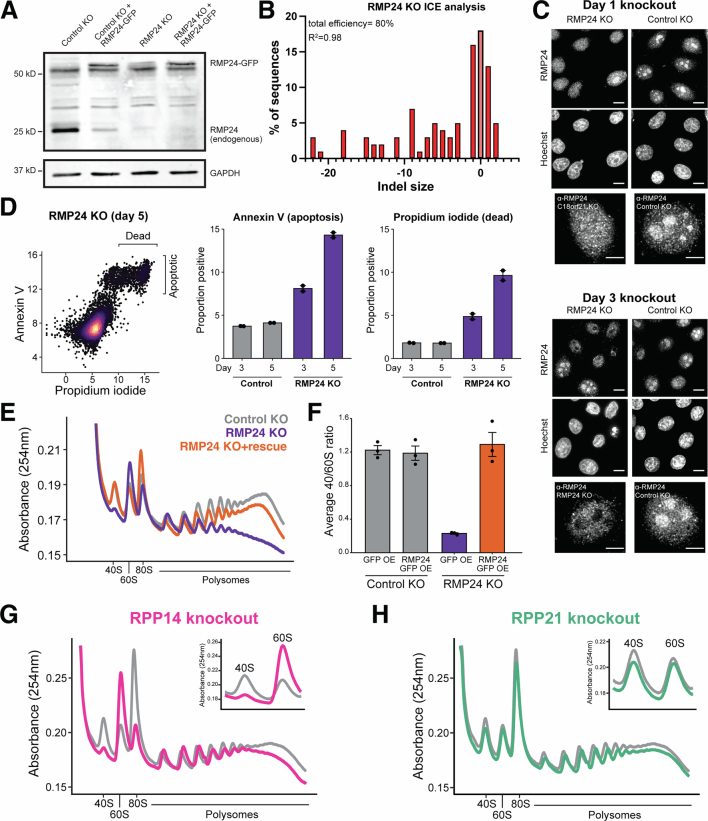
Loss of RMP24 induces apoptosis and leads to defects in ribosome biogenesis. (**a**) A representative western blot from two biological replicates showing the expression levels of RMP24 in the indicated cell lines. (**b**) Sanger sequencing-based ICE (Synthego) analysis^79^ of genomic DNA extracted from HeLa cells three days after RMP24 knockout. The y-axis depicts the frame of the indel and the x-axis depicts the percentage of sequences with that frame. (**c**) Representative immunofluorescence images from three biological replicates taken of either control or RMP24 knockout cells stained with a polyclonal RMP24 (C18orf21) antibody. The scale bar represents 20 µm in the zoomed-out view and 5 µm in the zoomed-in view. (**d**) *Left:* A representative scatter plot from two biological replicates showing propidium iodide (x-axis) and annexin V (y-axis) staining after five days of RMP24 knockout. Dead/apoptotic cells are highlighted with brackets. *Middle:* A bar graph showing the proportion of positive annexin V stained cells after 3 or 5 days of either control or RMP24 knockout. *Right:* A bar graph showing the proportion of positive propidium iodide stained cells after 3 or 5 days of either control or RMP24 knockout. (**e**) Representative polysome profile obtained from three biological replicates from the fractionation of cell lysates obtained from cells where a RMP24 construct hardened against the sgRNA with a C-terminal GFP was ectopically expressed in RMP24 knockout cells. The traces for control and RMP24 knockout cells are taken from Fig. 5c for comparison with the ectopic rescue. (**f**) Quantitation of the 40S to 60S ribosomal subunit ratio in the indicated conditions from experiments performed in triplicate. The error bars represent the standard error of the mean. (**g**) Representative polysome profile from three biological replicates obtained from the fractionation of cell lysates obtained from RPP14 knockout cells. The inset in the top right is zoomed in to highlight the 40S to 60S ribosome ratio. (**h**) Representative polysome profile from three biological replicates obtained from the fractionation of cell lysates obtained from RPP21 knockout cells. The inset in the top right is zoomed in to highlight the 40S to 60S ribosome ratio.

The identification of RMP24 as an RNase-MRP-specific subunit creates the opportunity to assess the functional contributions of RNase MRP. The precise function of RNase MRP in human cells has been difficult to define owing to the lack of a specific protein component. Earlier work relied on transient knockout of the RNase MRP RNA to assess of RNase MRP function, relying on a dual guide system with relatively low penetrance^8^. To define the functional contributions of RMP24, we tested how eliminating the gene affected cell growth, translation, ribosome biogenesis and rRNA processing. On the basis of a competitive growth assay using a coculture system (Methods), we found that CRISPR–Cas9-based targeting of RMP24 led to a substantial reduction in cellular fitness relative to that of control cells (Fig. 5a and Extended Data Fig. 5a–c). This decreased fitness was due, at least in part, to an increased rate of apoptosis and cell death (Extended Data Fig. 5d), consistent with prior work showing that RNase MRP RNA is essential in yeast and human cells^8,22^.

Because RNase MRP is predicted to contribute to ribosome production, we compared rates of protein synthesis using an incorporation assay of L-homopropargylglycine (HPG; a clickable methionine analog^48^). In RMP24-knockout cells, global translation rates were much lower than those of controls (Fig. 5b). Consistent with this assay, RMP24-knockout HeLa cells also displayed a decreased polysome/monosome ratio compared with control knockout cells (Fig. 5c). Surprisingly, we found that the fraction of mature 40S ribosomes was depleted, whereas the number of free 60S subunits increased in RMP24-knockout cells compared with control HeLa cells (Fig. 5c,d). Consistent with the notion that RMP24 has a preferential role in 40S-ribosome biogenesis, recent genome-wide perturbation studies have clustered RMP24 with 40S, rather than 60S, ribosome subunits^42^. We found that this decrease in the abundance of the mature 40S ribosome could be rescued by ectopic expression of RMP24 (Extended Data Fig. 5e,f). In addition, we found that the knockout of RPP14 (RNase MRP/P), but not RPP21 (RNase P), resulted in a similar decrease in 40/60S ribosome ratio (Fig. 5d and Extended Data Fig. 5g,h). Thus, this unexpected defect in the ratio of mature ribosome species is specific to eliminating RNase MRP activity.

**Extended Data Fig. 6:**
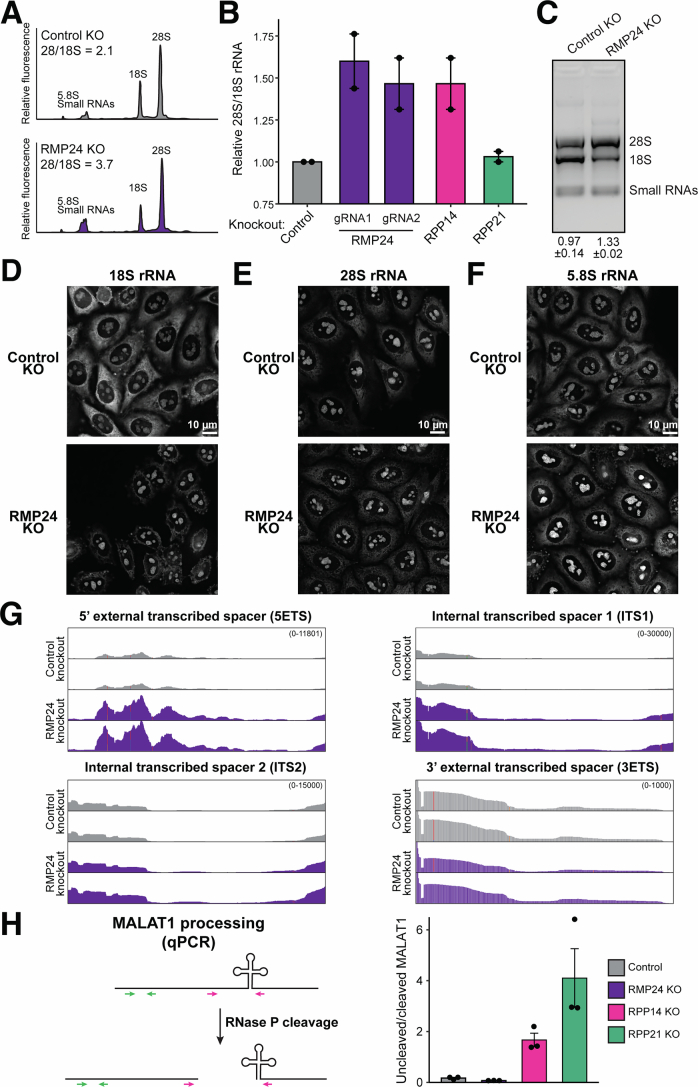
Loss of RMP24 leads to defects in rRNA biogenesis. (**a**) Representative bioanalyzer traces from two biological replicates obtained from RNA isolated from control or RMP24 knockout cells. Ratios of 28S:18S were obtained by integrating each peak in the bioanalyzer trace. (**b**) Quantitation of the 28S:18S rRNA ratio taken from duplicate experiments in the indicated knockout cell lines. (**c**) A representative SYBR-gold stained agarose gel depicting the RNA species present in control or RMP24 knockout cells. The ratio of the intensity of the 18S rRNA band relative to the 28S rRNA band is shown at the bottom of the gel. The standard error of the mean was calculated from two biological replicates. (**d**)**–**(**f**) Representative fluorescence *in situ* hybridization images from two biological replicates of control or RMP24 knockout cells performed with Cy5 labeled probes for the 18S, 5.8S, or 28S rRNA species. The control and RMP24 cell images are uniformly scaled for each rRNA probe, but the images are scaled differently across the probes. (**g**) RNA-sequencing coverage plots that display reads for specific regions of the 45S rRNA from the RMP24 knockout cells or control knockout cells. The red and green bars represent mutations identified in the reads for the indicated region. (**h**) *Left:* schematic showing the qPCR scheme to determine the abundance of uncleaved MALAT1. *Right:* Quantitation of signal from primers that amplify the 3’ end of MALAT1^81,82^ normalized to the signal obtained from primers that amplify the 5’ end of MALAT1 (total MALAT1 levels) from RT-qPCR experiments performed on RNA extracted from the indicated knockout cell lines. The error bars represent the standard error of the mean from 3 replicates.

The cleavage activity of RNase MRP separates the 18S rRNA subunit from the 5.8S and 28S rRNA subunits, facilitating the maturation of each rRNA subunit (Fig. 5e). To test whether the changes in ribosomal subunit population were due to changes in rRNA processing, we compared the abundance of rRNA species in control cells and in RPP21-, RMP24- and RPP14-knockout cells (Extended Data Fig. 6a–f). Owing to its RNase-P-specific role, we found that there was no difference in the ratio of mature 18S rRNA species, relative to 28S rRNA species, between control and RPP21-knockout cells (Extended Data Fig. 6b). We found that, consistent with a role in RNase MRP complex function, RMP24- and RPP14-knockout cells each had decreased amounts of mature steady-state 18S rRNA relative to 28S rRNA (Extended Data Fig. 6a–c). We also monitored the nucleolar and cytoplasmic distribution of the rRNA species by fluorescence in situ hybridization (FISH) (Extended Data Fig. 6d–f). In agreement with a change in the ratio of mature rRNA, we found that RMP24-knockout cells displayed reduced 18S rRNA levels in the cytoplasm and an accumulation of nucleolar signal compared to control cells (Extended Data Fig. 6d). In contrast, there was no difference in the distribution of 28S rRNA in either condition (Extended Data Fig. 6e). Knockout of RMP24 and RPP14 also resulted in the increase in rRNA intermediates, particularly upstream of the 5.8S rRNA, including an accumulation of 5.8S rRNA signal in the nucleolus in RMP24-knockout cells (Fig. 5f and Extended Data Fig. 6f,g). The analysis of RMP24-knockout cells is consistent with prior work showing that that targeting RNase MRP RNA with CRISPR–Cas9 leads to accumulation of the 5.8S rRNA in the nucleolus and the formation of rRNA processing intermediates, called X and 21SL3’, that contain 18S rRNA, ITS1 and 5.8S rRNA, but not regions downstream of 5.8S, including the 28S rRNA^8^. By contrast, work in yeast has shown that base pairing between the 5.8S and 25S rRNAs precede ITS2 cleavage, suggesting that 28S and 5.8S should exhibit matching nucleolar levels^49,50^. However, 28S does not accumulate in the nucleolus of RMP24-knockout cells (Extended Data Fig. 6e). Working out whether this discrepancy is due to technical factors in our FISH experiment, or a biological difference between human and yeast, will require further studies of rRNA maturation in humans. To test whether RMP24 knockout was specific to the loss of activity of RNase MRP, but not RNase P, we measured RNase P activity through MALAT1 cleavage with RT–quantitative PCR (RT–qPCR) (Extended Data Fig. 6h). Knockout of RMP24 did not affect MALAT1 cleavage, whereas knockout of RPP21 and RPP14 decreased MALAT1 processing (Extended Data Fig. 6h), consistent with RMP24 being uniquely a part of RNase MRP.

**Extended Data Fig. 7:**
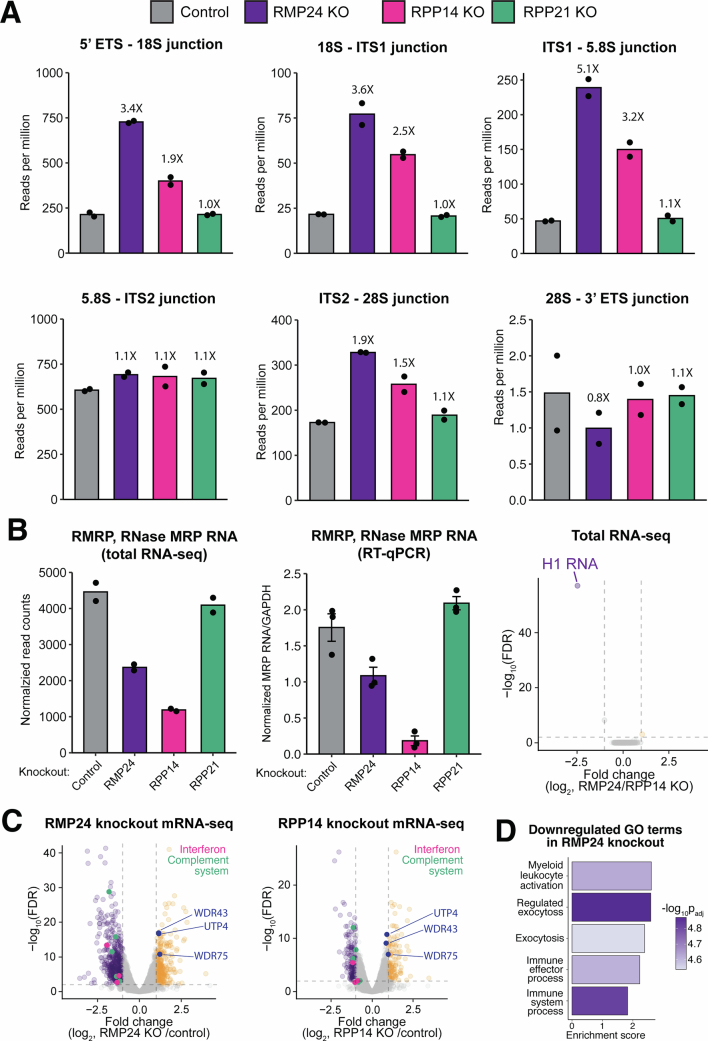
Loss of RMP24 and RPP14 leads to changes in rRNA processing, RNase MRP levels, and gene expression. (**a**) Quantitation of RNA sequencing data showing the relative read coverage of the indicted spacer-rRNA junctions obtained from RNA isolated from RMP24, RPP14, RPP21, or HS1 control knockout cells. (**b**) *Left:* Quantitation of RNA sequencing data showing the relative read coverage of RNase MPR RNA (*RMRP*) *Middle:* Quantitation of the RNase MRP RNA signal normalized to the GAPDH signal from qPCR experiments performed on RNA isolated from RMP24, RPP14, RPP21, or HS1 control knockout cells. The error bars represent the standard error of the mean from 3 replicates. *Right:* Volcano plot showing the changes in gene expression in RMP24 knockout compared to RPP14 knockout cells. Genes with FDR < 0.01 and a fold change > 2 were called as significant. (**c**) *Left:* Volcano plot showing the changes in gene expression in RMP24 knockout compared to control knockout cells. *Right:* Volcano plot showing the changes in gene expression in RPP14 knockout compared to control knockout cells. For both volcano plots genes with FDR < 0.01 and a fold change > 2 were called as significant. (**d**) GO term enrichment analysis of RMP24 knockout cells to identify gene sets that are downregulated upon RMP24 knockout. Gorilla^83^ was used to GO term analysis with default settings. FDR was used to correct for multiple hypothesis testing.

To test whether the changes in levels of relative mature rRNA species were caused by changes in cleavage of site 2, we carried out RT–qPCR experiments with primers flanking this region. We found that both RMP24- and RPP14-knockout cells had an increased abundance of unprocessed ITS1 compared with control and RPP21-knockout cells (Fig. 5g). The change in ITS1 cleavage is consistent with the observation that RMP24- and RPP14-knockout cells have an increase in reads spanning the junction created by ITS1 and 5.8S rRNA, as well as the junctions flanking the 18S rRNA (Extended data Fig. 7a). This result is also consistent with previous studies showing that subunit loss in RNase MRP led to the accumulation of precursor rRNA species^8,51^. In addition to changes in rRNA processing, we found that loss of RMP24 or RPP14 led to a decrease in abundance of RNase MRP RNA (Extended data Fig. 7b), suggesting that these subunits are required for RNase MRP stability or expression. Previous work has suggested that RNase MRP cleavage at site 2 in ITS1 is a step in the predominant pathway for the production of 18S, 5.8S and 28S rRNA (Fig. 5e)^3^. Surprisingly, we found that the loss of RMP24 and RPP14 preferentially affects 18S rRNA production (Extended data Fig. 6a–f), consistent with the specific loss of mature 40S ribosomes (Fig. 5c,d). This suggests that loss of either component leaves RNase MRP partially functional, or an alternative rRNA-processing pathway exists, such as those that are found in *Saccharomyces cerevisiae*, that bypasses loss of RNase MRP to generate 5.8S and 28S rRNA^52,53^.

In addition to changes in rRNA levels, we also assessed the transcriptome of cells lacking RNase MRP activity (RMP24 knockout) in HeLa cells. RMP24 knockouts displayed strong changes in the levels of hundreds of mRNAs (Extended Data Fig. 7c). These included the upregulation of several rRNA-processing components, such as UTP4, WDR75 and WDR43, which could represent compensatory mechanisms upon the loss of RNase MRP activity (Extended Data Fig. 7c). We also observed hundreds of mRNAs downregulated upon RMP24 knockout, including interferon and complement-system genes (Extended Data Fig. 7c,d). By analyzing the transcriptome of RPP14-knockout cells, we found similar changes in gene expression (Extended Data Fig. 7c), suggesting that this transcriptomic signature likely represents an RNase-MRP-defective signature. The gene-expression changes in RNase-MRP-deficient cells could be related to how RNase MRP diseases are associated with immunodeficiencies^26–28^. Together, the results of our functional analysis indicate that the RNase-MRP-specific protein RMP24 is essential for cell viability and has key roles in translation and 40S-ribosome biogenesis, and provides insights on how loss of RNase MRP activity alters the transcriptome.

### NEPRO/RMP64 associates with the RNase MRP complex

**Fig. 6:**
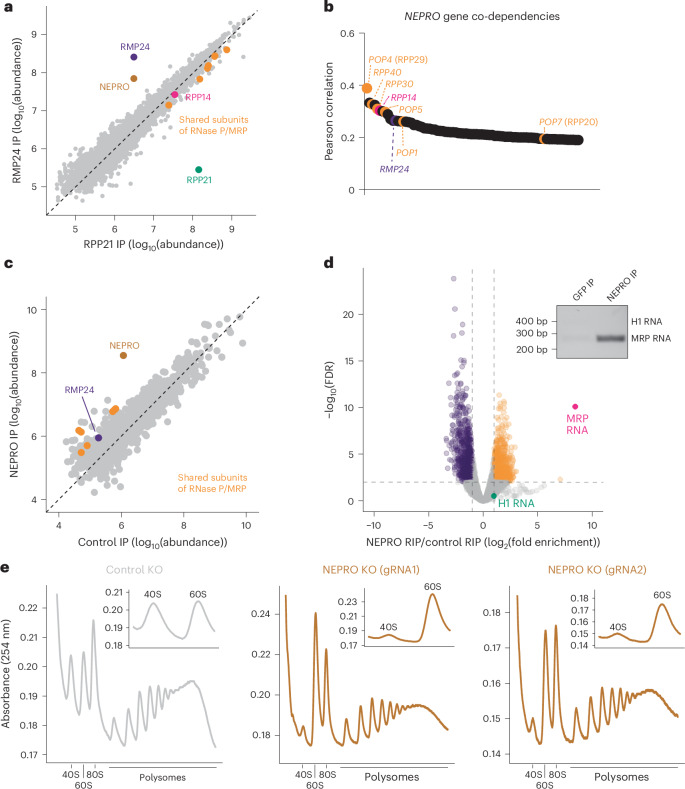
NEPRO is an rRNA-processing factor that associates with RNase MRP. **a**, The abundance of the proteins detected in the indicated IP–MS experiments. The shared components of the RNase P/MRP complexes are highlighted in orange, except RPP14, which is shown in pink. RMP24 is highlighted in purple, NEPRO is highlighted in brown and RPP21 is highlighted in green. **b**, The genes with the highest Pearson correlation of CRISPR–Cas9-based targeting effect scores to *NEPRO* in the DepMap database (DepMap Public 24Q2). The shared RNase P/MRP components are shown in orange, except RPP14, shown in pink, and RMP24, shown in purple. **c**, The abundance of the proteins detected in the indicated IP–MS experiments. The shared components of the RNase P/MRP complexes are highlighted in orange. RPP21 was not immunoprecipitated in the NEPRO IP–MS experiments, and thus is not shown. **d**, A volcano plot showing the log_10_(FDR) and fold enrichment from RIP–RNA-seq experiments. The top right inset depicts a representative agarose gel stained with ethidium bromide, showing the results of NEPRO RIP–RT–PCR experiments, performed in triplicate. **e**, Polysome profiles obtained from the fractionation of cell lysates separated on a 10–50% sucrose gradient. NEPRO-knockout profiles obtained using two different guide RNAs are depicted in brown, and the control profile is depicted in gray. The inset on the top right shows a magnified view of the 40S and 60S traces.

**Extended Data Fig. 8:**
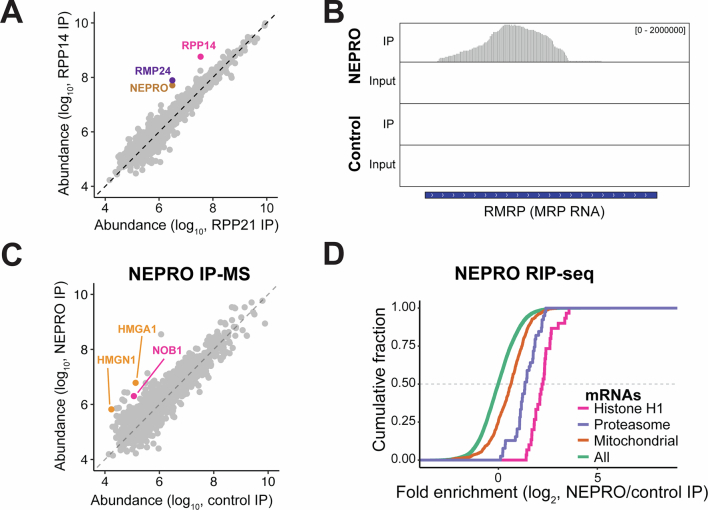
NEPRO associates with additional proteins and RNAs not found in the RNase MRP complex. (**a**) A Scatter plot showing the abundance of the proteins detected in the indicated immunoprecipitation-mass spectrometry experiments. RPP14 is shown in pink, RMP24 is highlighted in purple, and NEPRO is highlighted in brown. (**b**) RNA-sequencing coverage plots that display reads for RNase MRP RNA. (**c**) The same scatter plot that is shown in Fig. 6c highlighting non-RNase MRP/P proteins that were immunoprecipitated by NEPRO. (**d**) Plot showing the cumulative fraction on the Y-axis and the log_2_ fold enrichment on the X-axis of families of mRNA species obtained from ribonucleoprotein-immunoprecipitation (RIP) RNA sequencing experiments. Mitochondrial gene annotations were taken from MitoCarta3.0^84^.

The discovery of RMP24 as a unique member of the RNase MRP complex enables proteins that interact with the RNase MRP complex, but not the RNase P complex, to be identified. To evaluate whether there are any such proteins, we compared proteins that interact with RPP21 and RMP24 in our IP–MS experiments. The protein NEPRO (C3orf17) stands out as a unique interacting partner for both RMP24 and RPP14, but not RPP21 (Fig. 6a and Extended Data Fig. 8a). On the basis of the DepMap database^41^, *NEPRO* is also co-essential in cancer cells with several of the shared RNase P/MRP complex components and the RNase-MRP-specific subunit *RMP24* (Fig. 6b). Similarly, *NEPRO* clusters with factors involved in 40S ribosome biogenesis in both genome-wide Perturb-Seq and a pooled optical CRISPR–Cas9-based functional screen^42,54^.

To determine NEPRO’s interacting partners, we conducted IP–MS experiments in HeLa cells ectopically expressing a C-terminally-tagged NEPRO–GFP fusion. We found that NEPRO interacts with the shared components of the RNase P/MRP complex and the RNase-MRP-specific protein RMP24, but not the RNase-P-specific protein RPP21 (Fig. 6c and Supplementary Table 1). This observation is supported by previous work showing that NEPRO can interact with several of the shared RNase P/MRP components^45,55^. To determine whether NEPRO has specificity for either H1 RNA or RNase MRP RNA, we performed GFP immunoprecipitations followed by RNA sequencing and RT–PCR. NEPRO–GFP immunoprecipitation isolated RNase MRP RNA, but not H1 RNA, suggesting that NEPRO associates with the RNase MRP complex, but not the RNase P complex (Fig. 6d, Extended Data Fig. 8b and Supplementary Table 2). To test whether NEPRO loss and RMP24 or RPP14 loss lead to a similar defect in ribosome biogenesis, we carried out polysome profiling in HeLa cells in which NEPRO was targeted by CRISPR–Cas9. Upon NEPRO depletion, we observed a decrease in the population of mature 40S ribosomes, mirroring the decrease observed when RMP24 or RPP14 was knocked out (Fig. 6e). Together, our data show that NEPRO associates with RNase MRP complex and has a key role in ribosome biogenesis. On the basis of the protein and RNA interactions, genetic associations and subcellular localizations, we propose that NEPRO is a subunit of RNase MRP and refer to it as ribonuclease MRP subunit p64 (RMP64).

Previous work highlighted multiple additional roles for RMP64 across different cell types. For example, RMP64 is activated downstream of Notch signaling to inhibit differentiation of neurons, and loss of RMP64 leads to the mis-localization of ribosomal subunits and mitochondrial proteins at the two-cell stage in mouse embryos^56,57^. In agreement with roles for RMP64 outside the RNase MRP complex, we found that RMP64 interacts with other rRNA-processing factors in our IP–MS experiments that do not immunoprecipitate with RMP24 or RPP14 (Extended Data Fig. 8c). Additionally, we found that RMP64 pulls down several proteins that are associated with transcription regulation (Extended Data Fig. 8c). Similarly, using RIP-seq, we found that RMP24, RPP14 and RMP64 interact with RNase MRP RNA. However, in contrast to RMP24 and RPP14, which did not interact with any mRNAs (Fig. 2c,f), we found that RMP64 significantly interacts with 875 mRNAs, including the many histone H1 subtypes, mRNAs of proteasome subunits and nuclear-encoded mitochondrial mRNAs, highlighting potential roles outside of rRNA processing for this protein (Fig. 6d and Extended Data Fig. 8d). Together, our findings indicate that RMP64 is a subunit of the RNase MRP complex, but not RNase P. However, our work also suggests that RMP64 could associate and function with unrelated complexes.

### Disease-causing substitutions in NEPRO/RMP64 lead to altered RNase MRP association

**Fig. 7:**
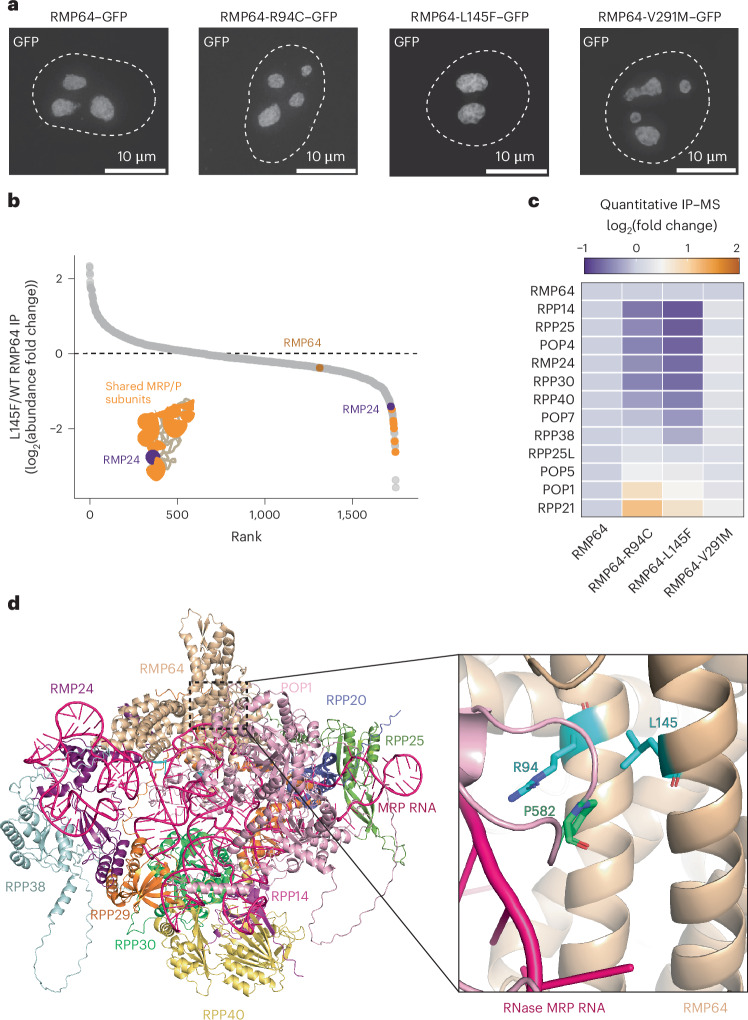
Disease-associated substitutions in RMP64 disrupt its interaction with RNase MRP. **a**, Representative Z-projected images from more than three biological replicates taken during live imaging of cells ectopically expressing the indicated C-terminally GFP-tagged NEPRO–RMP64 constructs. The dashed outlines represent the boundaries of the nucleus, which were obtained from Hoechst staining. Images were deconvolved and are not scaled equally. **b**, The abundance fold change of proteins between RMP64-L145F and RMP64 immunoprecipitations. **c**, A heat map showing the average fold change in abundance from two biological replicates of each RNase P/MRP component immunoprecipitated from cell lines ectopically expressing the indicated GFP-tagged construct. **d**, A cartoon representation of the predicted structure of human RNase MRP with RMP64, created using AlphaFold 3 (ref. ^47^). The magnified view to the right of the full structure highlights the predicted contacts between RMP64 and POP1. Residue L145 of RMP64 is shown in cyan in a stick representation. Residues 580, 581 and 582 of POP1 are shown in a stick representation and are shown in light pink.

Substitutions in the promoter region and several subunits of RNase MRP can cause a variety of diseases, including anauxetic dysplasia^23–29^. Notably, people with anauxetic dysplasia have been found to have missense mutations affecting residues R94 and L145 of RMP64, consistent with RMP64 being a subunit of RNase MRP^58^. We first assessed the ability of RMP64-R94C and RMP64-L145F to localize to the nucleolus. Using live-cell imaging, we found that RMP64-R94C–GFP and RMP64-L145F–GFP localized to the nucleolus, similar to wild-type RMP64 (Fig. 7a). Next, to test for differences in protein interactions, we profiled the partners of RMP64-R94C and RMP64-L145F by quantitative IP–MS. Both substitutions displayed reduced interactions with components of the RNase–MRP complex (Fig. 7b,c and Supplementary Table 1). By contrast, other RMP64 interacting partners were immunoprecipitated equally by the wild-type and mutant protein (Supplementary Table 1). This suggests that the p.R94C and p.L145F substitutions predominantly affect the association of RMP64 with RNase MRP (Fig. 7b).

**Extended Data Fig. 9:**
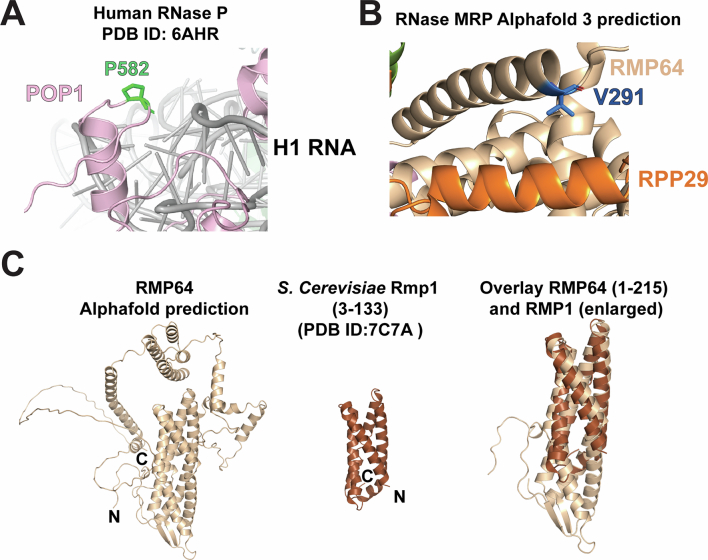
NEPRO has structural homology with Rmp1 from *S. Cerevisiae.* (**a**) A magnified view of the structure of human RNase P (PDB ID:6AHR) with POP1 shown in light pink. Residue P582 is shown in a stick representation in green^61^. (**b**) A magnified view of the predicted structure of RNase MRP highlights the RMP64 and RPP29 interaction. Residue V291 is shown in a stick representation in marine blue. This residue is mutated in a patient with Stickler syndrome. (**c**) A cartoon representation of the predicted structure of RMP64 that was created using AlphaFold 3 is depicted in wheat and the solved structure of *S. cerevisae* Rmp1 (PDB ID: 7C7A) shown in brown. An overlay of the structurally similar N-terminal helical bundle is shown to the right.

To model how the RMP64-R94C and RMP64-L145F mutants affect the interaction between RMP64 and the RNase MRP complex, we used AlphaFold 3 to predict the structure of RMP64 with all of the components of the RNase MRP complex (Fig. 7d). We found that both the R94 and L145 residues of RMP64 are predicted to be at an interface formed between RMP64 and POP1, providing a molecular explanation for the disease caused by this substitution (Fig. 7d). Surprisingly, a missense mutation associated with anauxetic dysplasia in POP1 encodes p.P582S, found at this same interface, suggesting that this surface is important for RNase MRP complex formation (Fig. 7d)^25,59,60^. This is highlighted by the fact that the solved structure of RNase P places residue 582 of POP1 in a linker that faces the solvent, suggesting that p.P582S would likely affect only the RNase MRP complex, consistent with this disease also being associated with MRP RNA, but not H1 RNA, substitutions (Extended Data Fig. 9a)^61^. Additional work has identified a missense mutation in RMP64 (encoding p.V291M) in an individual with a connective-tissue disorder called Stickler syndrome^62^. Our structural model places residue V291 at an interface with RPP29 (Extended Data Fig. 9b). However, this substitution does not affect either protein localization or the interaction between RMP64 and the other subunits of the RNase MRP complex (Fig. 7a,c). Together, our data indicate that RMP-R94C and RMP64-L145F exhibit altered interactions with the RNase MRP complex, providing a molecular explanation for the disease caused by this substitution. Further, our structural model shows that, despite being located at distinct regions of the complex, several anauxetic-dysplasia-causing substitutions cluster to the same surface between POP1 and RMP64, defining a hotspot for disease-causing mutations in the structure of RNase MRP.

## Discussion

**Extended Data Fig. 10:**
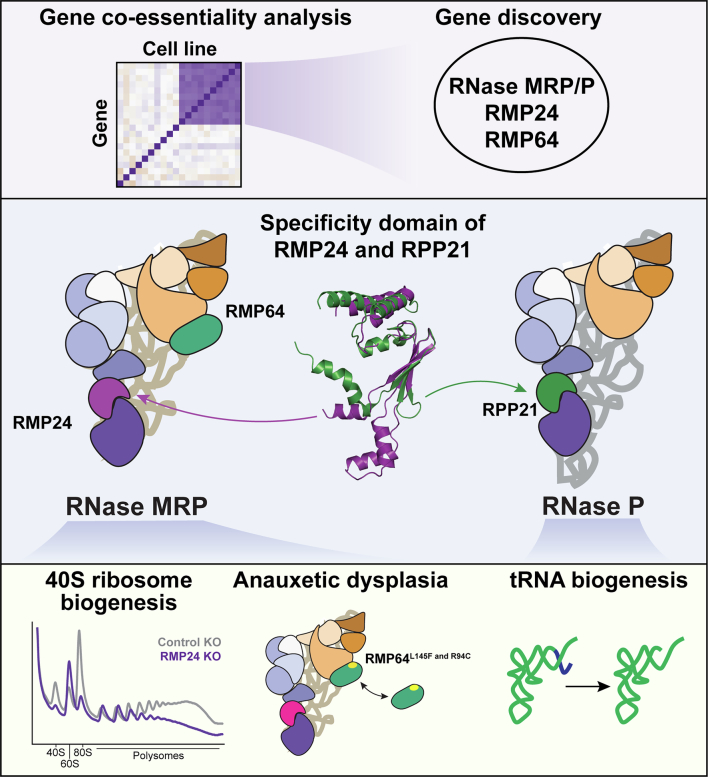
RNase MRP has two unique subunits that are required for 40S ribosome biogenesis. Schematic model of the molecular determinants of RNase MRP specificity and function in homeostasis and disease.

Despite the distinct roles of RNase MRP and RNase P in cellular function, they share multiple protein components, and it was unknown whether any unique RNase-MRP-specific proteins existed in humans. To address this, we identified the previously uncharacterized protein C18orf21/RMP24 as an RNase-MRP-specific protein that is conserved across vertebrates. Our work showed that loss of this protein leads to defects in cellular fitness, ribosome biogenesis and rRNA maturation, highlighting its central role in RNase MRP function. The finding that RMP24 is an RNase-MRP-specific protein led us to identify it as an additional subunit of the RNase MRP, but not RNase P, complex. However, unlike RMP24, RMP64 has several additional protein and RNA interaction partners outside of the RNase MRP complex, highlighting multiple roles for the protein aside from ribosome biogenesis. Although RMP64 performs diverse functions, we find that anauxetic dysplasia substitutions in RMP64 disrupt its association with RNase MRP (Extended Data Fig. 10). Additionally, recent work supports our findings, placing RMP24 and RMP64 as members of the RNase MRP complex^51,63^.

Our understanding of the mechanisms that RNase P uses to recognize and process tRNA is informed by several structural studies of the enzyme across all three domains of life^35,61,64,65^. By contrast, most molecular information about the enzymatic action of RNase MRP comes from studies of the enzyme from *S. cerevisiae*^37,38^. For example, the structure of *S. cerevisiae* RNase MRP bound to a 21-base-pair oligomer composed of part of the ITS1 sequence shed light on the catalytic mechanism that the enzyme uses to cleave single-stranded RNA^12^. Additionally, biochemical studies of *S. cerevisiae* RNase MRP identified the sequence features on the RNA substrate that facilitate cleavage and used cross-linking analysis to determine that POP1, POP4, POP5 and RPP1 interact with the RNA substrate^66,67^. However, no structural or biochemical experiments have identified how human RNase MRP recognizes its substrate. The studies of yeast RNase MRP have also shed light on the overall organization of the enzyme and defined how two RNase MRP specific proteins found in *S. cerevisiae*, Snm1 and Rmp1, fit into the complex^37,38^. Strikingly, Snm1 and Rmp1 both have several structural elements that match those found in our prediction of RMP24 and RMP64, but also have regions that diverge significantly (Extended Data Fig. 9c). For example, Rmp1 forms a bundle of helices at its N terminus that interact with both POP1 and the RNase MRP RNA subunit (Nme1)^11,12^. AlphaFold 3 predicts that RMP64 and Rmp1 have similar bundles of helices at their N termini, but RMP64 has 366 additional residues at its C terminus (Extended Data Fig. 9c). Although the structures of *S. cerevisiae* RNase MRP provide important models, several open questions remain about the differences between the yeast and human RNase-MRP-specific proteins, including how the human proteins fit into the enzyme. Ultimately, the identification of RMP24 and RMP64 reveal new paths to answer open questions regarding the architecture and substrate specificity of the human RNase MRP complex.

Previous work found that human RNase MRP is responsible for cleaving site 2 in ITS1 of pre-rRNA to separate the 18S rRNA from the 5.8S and 28S rRNA subunits (Fig. 5e)^8^. We found that loss of RMP24 resulted in the depletion of mature 18S rRNA species and an increase in sequencing reads in both the 5′ ETS and ITS1, indicating a build-up of rRNA-processing intermediates (Fig. 5f). Similarly, studies conducted in patient-derived fibroblast cells with a mutation in RNase MRP RNA (RMRP 70^AG^) found no change in 5.8S rRNA maturation, but did uncover an increase in the abundance of intermediate 18S rRNA species^68^. Together with previous studies, our work demonstrates that the maturation of the 18S rRNA relies strongly on RNase MRP function^8,68^. This could be through cleavage at site 2 in ITS1, but it is also possible the RNase MRP cleaves at alternative locations that are required for 18S rRNA biogenesis.

In agreement with the rRNA-processing defects that we observed, our polysome-profiling experiments show a depletion of the 40S ribosome and an accumulation of 60S ribosomes upon RMP24, RMP64 and RPP14 knockout (Figs. 5c,d and 6e). Our results strongly suggest that RNase MRP preferentially affects 40S ribosome maturation, consistent with the genetic interaction between RMP24 and 40S ribosome biogenesis factors^42^. However, prior work has suggested that RNase MRP is important for the production of 5.8S and 28S rRNA^3^. The build-up of the 60S ribosome in our experiments suggests that an alternative pathway exists that ensures 5.8S and 28S rRNA biogenesis in the absence of RNase MRP activity. It could include an unidentified redundant or alternative pathway allowing for the correct processing of the 5.8S subunit, but not the 18S rRNA. Together, our studies have leveraged the discovery of RNase MRP-specific proteins to define the contributions of this enzyme to rRNA processing and ribosome biogenesis in cells.

## Methods

### Plasmid constructs

The bicistronic cassette used to coexpress C18orf21/RMP24 and RPP29 codon was optimized for expression in *E. coli* and synthesized by Twist Bioscience. Restriction cloning was used to insert the cassette into the pGEX-6P-1 vector. Single guide RNAs (sgRNAs) were synthesized as complimentary single-stranded DNA pairs by Integrated DNA Technologies (IDT) that were annealed before being inserted into the LentiCRISPRv2-Opti vector (Addgene cat. no. 163126). RMP24, RMP64/Nepro, and RMP64-L145F, RMP64-R94C and RMP64-V291M with silent mutations resistant to the corresponding sgRNA were synthesized by Twist Bioscience. *RPP21* and *RPP14* genes were amplified from cDNA obtained from HeLa cells. The following sgRNA sequences were used: C18orf21 gRNA1 5′-GGACTGCACGACAGCTGCCC-3′; C18orf21 gRNA2 (used only in Extended Data Fig. 6b) 5′-GAGACAGAAGCACTACCTTG-3′; RPP14 5′-GGTTAGGATGTCCAAAGGTA-3′; RPP21 5′-GGTGATGGCGGGGCCGGTGA-3′; Nepro gRNA1 5′-AGTCATCAGAATTTGATGTG-3′; Nepro gRNA2 5′-GACAGTGCAGAACCCCGGCG-3′; and control HS1 5′-GCCGATGGTGAAGTGGTAAG-3′.

### Lentiviral production and transduction

Lentivirus was produced by transfecting HEK293T cells with 1.2 µg lentiCas9v2-Opti vector containing a sgRNA, 1 µg psPAX2 (Addgene 12260) and 0.4 µg vsFULL (Gift from Sabatini lab) using Xtremegene-9 (Roche). Approximately 14–16 h after transfection the growth medium was changed. Twenty-four hours after changing the medium, the lentivirus was collected and stored at −80 °C.

The resulting sgRNA–Cas9 lentivirus was used to transduce HeLa cells with 10 μg ml^−1^ polybrene and spinfection at 2,250*g* for 30 min at 37 °C. The cells were incubated for 16 h. The medium was replaced, and cells were then grown for an additional 24 h. The infected cells were selected for using 0.4 μg ml^−1^ puromycin for 48 h. The selected cells were grown for 3 days before analysis of global translation, polysome profile and rRNA and mRNA species.

For rescue experiments, GFP and GFP-C18orf21 was induced with doxycycline (1 μg ml^−1^) at the time of transduction (Extended Data Fig. 5e). Doxycycline (1 μg ml^−1^) was kept in the medium for all subsequent manipulations, to maintain robust transgene expression.

### Tissue culture

Except for HPG-incorporation experiments, all cells were cultured at 37 °C with 5% CO_2_ in Dulbecco’s modified Eagle medium supplemented with 10% tetracycline-free fetal bovine serum, 100 U ml^−1^ penicillin–streptomycin and 2 mM L-glutamine. HeLa cells were used for all experiments described here, except for lentiviral production, which was carried out in HEK293T cells. Cells were counted using a Z2 Coulter counter (Beckman Coulter).

### Cell-line generation

Cell lines with doxycycline-inducible expression of C-terminally GFP-tagged C18orf21, RMP64, RPP21 or RPP14 were created as follows. First, Gibson assembly was used to insert each construct downstream of a tetracycline-responsive promoter and upstream of Myc–TEV–GFP. These elements were flanked on both sides by ~800-base-pair homology arms for insertion into the AAVS1 safe-harbor locus^69^, which contained a puromycin-resistance marker. Each cell line was created by transiently transfecting HeLa cells with the donor plasmid, along with the pX330 plasmid, which contained a sgRNA targeting the AAVS1 locus using Lipofectamine 2000 transfection reagent (Invitrogen), according to the manufacturer’s instructions. Cells were selected with puromycin (0.4 μg ml^−1^) for 4 days.

### Competitive growth assays

To determine whether RMP24 is essential for cellular fitness, we used a competitive growth assay in which control HeLa cells (labeled with mCherry) were cocultured with cells in which RMP24 was knocked out by CRISPR–Cas9 (unlabeled cells), allowing us to use flow cytometry to monitor the relative growth of the cells grown together. Specifically, HeLa cells were spinfected (2,250*g* for 45 min at 37 °C) with lentivirus that contained Cas9 and C18orf21 or control AAVS1 sgRNA. Infected cells were selected with puromycin for 2 days. After selection, the infected cells were mixed 50:50 with mCherry-expressing HeLa cells, and the relative mCherry to uncolored cell ratio was monitored every three days using BD FACSSymphony A1 Cell Analyzer (BD Biosciences). More than 10,000 cells were analyzed per sample.

### Live-cell imaging

For live-cell imaging experiments, 50,000 cells were plated into the wells of a 12-well polymer glass-bottom plate (Cellvis). Expression of each GFP construct was induced by the addition of doxycycline (1 μg ml^−1^) for 48 h. Hoechst (0.1ug ml^−1^) was added to each well 1 h before imaging. The following protocol was used for RPP14 imaging experiments with C18orf21 knockout or a control knockout. On the first day, the HeLa cell line with the GFP-tagged RPP14 construct under doxycycline-inducible expression were plated at ~40% confluency (0.5 × 10^6^ cells) in a six-well dish. The following day, expression of RPP14–GFP was induced by the addition of doxycycline, and cells were transduced with a lentivirus that contained Cas9 and a sgRNA that targeted either *C18orf21* or *HS1* locus in the genome. Additionally, the virus contained a puromycin-resistance gene that allows for the selection of infected cells. Puromycin selection was carried out for three days; on the third day, ~50,000 surviving cells were transferred to a 12-well polymer bottom plate to allow for live-cell imaging. Images were taken ~24 h after plating. All live-cell imaging was performed with a DeltaVision Ultra High-Resolution microscope with a ×60, 1.42-NA objective (Cytiva). Images were acquired with SoftWoRx software v7.2.1. ImageJ was used to perform all image analysis and manipulation. Detailed imaging information is available in Supplementary Table 3.

### Fluorescence in situ hybridization

Cells were fixed with 4% formaldehyde in 1× PBS for 10 min and then washed three times with 1× PBS. Cells were then permeabilized by incubation with 0.5% Triton X-100 in 1× PBS for 10 min. The cells were then dehydrated by consecutively incubating them in 70% ethanol, 95% ethanol and then 100% ethanol for 5 min each. The cells were then air dried for three minutes before rehydrating them in 2× saline-sodium citrate (SSC) 50% formamide for 5 min. Cells were then prehybridized for 1 h at 37 °C in hybridization buffer (100 mg ml^−1^ dextran sulfate, 0.125 mg ml^−1^ yeast tRNA, 1 mg ml^−1^ BSA, 0.5 mg ml^−1^ salmon sperm DNA, 1 U µl^−1^ of SUPERaseIn, and 50% formamide in 2× SSC). Then, cells were incubated with 20 nM of the rRNA probe in hybridization buffer overnight at 37 °C. The following day, the cells were washed twice with 2× SSC, 50% formamide for 30 min per wash. The cells were then washed twice with 1× PBS before incubation with 1.6 μM of Hoechst in 1× PBS for 10 min. The cover slips the cells were plated on were then embedded and sealed with ProLong Gold (Thermo Fisher). For FISH, the following 3′ Cy5 labeled probes (Azenta) based on previously published sequences^70,71^ were used:

18S rRNA: 5′-GAGGTTTCCCGTGTTGAGTCAAATTAAGCCGCA-3′

5.8S rRNA: 5′-CATCGACGCACGAGCCGAGTGATCCAC-3′

28S rRNA: 5′-CCTTGTGTCGAGGGCTGACTTTCAATAG-3′

### Structural predictions

Structures were predicted using the AlphaFold 3 server^47^. Structural model figures were prepared using PyMOL (Schrodinger LLC)^72^.

### Protein purification

To express and purify C18orf21–RPP29 and C18orf21^33–220^−RPP29, the following protocol was followed. *Escherichia coli* BL21-DE3 cells were grown at 37 °C until they reached an optical density at 600 nm of 0.6–0.8. The cells were induced with 250 mM isopropyl β-d-1-thiogalactopyranoside (IPTG) for 16 h at 18 °C. Cells were pelleted at 4,000*g* in a JLA8.1000 (Beckman Coulter) in a Avanti JXN-26 centrifuge (Beckman Coulter) before being flash frozen in liquid nitrogen and stored at −80 °C. Cells were thawed in a 25 °C water bath and then subsequently resuspended in lysis buffer (50 mM Tris HCl pH 7.5, 200 mM NaCl, 5 mM β-mercaptoethanol (BME) and 1 mM phenylmethylsulfonyl fluoride (PMSF) supplemented with 1 cOmplete protease inhibitor cocktail tablet (Roche, 04693159001). After resuspension, cells were lysed by sonication (10 sec on, 30 sec off for 2.5 min). The lysate was clarified by centrifugation at 35,000*g* for 1 h at 4 °C in a JA-17 rotor (Beckman Coulter) in an Avanti JE centrifuge (Beckman Coulter). The clarified lysate was passed over a Glutathione-Agarose (GE Healthcare) column that was equilibrated with lysis buffer. The column was then washed with 20 column volumes of 50 mM Tris HCL pH 7.5, 200 mM NaCl, 1 mM DTT. For elution, the protein beads were nutated with PreScission protease overnight at 4 °C or 5 column volumes of 25 mM Tris HCl pH 8, 50 mM NaCl, 1 mM dithiothreitol (DTT), 50 mM reduced glutathione was used. Eluted protein was dialyzed into S200 buffer (25 mM Tris HCl pH 8, 100 mM NaCl, 1 mM dithiothreitol (DTT)) at 4 °C overnight. Dialyzed protein was concentrated and passed over a S200 increase column 10/300 GL (Cytiva). Data were collected with Unicorn software (Cytiva) and plotted with Prism (GraphPad). Peak fractions were analyzed by Coomassie stained SDS–PAGE gels to determine purity. Pure fractions were then pooled, concentrated, flash frozen in liquid nitrogen and stored at −80 °C.

### Immunoprecipitation–mass spectrometry

Expression of each GFP construct was induced by the addition of doxycycline for 48 h. After dox induction, cells were collected after incubation with 5 mM EDTA in 1× PBS for 5 min at 37 °C. Collected cells were pelleted by centrifugation at 200*g*. Cells were washed once with 1× PBS and then once with 1× lysis buffer (25 mM HEPES pH 8.0, 2 mM MgCl_2_, 0.1 mM EDTA pH 8.0, 0.5 mM EGTA pH 8.0, 300 mM KCl and 10% glycerol). Cells were pelleted between each wash step. Finally, the cells were resuspended in a 1:1 ratio in 1× lysis buffer and flash frozen in liquid nitrogen, before being stored at −80 °C. Polysome lysis buffer (20 mM Hepes pH 7.5, 100 mM KCl, 5 mM MgCl_2_, 1% Triton X-100) supplemented with 1% CHAPS, 1 cOmplete EDTA-free protease inhibitor cocktail (Roche) and 0.02 U μl^−1^ of SUPERaseIn (Invitrogen) was added to each frozen cell pellet before thawing the cells in a 37 °C water bath. Cells were lysed by 10 cycles of sonication at high amplitude with a 30 s on time and 1 min off time (Bioruptor, Diagenode). The lysate was clarified by centrifugation at 21,000*g* for 30 min at 4 °C. Clarified lysate was then incubated with Protein A beads (BioRad) coupled to a rabbit anti-GFP antibody (83 µg per 150 µl packed beads)^73^ at 4 °C for 2 h on a rotating wheel. Following incubation with the clarified lysate the beads were washed 4 times for 5 min with polysome lysis buffer supplemented with 1 mM DTT, 10 µg ml^−1^ leupeptin, pepstatin and chymostatin, and 0.02 U μl^−1^ SUPERaseIn. Three elutions were performed by rotating the beads with 2 bead volumes of 100 mM glycine pH 2.6 for 5 min at 4 °C. The three elutions were pooled and Tris pH 8.5 was added to a final concentration of 200 mM. After elution, one-fifth of the final volume of 100% trichloroacetic acid was added to each elution. The eluted proteins were precipitated overnight at 4 °C. The precipitated proteins were pelleted by centrifugation at 21,000*g* for 30 min. The proteins were then washed three times with 1 ml ice-cold acetone before being dried in a speedvac for 5 min at 30 °C. The proteins were then resuspended in 5% SDS, 50 mM tetraethylammonium bromide pH 8.5 (TEAB) and 20 mM DTT, and then were incubated at 95 °C for 10 min. The resuspended proteins were then allowed to cool to room temperature. Next, 40 mM iodoacetamide was added to the resuspended proteins, and the alkylating reaction was allowed to proceed for 30 min in the dark before being quenched by the addition of 2.5% vol/vol phosphoric acid. Following alkylation six times the volume of S-trap binding buffer (90% methanol, 100 mM TEAB, pH 7.55) was added and the proteins were loaded onto an S-Trap microcolumn (Protifi) by centrifugation at 4,000*g* for 1 min. The eluate was passed through the column a second time, and subsequently the column was washed four times with S-trap binding buffer. An on-column digestion was performed by adding 1 µg of trypsin diluted in 50 mM TEAB pH 8.5 to the top of the column and placing the column in a humidified chamber at 37 °C overnight. Following overnight digestion, the tryptic peptides were eluted with 40 µl of 50 mM TEAB, followed by 40 µl of 0.2% formic acid and finally with 35 µl 50% acetonitrile/0.2% formic acid. The eluted peptides were pooled, flash frozen and then lyophilized overnight. The amount of eluted peptide obtained was quantified using the Pierce fluorometric peptide assay kit (Pierce), following the manufacturer’s protocol. The lyophilized peptides were resuspended in 0.1% formic acid, to a final concentration of 250 ng µl^−1^. Two-hundred fifty nanograms of each sample was then loaded onto an Exploris 480 Orbitrap mass spectrometer, equipped with a FAIMS Pro source connected to an EASY-nLC chromatography system. The injected peptides were separated on a 25-cm analytical column (PepMap RSLC C18 3 µm, 100 A, 75 µm). Liquid chromatography was performed with a flow rate of 300 nl min^−1^. A 120-minute step gradient was performed as follows. (1) 6–21%, 41 min B; (2) 21–36% B, 20 min; (3) 36–50% B, 10 min; (4) 50–100% B, 15 min; (5) 100–2% B, 6 min; (6) 2–100%, 6 min. The orbitrap and FAIMS were operated in positive-ion mode with a positive-ion voltage of 1,800 V; with an ion-transfer tube temperature of 270 °C; using standard FAIMS resolution and compensation voltages of –45 and –65 V. Full-scan spectra were acquired in profile mode at a resolution of ×120,000, with a scan range of 350–1,200 *m/z*, automatically determined maximum fill time, standard AGC target, intensity threshold of 5 × 10^3^, 2–5 charge state and dynamic exclusion of 30 sec.

For quantitative proteomics, 1 µg of trypsin-digested peptides was resuspended in 50 mM TEAB (pH 8.5) and labeled using the TMTpro 16plex Isobaric Labeling Reagent Set (Thermo Fisher Scientific, A44521). Each sample was labeled with TMTpro 16-plex reagents at a 20:1 label-to-peptide ratio (wt/wt) for 1 h at room temperature. The labeling reaction was then halted by adding 0.2% hydroxylamine for 15 min at room temperature. After quenching, the samples were pooled on ice, flash-frozen and lyophilized. The combined TMT-labeled peptides were cleaned and fractionated using the Pierce High pH Reversed-Phase Peptide Fractionation Kit (Thermo Fisher Scientific, 84868), following the manufacturer’s protocol for TMT workflows. The fractions were subsequently flash-frozen and lyophilized. Lyophilized peptides were resuspended in 0.2% formic acid to a concentration of 250 ng µl^−1^. Mass spectrometry was performed using an Orbitrap Exploris mass spectrometer equipped with a FAIMS Pro interface connected to an Easy-nLC 1200 chromatography system (Thermo Fisher Scientific). Peptides were separated using an Aurora Ultimate 25 cm column (75 µm × 25 cm, 120 Å) from IonOpticks (Fitzroy) at 400 nl min^−1^ on a gradient of 5–25% B for 110 min, 25–40% B for 10 min, 40–95% B for 10 min, 95% B over 10 min, 95–2% B for 2 min, 2% B for 2 min, 2–98% B for 2 min, 98% B for 2 min, 98–2% B for 2 min and 2% B for 2 min, using 0.1% FA in water for A and 0.1% FA in 80% acetonitrile for B. The Orbitrap and FAIMSpro were operated in positive-ion mode with a positive-ion voltage of 1,800 V, an ion-transfer tube temperature of 270 °C, and a 4.6 L min^−1^ carrier gas flow, using standard FAIMS resolution and compensation voltages of –45 and –65 V. Full-scan spectra were acquired in profile mode at a resolution of 60,000 (MS1) and 45,000 (MS2), with a scan range of 400–1,400 *m/z*, custom maximum fill time (50 ms), custom AGC target (300% MS1, 200% MS2), isolation windows of *m/z* 0.7, intensity threshold of 5.0 × 10^4^, 2–5 charge state, dynamic exclusion of 30 s and 32% HCD collision energy.

Proteome Discoverer 2.4 (Thermo Fisher Scientific) was used to process the raw files. Sequest HT was used to identify proteins and peptides (Thermo Fisher Scientific). The human protein database (UP000005640) with the GFP sequence was used as the reference database.

For label-free mass spectrometry, tryptic peptides with up to two missed cleavages were allowed. Mass tolerances for precursors was set to 10 p.p.m., and fragments were set to 0.02 Da. The analysis included the following post-translational modifications: dynamic oxidation (+15.995 Da; methionine), dynamic acetylation (+42.011 Da; N terminus), dynamic methionine loss (–131.04 Da; methionine N terminus), dynamic methionine loss combined with acetylation (–89.03 Da; methionine N terminus), and static carbamidomethylation (+57.021 Da; cysteine). For TMT experiments, we included static TMTpro ( + 304.207 Da; any N terminus) and static TMTpro (+304.207 Da; lysine). TMTpro isotope-correction values were accounted for (Thermo Fisher; A44521 lot no. ZK391244). Percolator was used to filter the identified peptides to ensure a false discovery rate (FDR) of less than 0.01. All IP–MS data are detailed in Supplementary Table 1. The mass-spectrometry proteomics data have been deposited to the ProteomeXchange Consortium through the PRIDE^74^ partner repository with the dataset identifier PXD065571 and 10.6019/PXD065571.

### Ribonucleoprotein immunoprecipitation

Cells were collected, and immunoprecipitations were carried out as described above in the IP–MS methods section. There were two major changes to the above protocol. First, a GFP nanobody (50 µg per 5 µl packed beads)^75^ was coupled to NHS Mag Sepharose beads (Cytiva) for the immunoprecipitation and second, instead of eluting the bound proteins off with glycine, TRI reagent (Invitrogen, AM9738) was added to the beads.

### RNA extraction

Four hundred microliters of TRI Reagent (Invitrogen, AM9738) were added directly to beads or cells. RNA was then purified using Phasemaker tubes (Invitrogen, A33248), according to the manufacturer’s instructions or by phenol-chloroform extractions. The purified RNA pellet was washed with 70% ethanol and resuspended in RNase-free water. The RNA concentration was quantified by nanodrop. For analysis of RNA integrity and to monitor mature 18/28S levels, the total RNA was diluted to 5 ng μl^−1^ and run on an Agilent BioAnalyzer 2100 using the Agilent RNA 6000 Pico Reagents Kit (5067–1513).

### RT–PCR

After isolation of the bound RNA reverse transcription reactions were performed using the Maxima First Strand cDNA synthesis kit (Thermo Scientific), following the manufacturer’s protocol. Following cDNA synthesis, PCR reactions were performed with Q5 2× master mix (New England Biolabs). Primers complementary to both H1 RNA and MRP RNA were added to the reaction at a final concentration of 500 nM each. The forward and reverse H1 RNA primers had 24 bases added to their 5′ ends to help separate the H1 PCR product from the MRP PCR product. PCR products were electrophoresed on a 1.5% agarose gel. Bands were visualized by staining the gel with ethidium bromide, and the gel was imaged with a Chemidoc Imaging System (Biorad). ImageJ was used to quantify the intensity of each band and Prism (GraphPad) was used to analyze and plot the data.

### Quantitative real-time PCR

cDNA was prepared as described above. The PowerUp SYBR Green Master Mix for qPCR (A25742) was used for qPCR reactions on the QuantStudio 6 systems, according to manufacturer’s instructions. RNA levels were quantitatively assessed by standard curve interpolation. rRNA levels were normalized to GAPDH mRNA. For qPCR, the following oligonucleotides were used. ITS1 cleavage: 5′-TCCCCGTGGTGTGAAACC-3′ and 5′-GCTCCCGACGACGCAC-3′. GAPDH: 5′-TCGGAGTCAACGGATTTGGT-3′ and 5′- TTCCCGTTCTCAGCCTTGAC-3′. MALAT1 uncleaved product was normalized to total MALAT1 levels. Precursor MALAT1: 5′-GCCAAGCTAGCATCTTAGCGG-3′ and 5′-AAGCAAAGACGCCGCAG-3′. Total MALAT1: 5′-CTAAGGTCAAGAGAAGTGTCAGCC-3′ and 5′-ACCTCGACACCATCGTTACC-3′.

### RNA sequencing

For RIP-seq library preparation, libraries were prepared with the Watchmaker library prep kit (Watchmaker Genomics) with a QiaFast Select rRNA kit (Qiagen) module for rRNA depletion, and each step was performed according to the manufacturer’s instructions. For the analysis of total RNA or rRNA from knockout cells, the total RNA was directly prepped using the Kapa HyperPrep kit without any depletion or enrichment modules, according the manufacturer’s instructions. For analysis of mRNAs from knockout cells, Kapa mRNA HyperPrep kit was used to enrich for polyadenylated mRNAs, followed by library prep as described in the manufacturer’s manual. The libraries were sequenced using the Element AVITI with standard 150× 150-base-pair paired-end reads.

### Analysis of RNA sequencing

For the RIP-seq and mRNA-seq experiments, reads were mapped to the genome using STAR^76^ with the following options–runThreadN 6–runMode alignReads–outFilterMultimapNmax 1–outFilterType BySJout–outSAMattributes All–outSAMtype BAM SortedByCoordinate. For the analysis of rRNA fragments, total RNA-seq reads for knockout cells were mapped to the human 45s rRNA using STAR with the following options–runThreadN 2–limitBAMsortRAM 10000000000–runMode alignReads–outFilterMultimapNmax 5000–winAnchorMultimapNmax 1000–outFilterType BySJout–outSAMattributes All–outSAMtype BAM SortedByCoordinate. To analyze non-polyadenylated RNAs such as H1 and MRP RNA, total RNA sequencing was mapped to the human genome using STAR with the following options–runThreadN 2–outFilterMultimapNmax 1000–winAnchorMultimapNmax 2000–outFilterType BySJout–outSAMattributes All–outSAMtype BAM SortedByCoordinate

For RIP-seq and mRNA-seq experiments, reads were mapped to the human genomic sequences and annotations were downloaded from the GENCODE website (release 25, GRCh38.p7, primary assembly or main annotation). For analysis of rRNA in knockout cells, the reads were mapped to a custom genome containing the sequence of the human precursor 45S ribosomal RNA (Gene ID 100861532). Exon or rRNA-mapping reads were quantified using htseq-count^77^ (0.11.0), with the parameters -f bam -t exon -s reverse –nonunique all; a read cut-off of ≥20 reads in each sample was applied for each gene. For rRNA, a custom annotation file was generated containing the human 5′ ETS, 18S, ITS1, 5.8S, ITS2, 28S and 3′ ETS (Fig. 5f and Extended Data Fig. 6g).

Statistical analysis of immunoprecipitation fold enrichment was calculated with DESeq2 (ref. ^78^) using the interaction term to normalize IP to input RNA-seq counts. For quantification of rRNA, the raw HTseq-count reads for each rRNA region were normalized to the total reads mapping to rRNA then compared to the control. To quantify reads at junctions of each domain of precursor rRNAs, we counted all reads that contained at least 20 nucleotides flanking each junction. We counted reads containing the following sequences for each junction, where ‘^’ indicates the junction, and normalized the junction reads to the number of million rRNA mapping reads (Extended Data Fig. 7a). All analyzed RNA-seq data can be found in Supplementary Table 2.

5′ ETS–18S: 5′-ACCTGCGGAAGGATCATTAAC^GGAGCCCGGAGGGCGAGGC-3′

18S–ITS1: 5′-CCGCGCCGCCGGGCACGGCC^CCGCTCGCTCTCCCCGGCCT-3′

ITS1–5.8S: 5′-CTCGCCAAATCGACCTCGTA^CGACTCTTAGCGGTGGATCA-3′

5.8S–ITS2: 5′-CGCCTGTCTGAGCGTCGCTT^GCCGATCAATCGCCCCCGGG-3′

ITS2–28S: 5′-CGGCCCGTCCCCCTCCGAGA^CGCGACCTCAGATCAGACGT-3′

28S–3′ ETS: 5′-CCTCGACACAAGGGTTTGTC^CGCGCGCGCGCGCGCGCGCG-3′

### Analysis of mature rRNA

Bioanalyzer was performed using 5300 Fragment Analyzer; 18S and 28S rRNA was also visualized by running 200 ng total RNA on an agarose gel with SYBR Gold. The quantification of the 18S to 28S ratio was performed in ImageJ.

### Homopropargylglycine incorporation assays

Before HPG incorporation, the medium was replaced with methionine-free medium for 30 min. Cells were labeled with 500 μM L-homopropargylglycine in methionine-free medium and incubated at 37 °C for 30 min. After amino acid incorporation, cells were washed twice with PBS, trypsinized and fixed with 4% formaldehyde in PBS for 15 min at room temperature. Fixed cells were washed once with PBS, then blocked using AbDil (20 mM Tris-HCl, 150 mM NaCl, 0.1% Triton X-100, 3% bovine serum albumin, 0.1% NaN3, pH 7.5) for 30 min at room temperature. Click chemistry was performed in solution by incubating the fixed cells in 120 μl of 100 mM Tris pH 8.0, 1 mM CuSO_4_, 5 μM AlexaFluor Azide and 0.1 M ascorbic acid at room temperature for 30 min in the dark. Cells were washed three times with PBS containing 0.1% Triton X-100, strained and analyzed by flow cytometry using the BD FACSSymphony A1 Cell Analyzer (BD Biosciences) and FlowJo. Dead cells and cell doublets were removed from the analysis on the basis of forward and side scatter. More than 10,000 cells were analyzed per replicate.

### Polysome gradient analysis

A 15-cm plate of cells at ~80% confluency was treated with 100 μg ml^−1^ cycloheximide for 2 min at 37 °C, washed with PBS supplemented with 100 μg ml^−1^ cycloheximide, and then trypsinized with 100 μg ml^−1^ cycloheximide at 37 °C. Cells were spun down at 500*g* for 3 min at 4 °C, washed twice with cold PBS supplemented with 100 μg ml^−1^ cycloheximide. Cells were lysed in polysome lysis buffer (20 mM Tris pH 7.4, 100 mM KCl, 5 mM MgCl_2_, 1% (vol/vol) Triton X-100, 100 μg ml^−1^ cycloheximide, 500 U ml^−1^ RNaseIn Plus, 1× cOmplete protease inhibitor cocktail) and incubated on ice for 10 min. The lysate was passed through a 26-gauge syringe to ensure efficient cell lysis. The lysate was spun at 1,300*g* for 10 min at 4 °C and the supernatant was flash frozen with LN2 and stored at −80 °C. The lysate was loaded onto a 10–50% sucrose gradient with cycloheximide and SuperaseIN and centrifuged at 36,000*g* for 2 h at 4 °C. Fractionation and measurement of absorbance at 254 nm was performed using the BioComp gradient fractionator.

### Western blot analysis

HeLa cells expressing dox-inducible GFP or GFP–C18orf21 were infected with control or C18orf21 gRNA1 as described above. Three days after knockout, a confluent six-well dish worth of cells was washed once with PBS and lysed in 1× Laemmli sample buffer (100 mM Tris pH 6.8, 12.5% glycerol, 1% SDS, 0.1% bromophenol blue, 200 mM β-mercaptoethanol) and heated at 95 °C for 5 min. Whole-cell lysates were then sonicated for 5 s at 10% amplitude using a Branson Digital Sonifier 450 to fragment genomic DNA before further boiling. Proteins were separated by SDS–PAGE and transferred onto PVDF membranes (VWR). Membranes were rinsed briefly with TBST and blocked for 1 h in 5% milk at room temperature. Primary antibodies were diluted in 5% milk and incubated with the membranes overnight at 4 °C, followed by four 5-minute washes with TBST. Secondary antibodies, diluted in 5% milk, were applied for 1 h at room temperature, followed by four additional washes with TBST and two rinses with PBS. The blots were imaged using an Odyssey Clx machine (LI-COR).

The following primary antibodies were used: anti-C18orf21 (1:1,000, Proteintech, 24977-1-AP) and anti-GAPDH (1:4,000, Santa Cruz Biotechnology, sc-47724). The following secondary antibodies were used: IRDye 680RD goat anti-rabbit (LI-COR 92668071), IRDye 680RD goat anti-mouse (LI-COR 92668070), IRDye 800CW goat anti-rabbit (LI-COR 92632211), IRDye 800CW goat anti-mouse (LI-COR 92632210). All secondary antibodies were diluted 1:10,000 in 5% milk and TBST.

### Immunofluorescence

Cells were seeded on poly-L-lysine coated coverslips and fixed in PBS with 4% formaldehyde and 0.25% Triton X-100 at room temperature for 15 min. Following fixation, cells were washed three times with PBS and 0.1% Triton X-100 and blocked in AbDil for 0.5–1 h. After blocking, cells were stained at 4 °C in a humidified chamber overnight with anti-C18orf21 antibody (1:200, Proteintech, 24977-1-AP), anti-coilin (1:500 Proteintech, 10967-1-AP) and anti-GFP (1:1000, Proteintech, gb2AF488) diluted in AbDil. Cells were washed with PBS and 0.1% Triton X-100 three times, then incubated in secondary antibody (donkey anti-rabbit-IgG (H+L) conjugated to Alexa Fluor 647) diluted in AbDil for 1 h at room temperature. Cells were stained in Hoescht for 15 min at room temperature, then washed 3 times with PBS and 0.1% Triton X-100 before mounting in PPDM (0.5% p-phenylenediamine and 20 mM Tris-Cl, pH 8.8, in 90% glycerol) and sealed with nail polish. The DeltaVision Ultra High-Resolution microscope with a ×60, 1.42 NA objective (Cytiva) was used to image the cells and ImageJ was used to perform all image analysis. The presented images were deconvolved and maximum projected.

### Propidium and annexin V staining for death and apoptosis

Knockout cells were grown in six-well plates and at the indicated days, the medium (2 ml) was directly strained into flow cytometry tubes. The cells subsequently were trypsinized without EDTA and strained into flow-cytometry tubes. Four drops of Annexin V ready flow conjugate (R37177) and 2.5 mM calcium chloride were added to the cells then incubated at room temperature for 15 min. Propidium iodide (1 μg ml^−1^) was added 10 min before flow-cytometry analysis. More than 10,000 cells were analyzed per replicate.

### CRISPR knockout efficiency analysis

HeLa cells were infected with control gRNA or C18orf21 gRNA1, as described above. Three days after knockout genomic DNA was collected, as described above, PCR of the isolated genomic DNA was carried out with the following primers: C18orf21 FWD: 5′-GACTGGACGCGAAGGAAAC-3′ C18orf21 Rev: 5′-GGGGAGATTAGGAGGAACCAGC-3′. PCR products were electrophoresed on a 1.5% agarose gel and purified by gel extraction (Qiagen). Sanger sequencing was performed with the C18orf21 FWD primer. The resulting traces were analyzed by ICE (Synthego) to determine cleavage efficiency and the distribution of indels introduced into the genomic locus^79^.

### Reporting summary

Further information on research design is available in the Nature Portfolio Reporting Summary linked to this article.

## Online content

Any methods, additional references, Nature Portfolio reporting summaries, source data, extended data, supplementary information, acknowledgements, peer review information; details of author contributions and competing interests; and statements of data and code availability are available at 10.1038/s41594-025-01690-7.

## Supplementary information

Supplementary Information FACS gating strategy.

Reporting Summary

Peer Review File

Supplementary Table 1 Mass spectrometry data.

Supplementary Table 2 DESeq2 results for RNA sequencing analysis.

Supplementary Table 3 Light microscopy reporting table.

## Source data

Source Data Statistical source data.

Source Data Unprocessed gels.

## Data Availability

RNA sequencing data and associated analyses were deposited in the Gene Expression Omnibus (GSE279079). The mass-spectrometry proteomics data have been deposited to the ProteomeXchange Consortium via the PRIDE^74^ partner repository with the dataset identifier PXD065571 and 10.6019/PXD065571. Source data are provided with this paper.
