# Supplementary material for: RNase MRP subunit composition and role in 40S ribosome biogenesis
Source: Nat Struct Mol Biol. 2025 Oct 24;33(1):20–33. doi: 10.1038/s41594-025-01690-7 (PMC12819141; doi:10.1038/s41594-025-01690-7)

nature structural & molecular biology

Supplementary information https://doi.org/10.1038/s41594-025-01690-7

RNase MRP subunit composition and role in 40S ribosome biogenesis

In the format provided by the authors and unedited

HPG staining gating strategy

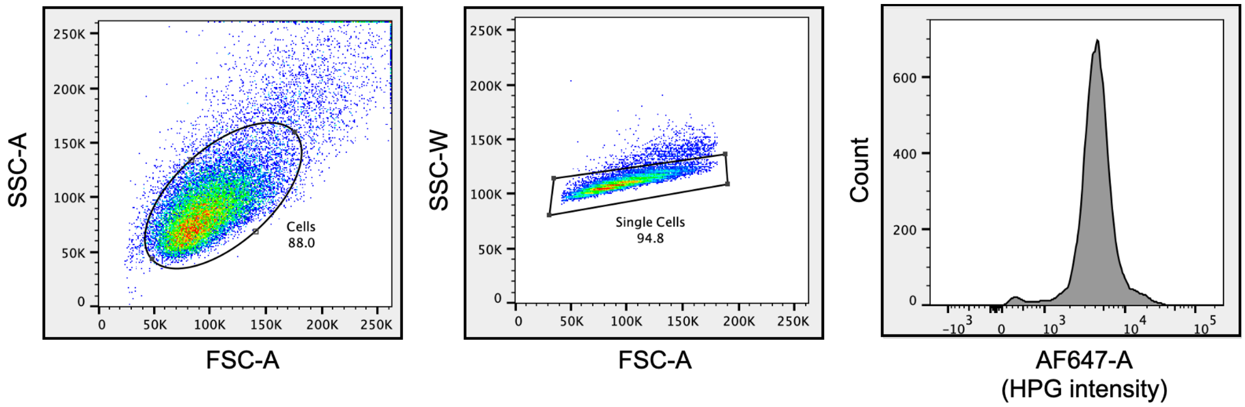

Apoptosis and live/dead gating strategy

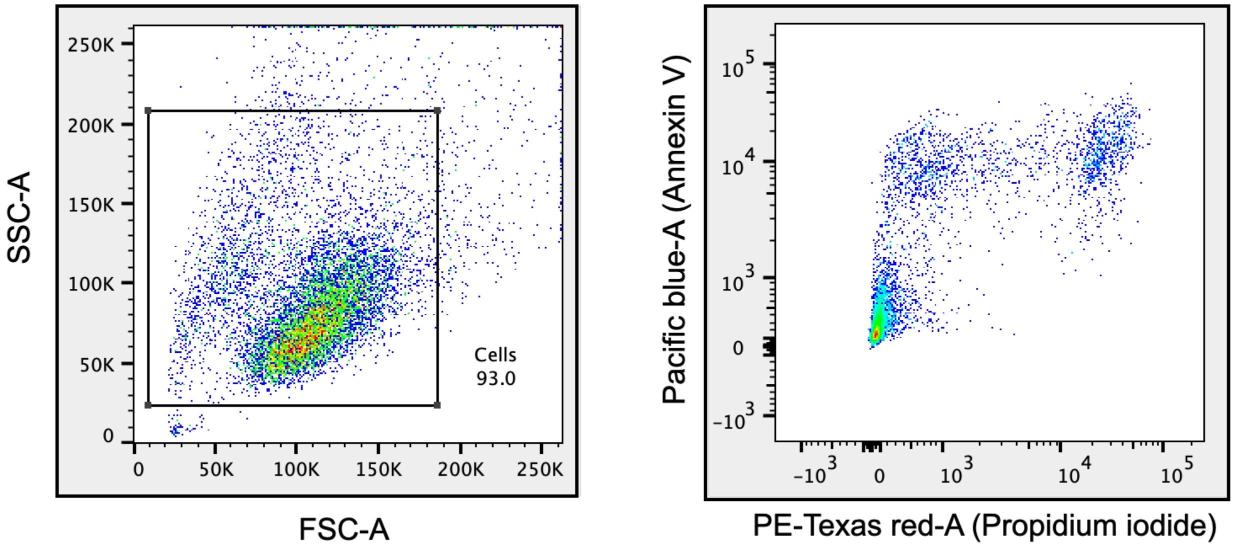

Supplement: Supplementary file 1 — FACS gating strategy. [file 41594_2025_1690_MOESM1_ESM.pdf]
