# Supplementary material for: RNase MRP subunit composition and role in 40S ribosome biogenesis
Source: Nat Struct Mol Biol. 2025 Oct 24;33(1):20–33. doi: 10.1038/s41594-025-01690-7 (PMC12819141; doi:10.1038/s41594-025-01690-7)
Supplement: Supplementary file 2 — Reporting Summary [file 41594_2025_1690_MOESM2_ESM.pdf]

nature portfolio

Corresponding author(s): Iain Cheeseman

Last updated by author(s): Aug 13, 2025

Reporting Summary

Nature Portfolio wishes to improve the reproducibility of the work that we publish. This form provides structure for consistency and transparency in reporting. For further information on Nature Portfolio policies, see our Editorial Policies and the Editorial Policy Checklist.

Statistics

For all statistical analyses, confirm that the following items are present in the figure legend, table legend, main text, or Methods section.

n/a	Confirmed	
☐	☒	The exact sample size (*n*) for each experimental group/condition, given as a discrete number and unit of measurement
☐	☒	A statement on whether measurements were taken from distinct samples or whether the same sample was measured repeatedly
☐	☒	The statistical test(s) used AND whether they are one- or two-sided<br>*Only common tests should be described solely by name; describe more complex techniques in the Methods section.*
☒	☐	A description of all covariates tested
☒	☐	A description of any assumptions or corrections, such as tests of normality and adjustment for multiple comparisons
☐	☒	A full description of the statistical parameters including central tendency (e.g. means) or other basic estimates (e.g. regression coefficient) AND variation (e.g. standard deviation) or associated estimates of uncertainty (e.g. confidence intervals)
☐	☒	For null hypothesis testing, the test statistic (e.g. *F*, *t*, *r*) with confidence intervals, effect sizes, degrees of freedom and *P* value noted<br>*Give P values as exact values whenever suitable.*
☒	☐	For Bayesian analysis, information on the choice of priors and Markov chain Monte Carlo settings
☒	☐	For hierarchical and complex designs, identification of the appropriate level for tests and full reporting of outcomes
☒	☐	Estimates of effect sizes (e.g. Cohen's *d*, Pearson's *r*), indicating how they were calculated

Our web collection on statistics for biologists contains articles on many of the points above.

Software and code

Policy information about availability of computer code

Data collection	Novaseq SP was used for sequencing; Orbitrap exploris or Orbitrap eclipse was used for mass spectrometry; Deltavision software was used for microscopy; BD FACSdiva was used to collect flow cytometry; LI-COR Odyssey CLx was used to image western blots.
Data analysis	Cutadapt (v3.7), STAR (v2.7.1), htseq-count (v0.11.0), DEseq2 (v1.38.3), ggplot2 (v3.4.4) were used for analysis of RNA-seq data.<br>Proteome Discoverer (v2.4) was used for analysis of mass spec data.<br>Softworx software v7.2.1 was used for image acquisition and deconvolution<br>Fiji/ImageJ (v2.16.0) was used for analysis of images.<br>FlowJo v10.7.1 was used for analysis of flow cytometry data.<br>Unicorn (v7.5) was used to collect gel filtration data.<br>Prism (v10.2.2) was used to plot chromatograms acquired from protein purification by gel filtration.<br>AlphaFold3 (online server) was used for structure predictions.<br>Pymol (v2.4.1) for preparing structural model figures.

For manuscripts utilizing custom algorithms or software that are central to the research but not yet described in published literature, software must be made available to editors and reviewers. We strongly encourage code deposition in a community repository (e.g. GitHub). See the Nature Portfolio guidelines for submitting code & software for further information.

Data

Policy information about availability of data

All manuscripts must include a data availability statement. This statement should provide the following information, where applicable:

- Accession codes, unique identifiers, or web links for publicly available datasets
- A description of any restrictions on data availability
- For clinical datasets or third party data, please ensure that the statement adheres to our policy

All data necessary for the conclusions of the paper are provided with the article. Sequencing data and associated analyses were deposited in Gene Expression Omnibus (GSE279079). Mass spectrometry data were deposited in the PRIDE database (PXD065571).

Research involving human participants, their data, or biological material

Policy information about studies with human participants or human data. See also policy information about sex, gender (identity/presentation), and sexual orientation and race, ethnicity and racism.

Reporting on sex and gender	N/A
Reporting on race, ethnicity, or other socially relevant groupings	N/A
Population characteristics	N/A
Recruitment	N/A
Ethics oversight	N/A

Note that full information on the approval of the study protocol must also be provided in the manuscript.

Field-specific reporting

Please select the one below that is the best fit for your research. If you are not sure, read the appropriate sections before making your selection.

☒ Life sciences ☐ Behavioural & social sciences ☐ Ecological, evolutionary & environmental sciences

For a reference copy of the document with all sections, see nature.com/documents/nr-reporting-summary-flat.pdf

Life sciences study design

All studies must disclose on these points even when the disclosure is negative.

Sample size	No sample size calculation was performed. Sample sizes were chosen according to field standards and are sufficient based on the relatively large effect size between groups.
Data exclusions	No data was excluded
Replication	All experiments were replicated at least twice with the similar results. Representative plots are shown when appropriate.
Randomization	Experimental groups were assessed in the same experiment as controls to eliminate covariates.
Blinding	All cell lines and plasmids were given non-descriptive sequential numbers and decoded after data acquisition/analysis.

Reporting for specific materials, systems and methods

We require information from authors about some types of materials, experimental systems and methods used in many studies. Here, indicate whether each material, system or method listed is relevant to your study. If you are not sure if a list item applies to your research, read the appropriate section before selecting a response.

Materials & experimental systems

n/a	Involved in the study
	☒ Antibodies
	☒ Eukaryotic cell lines
☒	Palaeontology and archaeology
☒	Animals and other organisms
☒	Clinical data
☒	Dual use research of concern
☒	Plants

Methods

n/a	Involved in the study
☒	ChIP-seq
	☒ Flow cytometry
☒	MRI-based neuroimaging

Antibodies

Antibodies used

Anti-GFP (Goat, Cheeseman lab) (83 µg/ 150 µl packed beads)
GFP nanobody (PMID: 29809153) (50 µg/5 µl packed beads)
anti-C18orf21 antibody (Proteintech, 24977-1-AP) Immunofluorescence dilution 1:200 Western blot dilution 1:1000
anti-coilin (Proteintech, 10967-1-AP) Immunofluorescence dilution 1:500
anti-GFP (Proteintech, gb2AF488) Immunofluorescence dilution 1:1000
anti-GAPDH (Santa Cruz Biotechnology, sc-47724) Western blot dilution 1:4000
IRDye 680RD Goat anti-Rabbit (LI-COR 92668071) dilution 1:10,000
IRDye 680RD Goat anti-Mouse (LI-COR 92668070) dilution 1:10,000
IRDye 800CW Goat anti-Rabbit (LI-COR 92632211) dilution 1:10,000
IRDye 800CW Goat anti-Mouse (LI-COR 92632210) dilution 1:10,000

Validation

The polyclonal Goat GFP antibody used for IP-MS efficiently pulls down GFP, validating that the antibody works (figures 1,6, and 7).
Each commercially available antibody used in this study was validated by the manufacturer as reported on their websites.
anti-C18orf21 antibody (Proteintech, 24977-1-AP): https://www.ptglab.com/products/C18orf21-Antibody-24977-1-AP.htm?srsltid=AfmBOorkYFB0vU4Obt71dnFLruSzUrbM387L1C_8aFEVwuCzD0YBiQGT
anti-coilin (Proteintech, 10967-1-AP): https://www.ptglab.com/products/COIL-Antibody-10967-1-AP.htm?srsltid=AfmBOoq22OMeiFUwIdEOWKrg_jRyre81-hR-5fTdkKYA5nRM-ctbOYYB
anti-GFP (Proteintech, gb2AF488): https://www.ptglab.com/products/GFP-Booster-Alexa-Fluor-488-gb2AF488.htm?srsltid=AfmBOop34j-xW8tInEdRADQIhEOCTY7rGrE5ZR2qCvlC6P8OpO_zebp0
anti-GAPDH (Santa Cruz Biotechnology, sc-47724): https://www.scbt.com/p/gapdh-antibody-0411?srsltid=AfmBOopp6a9BaFiM1PRd_1lvRHN_1y_ubrCE4PO8zj7dlodhjPf40IFi

Eukaryotic cell lines

Policy information about cell lines and Sex and Gender in Research

Cell line source(s)

HeLa and HEK293T (Cheeseman lab stocks) cells were used in this study. Generation of specific HeLa derived cell lines was performed as described in methods.

Authentication

Cell lines were not authenticated.

Mycoplasma contamination

Cell lines tested negative for mycoplasma contamination.

Commonly misidentified lines
(See ICLAC register)

No commonly misidentified cell lines were used in this study.

Plants

Seed stocks

N/A

Novel plant genotypes

N/A

Authentication

N/A

Flow Cytometry

Plots

Confirm that:

☒ The axis labels state the marker and fluorochrome used (e.g. CD4-FITC).

☒ The axis scales are clearly visible. Include numbers along axes only for bottom left plot of group (a 'group' is an analysis of identical markers).

☒ All plots are contour plots with outliers or pseudocolor plots.

☒ A numerical value for number of cells or percentage (with statistics) is provided.

Methodology

Sample preparation	Samples were prepared as described in the methods. Following staining cells were strained and monitored using the BD FACSymphony A1 Cell Analyzer (BD Biosciences)
Instrument	FACSymphony A1 Cell Analyzer (BD Biosciences)
Software	BD FACSDiva was used to collect data and FlowJo 9 and 10 were used to analyze the data.
Cell population abundance	N/A
Gating strategy	We used forward scatter and side scatter area to gate singlet live cells prior to analyzing HPG intensity. For apoptosis and propidium iodide experiments, cell debris was gated out prior to analyzing proportion of live/dead cells.

☒ Tick this box to confirm that a figure exemplifying the gating strategy is provided in the Supplementary Information.
