# Supplementary material for: RNase MRP subunit composition and role in 40S ribosome biogenesis
Source: Nat Struct Mol Biol. 2025 Oct 24;33(1):20–33. doi: 10.1038/s41594-025-01690-7 (PMC12819141; doi:10.1038/s41594-025-01690-7)

Figure 2G

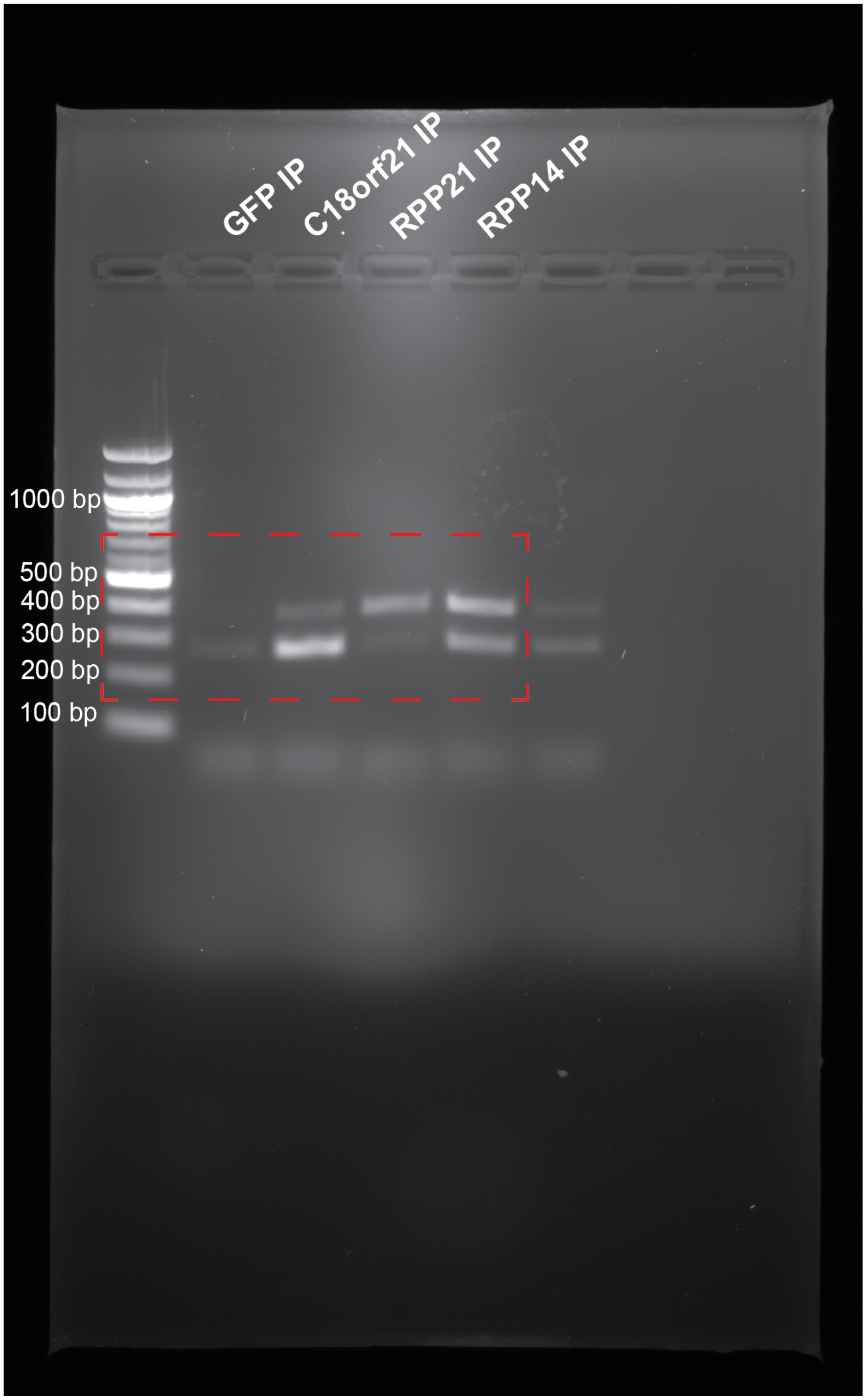
GFP IP
C18orf21 IP
RPP21 IP
RPP14 IP
1000 bp
500 bp
400 bp
300 bp
200 bp
100 bp

Figure 3C

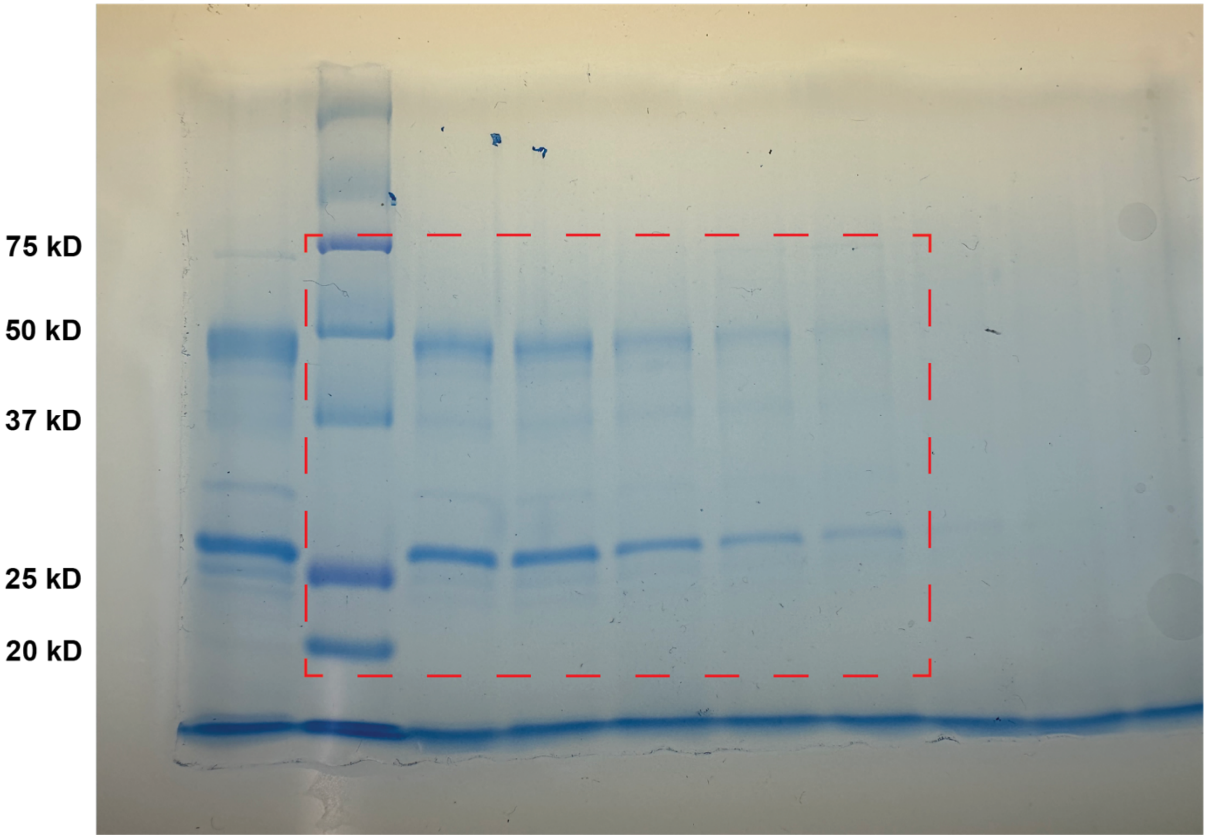

Figure 4C

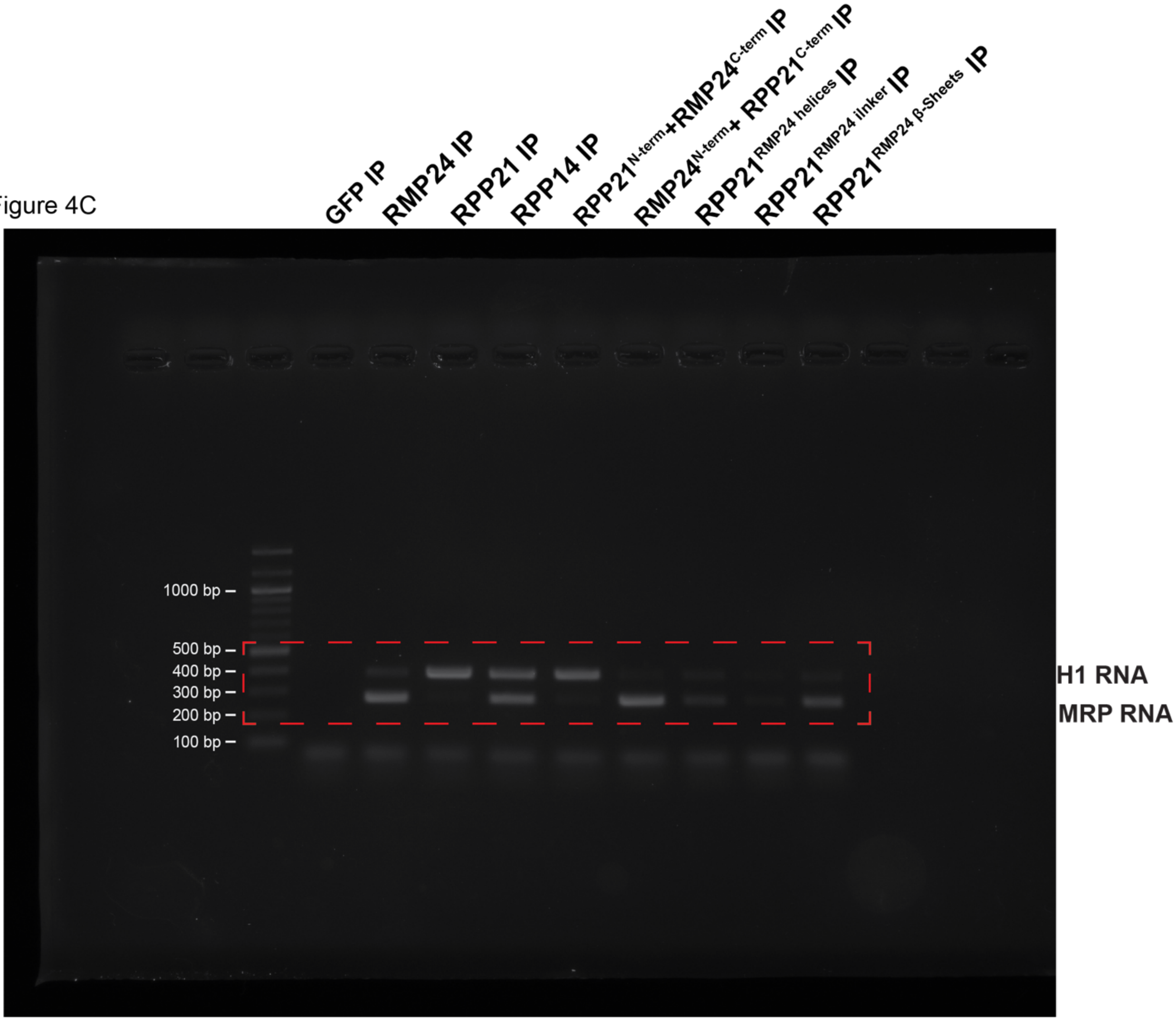
GFP IP
RMP24 IP
RPP21 IP
RPP14 IP
RPP21$^{N\text{-term}}$+RMP24$^{C\text{-term}}$ IP
RMP24$^{N\text{-term}}$+ RPP21$^{C\text{-term}}$ IP
RPP21$^{RMP24\ helices}$ IP
RPP21$^{RMP24\ linker}$ IP
RPP21$^{RMP24\ \beta\text{-Sheets}}$ IP
1000 bp
500 bp
400 bp
300 bp
200 bp
100 bp
H1 RNA
MRP RNA

Figure 6D

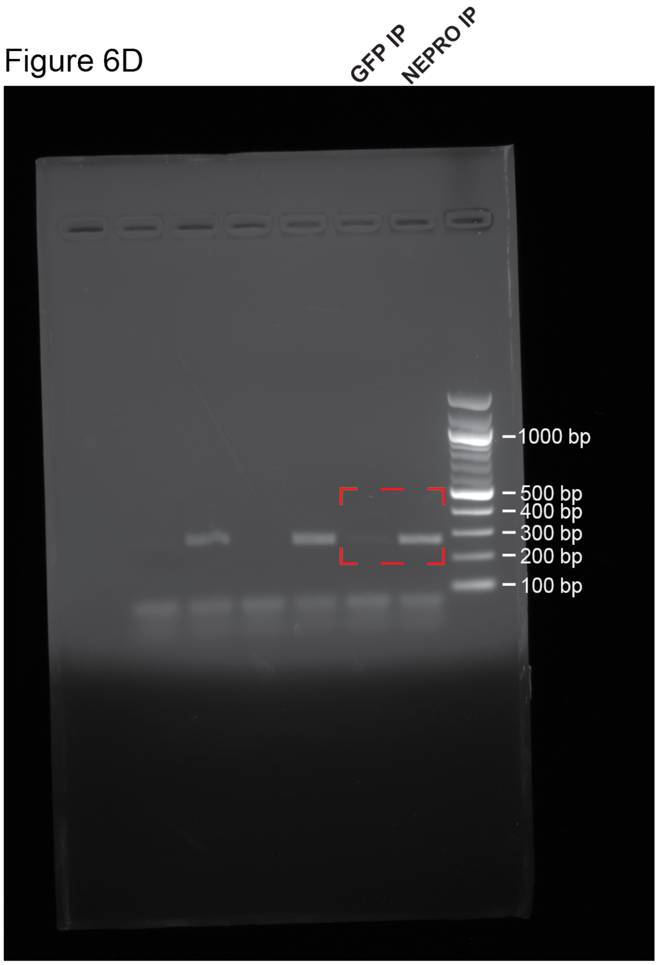

Extended Data Figure 3E

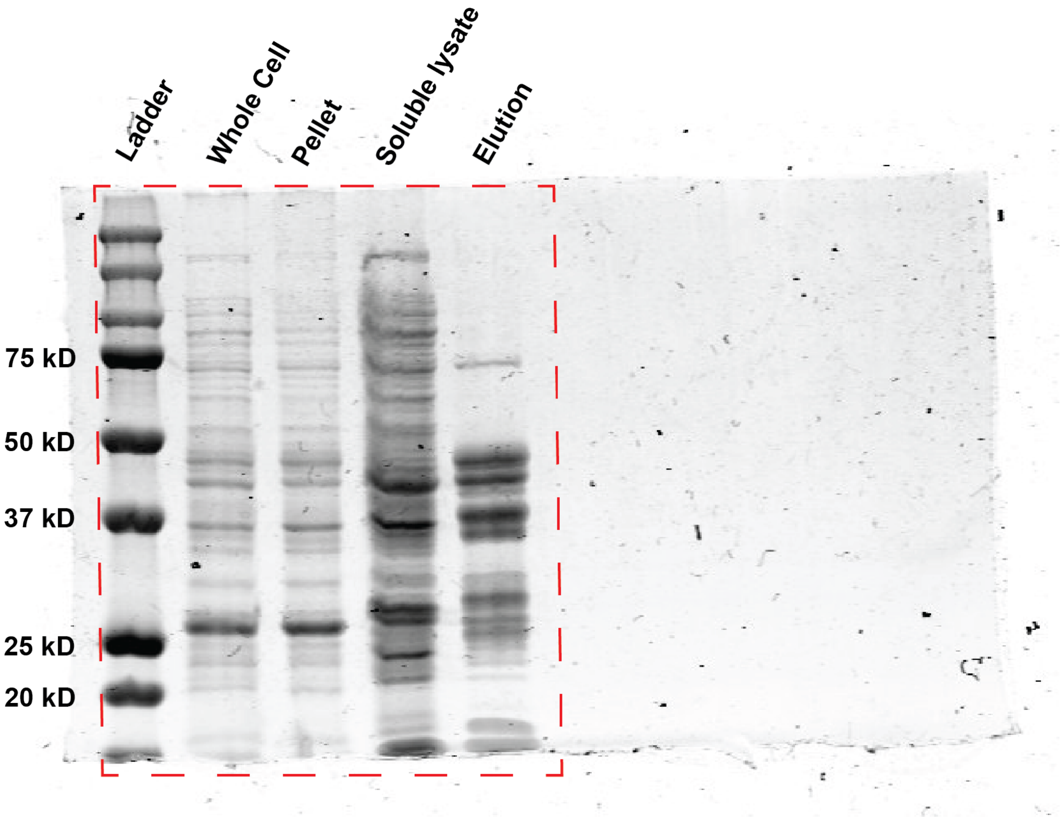

Extended data Figure 5A

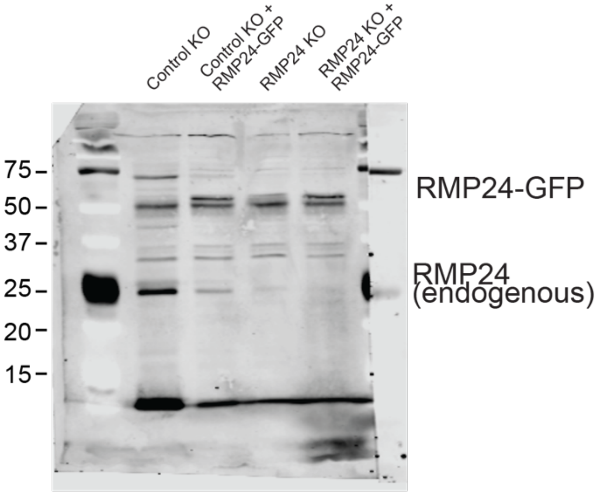

α-C18orf21

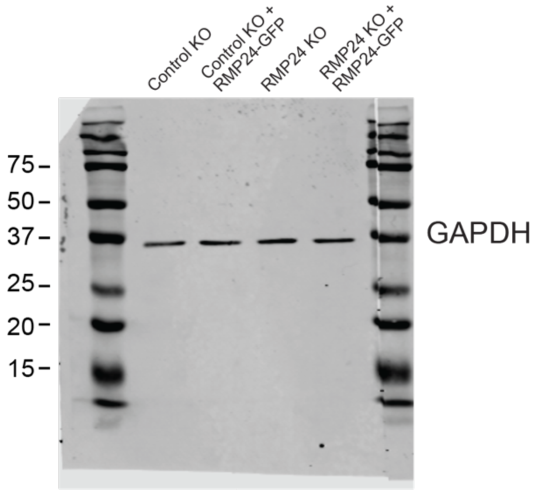

α-GAPDH

Extended data Figure 6C

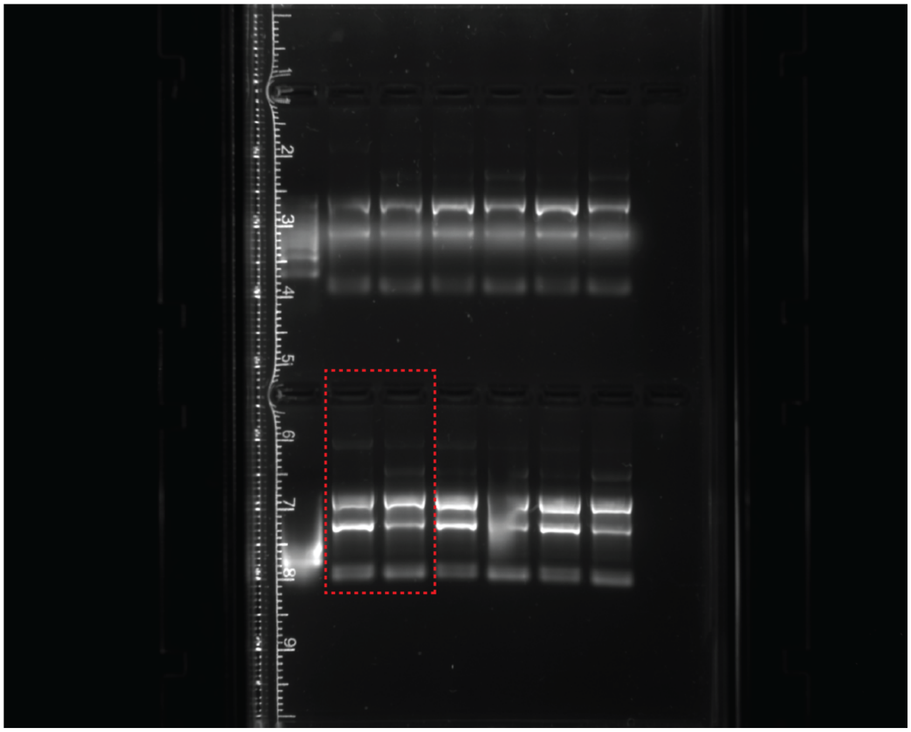

Supplement: Supplementary file 8 — Unprocessed gels. [file 41594_2025_1690_MOESM8_ESM.pdf]
