# Supplementary material for: RNase MRP subunit composition and role in 40S ribosome biogenesis
Source: Nat Struct Mol Biol. 2025 Oct 24;33(1):20–33. doi: 10.1038/s41594-025-01690-7 (PMC12819141; doi:10.1038/s41594-025-01690-7)
Supplement: Supplementary file 3 — Peer Review File [file 41594_2025_1690_MOESM3_ESM.pdf]

nature portfolio

Peer Review File

RNase MRP subunit composition and role in 40S ribosome biogenesis

Corresponding Author: Dr Iain M. Cheeseman

Parts of this Peer Review File have been redacted as indicated to remove third-party material.

Version 0:

Decision Letter:

28th Mar 2025

Dear Dr Cheeseman,

Thank you again for submitting your manuscript "Molecular determinants of RNase MRP specificity and function". We now have comments (below) from the 3 reviewers who evaluated your paper. In light of those reports, we remain interested in your study and would like to see your response to the comments of the referees, in the form of a revised manuscript.

You will see that while the reviewers appreciate the work, reviewer #1 requests acknowledgeing previous reports of RNase MRP interacting proteins, validation of the expression levels of C18ORF21 in the cell lines used, and better substantiation of conclusions about the role of RNase MRP in 40S vs 60S ribosome biogenesis, and reviewer #2 requests a more robust substantiation of the interactions between RMRPP1 and H1 or MRP RNAs, a specificity control in the RMRPP1 interaction with Rpp29 as well as other streamlining and missing controls. Editorially, we agree that strengthening the conclusions about the role of RNase MRP in 40S vs 60S ribosome biogenesis will be important for taking the manuscript forward. Please be sure to address/respond to all concerns of the referees in full in a point-by-point response and highlight all changes in the revised manuscript text file.

We are committed to providing a fair and constructive peer-review process. Do not hesitate to contact us if there are specific requests from the reviewers that you believe are technically impossible or unlikely to yield a meaningful outcome.

We appreciate the requested revisions are extensive. We thus expect to see your revised manuscript within 4-6 months. If you cannot send it within this time, please let us know. We will be happy to consider your revision as long as nothing similar has been accepted for publication at NSMB or published elsewhere. Should your manuscript be substantially delayed without notifying us in advance and your article is eventually published, the received date would be that of the revised, not the original, version.

As you already know, we put great emphasis on ensuring that the methods and statistics reported in our papers are correct and accurate. As such, if there are any changes that should be reported, please submit an updated version of the Reporting Summary along with your revision.

Reporting Summary:
https://www.nature.com/documents/nr-reporting-summary.pdf

Please note that the form is a dynamic ‘smart pdf’ and must therefore be downloaded and completed in Adobe Reader.

When submitting the revised version of your manuscript, please pay close attention to our href="https://www.nature.com/nature-portfolio/editorial-policies/image-integrity">Digital Image Integrity Guidelines. and to the following points below:

-- that unprocessed scans are clearly labelled and match the gels and western blots presented in figures.
-- that control panels for gels and western blots are appropriately described as loading on sample processing controls
-- all images in the paper are checked for duplication of panels and for splicing of gel lanes.

EXTENDED DATA FIGURES

When re-submitting your manuscript, please ensure that any supplementary figures and tables that are crucial to the manuscript's conclusions are converted into Extended Data figures and tables to increase visibility of these data. Extended Data figures and tables are online-only (present in the online PDF and full-text HTML versions of the paper), peer-reviewed display items that provide essential background to the article but are not included in the main article due to space constraints. A maximum of ten Extended Data display items (figures and tables) is permitted.

Finally, please ensure that you retain unprocessed data and metadata files after publication, ideally archiving data in perpetuity, as these may be requested during the peer review and production process or after publication if any issues arise.

Please note that all key data shown in the main figures as cropped gels or blots should be presented in uncropped form, with molecular weight markers. These data can be aggregated into a single supplementary figure. While these data can be displayed in a relatively informal style, they must refer back to the relevant figures. These data should be submitted with the last revision, prior to acceptance, but you may want to start putting it together at this point.

SOURCE DATA: we request that authors provide, in tabular form, the data underlying the graphical representations used in figures. This is to further increase transparency in data reporting, as detailed in this editorial (http://www.nature.com/nsmb/journal/v22/n10/full/nsmb.3110.html). Spreadsheets can be submitted in excel format. Only one (1) file per figure is permitted; thus, for multi-paneled figures, the source data for each panel should be clearly labeled in the Excel file; alternately the data can be provided as multiple, clearly labeled sheets in an Excel file. When submitting files, the title field should indicate which figure the source data pertains to. We encourage our authors to provide source data at the revision stage, so that they are part of the peer-review process.

While we encourage the use of color in preparing figures, please note that this will incur a charge to partially defray the cost of printing. Information about color charges can be found at http://www.nature.com/nsmb/authors/submit/index.html#costs

We require deposition of coordinates (and, in the case of crystal structures, structure factors) into the Protein Data Bank with the designation of immediate release upon publication (HPUB). Electron microscopy-derived density maps and coordinate data must be deposited in EMDB and released upon publication. Deposition and immediate release of NMR chemical shift assignments are highly encouraged. Deposition of deep sequencing and microarray data is mandatory, and the datasets must be released prior to or upon publication. To avoid delays in publication, dataset accession numbers must be supplied with the final accepted manuscript and appropriate release dates must be indicated at the galley proof stage. Please find the complete NRG policies on data availability at http://www.nature.com/authors/policies/availability.html.

Nature Structural & Molecular Biology is committed to improving transparency in authorship. As part of our efforts in this direction, we are now requesting that all authors identified as 'corresponding author' on published papers create and link their Open Researcher and Contributor Identifier (ORCID) with their account on the Manuscript Tracking System (MTS), prior to acceptance. This applies to primary research papers only. ORCID helps the scientific community achieve unambiguous attribution of all scholarly contributions. You can create and link your ORCID from the home page of the MTS by clicking on 'Modify my Springer Nature account'. For more information please visit please visit www.springernature.com/orcid.

Please use the link below to submit your revised manuscript and related files:

Link Redacted

Note: This URL links to your confidential home page and associated information about manuscripts you may have submitted, or that you are reviewing for us. If you wish to forward this email to co-authors, please delete the link to your homepage.

We look forward to seeing the revised manuscript and thank you for the opportunity to review your work.

Sincerely,
Sara

Sara Osman, Ph.D.
Senior Editor
Nature Structural & Molecular Biology

Reviewers' Comments:

Reviewer #1 (Remarks to the Author):

The authors identify and characterize two proteins that are associated with RNase MRP in preference to RNase P; C18ORF21 / RMRPP1 and C3ORF17 / Nepro. For RMRPP1 and RPP21, chimeric proteins are used to identify protein domains involved in discriminating between MRP and P association. Depletion of RMRPP1 or Nepro is reported to impair

ribosomal subunit association, with a greater reduction in 40S abundance. A disease linked mutation in Nepro is shown to interfere with MRP association.

Overall, the MS reports a lot of data that will be of wide interest. However, there are some points that should be considered.

Major points:

1: Both C18ORF21 and C3ORF17 were previously identified as RNase MRP interacting proteins based on pulldown of the RMRP RNA. See Figures 1B and 1C in:
https://www.nature.com/articles/s41586-018-0453-z.
MRP interactions were also predicted from bioinformatics:
https://bmcbioinformatics.biomedcentral.com/articles/10.1186/s12859-023-05425-7
These findings were apparently not pursued, and this does not substantially detract from novelty of the current report. They should, however, be acknowledged.

2: Knockout cells models: it might have been anticipated that C18ORF21knockout cells would not be viable. The assays were reportedly done on a puromycin selected pool of cells. Could validation data be shown to give an indication of % expression of C18ORF21 mRNA and protein in these pools?

For the growth competition assay, it would be helpful to have data to understand if the RMPP1 "KO" cells are dying (as reported for knockout of RMRP eg in Goldfarb and Cech. 2017) or alive, but growing more slowly (as seen in patient mutations in RMRP).

3: The conclusion in abstract (line 19) and elsewhere in text that "...we reveal that RNase MRP is required for 40S, but not 60S, ribosome biogenesis, uncovering an alternative pathway for ribosome assembly" is stronger than the presented data can support.

The data support some reduction in 40S:60S ratios the KO cell lines, but no data is shown on what this pathway would. Moroeover, the data presented show ratios of 60S to 40S, making it unclear that 60S production is unaffected, only that 40S is preferentially affected. These difficulties in interpretation are compounded by the uncertainty whether the defects are being studies in viable or dying cells.

This conclusion would also contrast with a previous report from cells with disease-associated mutations in the RMRP RNA where 18S and 28S rRNA were approximately equally reduced (Robertson et al, 2022). Also of note are previous findings that RMRP knockout in mammalian cells causes the appearance of undefined rRNA processing intermediates ("X" bands in Fig 1E in that paper, and also in (Goldfarb and Cech 2017). It might be more balanced to conclude, for example, that RMRPP1 knockdown preferentially reduce 40S to 60S rRNA, highlighting that human pre-rRNA processing pathways are incompletely understood.

Fig. 5 Panels C and D: Polysome analyses greatly enhance differences in subunit abundance, since most subunits are in the form of 80S ribosomes. Given that the authors stress the 40S;60S imbalance, it would be helpful to better quantify the actual relative abundances. This is addressed with bioanalyzer traces – but largest differences visible in Extended Data Figure 5 appear to be for a small rRNA, presumably 5.8S or a related species. Displaying the results on a gel might be informative.

Fig. 5 Panels E and F: The difficulty in assessing the effects on pre-rRNA processing from the short read sequencing is that each spacer region is present in multiple pre-rRNA species, and in some cases may also be present as an excised fragment. The effects on pre-rRNA processing is a major conclusion and would have been better assessed by northern hybridization.

If the authors are unable to perfume these analyses, the conclusions, including the abstract should be suitable modified.

Specific points:
4: Introduction, line 38: "The importance of RNase MRP .... result in a variety of human diseases including cartilage hair hypoplasia, metaphyseal dysplasia without hypotrichosis, anauxetic dysplasia, kyphomelic dysplasia and Omenn syndrome".

This sentence could be clarified. Knockout of RMRP is embryonic lethal in mice (and all other systems reported), but human disease associated mutations have not yet been reported in a mouse model. Regarding the associated human diseases, "Omenn syndrome" occurs secondarily to severe combined immune deficiency (SCID) due to T cell deficiency, which in the context of RMRP mutations would normally be classified as part of the CHH spectrum. It might be clearer to say that RMRP mutations are associated with a spectrum of disorders characterised by growth impairment, skeletal dysplasia and immune deficiency.

5: Fig. 2 Panel A: The example microscopy images showing localisation of Rpp14, Rpp21 and C18ORF21 look convincing, but it was not clear how nuclear / nucleolar foci were defined? Could markers for these locations be included and multiple cell images quantified?

Fig. 2 Panel C: This would benefit from also showing RPPH1 RNA reads to assess specificity.

Fig. 2 Panel D: The use of “mean pixel intensity” seems unusual assay for this experiment but seems valid.

6: Fig 4: Please show quantification of replicates of the RT-PCR results (similar to that shown in Fig 2).

Minor point

5: What are the vertical lines in red/green in the PDF of Ext. Fig. 5D?

Reviewer #2 (Remarks to the Author):

The authors have identified the C18orf21 protein, renamed RMRPP1, as a specific component of the RNase MRP RNP in human cells. They demonstrate that RMRPP1 is a nucleolar protein that associates with both the shared protein components of RNase P and RNase MRP and with RNase MRP RNA. They provide evidence that RMRPP1 binds directly to the Rpp29 component shared between RNase P and RNase MRP. Using CRISPR/Cas9 knock down of RMRPP1, they could show that the RMRPP1 protein is required for normal growth and protein synthesis. These effects likely stem from reduced 40S ribosomal subunit accumulation, probably resulting from aberrant pre-rRNA processing, as demonstrated by an increase in the levels of pre-rRNAs containing 5’ETS and ITS1 sequences. Finally, the authors demonstrate that the multi-functional protein Nepro also interacts with RNase MRP but not with RNase P. They go on to show that a mutant version of Nepro associated with anauxetic dysplasia, NeproL145F, displays reduced interactions with RNase MRP specifically. Altogether, these results based on state of the art approaches, will be of great interest to a broad readership. However, I do feel that some data need to be improved, for all the claims of the authors to be supported:

- Figure 2D/E: RT-PCR experiments are probably not the best approach to study the interactions between RMRPP1 and H1 or MRP RNAs, due to the amplification steps. A standard northern analysis of the precipitated RNAs would have been much cleaner. In the experiment shown, there seems to be a substantial interaction of RMRPP1 with H1 RNA.

- Figure 3C: A specificity control is missing here. The authors could have used a RMRPP1 mutant lacking the two N-terminal helices proposed to interact with Rpp29.

- Figure 4B: the MRP RNA signal obtained with RMRPP1N-term+Rpp21C-term seems only slightly higher than background. Quantification of the data might have helped. Wild-type Rpp21 and RMRPP1 control samples should have been included in the same experiment and shown on the same gel to ascertain whether the chimeras have the same affinity for the RNAs tested as the wild-type proteins. Again, northern experiments would have been far more appropriate (same comment for Figure 4E).

- Figure 4E: It would have been better to have all the samples run on the same gel with negative as well as wild-type Rpp21 and RMRPP1 IP samples included. Based on the data presented on this figure, it seems that wild-type RMRPP1 pulls down a significant amount of H1 RNA compared to the GFP control, which contradicts the claim of the authors that RMRPP1 is specific to RNase MRP. The authors should comment. Moreover, contrary to the authors’ conclusion, it is not clear that the Rpp21 RMRPP1 linker chimera pulls down MRP RNA to a significant extent above background.

- Figure 5: Unless I am mistaken, nowhere have the levels of RMRPP1 been analysed following CRISPR/Cas9 targeting of RMRPP1. Was there any residual expression of the protein? Moreover, and this is probably my main criticism of the work, the authors systematically equate loss of RMRPP1 with total loss of RNase MRP activity. It would be necessary to determine whether loss of RMRPP1 impacts (i) MRP RNA levels (ii) RNase MRP assembly (iii) site 2 cleavage in the pre-rRNA. It is entirely possible that without RMRPP1, RNase MRP retains some activities that could explain why 60S biogenesis is not impacted.

- Figure 6F: Nepro knock down does not seem to have a very significant effect on polysome levels and also leads to a huge increase in the levels of 80S ribosomes. The authors should comment.

Minor points:
Figure 4A, bottom chimera: it should be ‘Rpp21 C-term’
Figure 4E, last lane ‘linker IP’ not ‘ilnker IP’
Line 240: remove second ‘that’

Reviewer #3 (Remarks to the Author):

RNase MRP is an essential catalytic ribonucleoprotein complex found in all eukaryotes. RNase MRP is known to be involved in the maturation of rRNA as well as other poorly characterized RNA processing activities. RNase MRP has evolved from RNase P, a ubiquitous RNA-based enzyme involved in the maturation of tRNA and found in all domains of life. RNase MRP and RNase P have related, but distinct catalytic RNA components and share most of their protein subunits.

The best characterized RNase MRP is the S. cerevisiae enzyme, for which cryo-EM structural data is now available. Yeast RNase MRP shares 8 of its protein subunits with yeast RNase P and has two proteins (Rmp1 and Snm1) that are unique to

RNase MRP (one of which, Snm1, is a homologue of a RNase P-specific protein). Human RNase MRP was known to include homologues of proteins shared between yeast RNase MRP and RNase P; these proteins are shared by human RNase MRP and RNase P as well. At the same time, no proteins that are components of human RNase MRP, but not of human RNase P were known up until recently. Accordingly, it was not clear if human RNase MRP possesses proteins that would correspond to yeast Rmp1 and Snm1.

In this manuscript, the authors convincingly demonstrate that two human proteins, a previously uncharacterized C18orf21, and Nepro are bona fide components of human RNase MRP, but not RNase P, demonstrating similarities to and likely playing the role of yeast Rmp1 and Snm1. Additionally, the authors leverage the discovery of RNase MRP (but not RNase P)-specific proteins to test the role of RNase MRP in human cells.

The work is technically solid, and the major conclusions are apparently most certainly valid. This manuscript answers an important question that remained opened for decades and will serve as a foundation for further studies of the structure and function of human RNase MRP. Accordingly, this manuscript will be of substantial interest to the experts in the field RNase P/MRP.

It should be noted that this manuscript is one of three works that have appeared in bioRxiv since January 2025 aiming to identify previously unknown human RNase MRP components. All three studies used different approaches and are unquestionably original. It is comforting that all three studies, including this one, arrive at exactly the same conclusions, identifying exactly the same previously unknown human RNase MRP components; this synergy further validates the result.

This reviewer has only minor comments that should be addressed to improve the manuscript.

General comment: the title of the manuscript significantly overstates the actual reach and significance of the reported results; it would be advisable to change it to something more factual.
Ln 39. Protein and even promoter regions mutations are also associated with dysplasias.
Ln 42. The role of RNase MRP in 5.8S rRNA processing is not essential, at least in yeast: the formation of the longer form of 5.8S rRNA does not appear to depend on RNase MRP.
Ln 47. PRORPs?
Ln 49. H1 RNA/RMRP are the name reserved for the human RNAs specifically; these are not appropriate names for the duplicated genes. Please rephrase.
Ln 58-60. Not a really meaningful statement.
Ln 62,63. Same as above
Ln 65. Role for this protein or for the whole RNase MRP complex that requires it?
Ln 66. Consider rephrasing
Ln 69,70. This is an overstatement
Ln 74,75 Consider rephrasing
Ln 163. Suggesting a direct interaction in the context of the RNase MRP holoenzyme : this is a strong indication, but not a real evidence or “illustration”.
Ln 247,248. How does this compare to what is known for yeast? See also Lindahl et al. 2009 and Li et al. 2021.
Ln 305. The authors may want to be a bit more cautious with this statement as potential alternative roles for RMRPP1 cannot be completely excluded at this point.
Ln 309. See comment to ln 39.
Ln 339. …is likely to have an altered interaction…
Ln 346-348. The authors may want to revisit this sentence.
Ln 359. Preserving ALL other roles? Please rephrase as it is an overstatement as it is: not ALL Nepro roles have been analyzed.
Ln 367. Substrate recognition by yeast RNase MRP has been previously studied biochemically: see Esakova et al. 2011 and 2013.
Ln 424 and below. Was the DNA/protein sequence altered?
Ln 498. Indicating acceleration (as opposed to rpms) would be more useful.
Ln 503. Precision protease? Please correct.

Version 1:

Decision Letter:

23rd Jun 2025

Dear Dr. Cheeseman,

Thank you again for submitting your manuscript "C18orf21/RMP24 and Nepro/RMP64 are RNase MRP-specific subunits required for 40S ribosome biogenesis". We now have comments (below) from the 3 reviewers who evaluated your paper. In light of those reports, we remain interested in your study and but would like to see the manuscript revised to address one outstanding concern.

You will see that while the three reviewers appreciate the quality of the revisions, Reviewer #1 finds the added FISH data inconsistent with the other conclusions of the manuscript. Please be sure to address/respond to all concerns of the referees in full in a point-by-point response and highlight all changes in the revised manuscript text file. If you have comments that are

intended for editors only, please include those in a separate cover letter.

We are committed to providing a fair and constructive peer-review process. Do not hesitate to contact us if there are specific requests from the reviewers that you believe are technically impossible or unlikely to yield a meaningful outcome.

We expect to see your revised manuscript within 6 weeks. If you cannot send it within this time, please contact us to discuss an extension; we would still consider your revision, provided that no similar work has been accepted for publication at NSMB or published elsewhere.

As you already know, we put great emphasis on ensuring that the methods and statistics reported in our papers are correct and accurate. As such, if there are any changes that should be reported, please submit an updated version of the Reporting Summary along with your revision.

Please follow the links below to download these files:

Reporting Summary:
https://www.nature.com/documents/nr-reporting-summary.pdf

Please note that the form is a dynamic ‘smart pdf’ and must therefore be downloaded and completed in Adobe Reader.

When submitting the revised version of your manuscript, please pay close attention to our
href="https://www.nature.com/nature-portfolio/editorial-policies/image-integrity">Digital Image Integrity Guidelines. and to the following points below:

-- that unprocessed scans are clearly labelled and match the gels and western blots presented in figures. Please note that all key data shown in the main figures as cropped gels or blots should be presented in uncropped form, with molecular weight markers. While these data can be displayed in a relatively informal style, they must refer back to the relevant figures. These data should be submitted as source data with the last revision, prior to acceptance.
-- that control panels for gels and western blots are appropriately described as loading on sample processing controls
-- all images in the paper are checked for duplication of panels and for splicing of gel lanes.
-- For any revision that includes light microscopy data, we ask our authors to please include a completed light microscopy reporting table [https://www.nature.com/documents/Light_microscopy_reporting_table.xlsx] to ensure the methods are described thoroughly. The table will be available to reviewers and ultimately published should the manuscript be accepted at the journal.

EXTENDED DATA FIGURES

When re-submitting your manuscript, please ensure that any supplementary figures and tables that are crucial to the manuscript’s conclusions are converted into Extended Data figures and tables to increase visibility of these data. Extended Data figures and tables are online-only (present in the online PDF and full-text HTML versions of the paper), peer-reviewed display items that provide essential background to the article but are not included in the main article due to space constraints. A maximum of ten Extended Data display items (figures and tables) is permitted.

Finally, please ensure that you retain unprocessed data and metadata files after publication, ideally archiving data in perpetuity, as these may be requested during the peer review and production process or after publication if any issues arise.

If there are additional or modified structures presented in the final revision, please submit the corresponding PDB validation reports.

SOURCE DATA: we request that author provide, in tabular form, the data underlying the graphical representations used in figures. This is to further increase transparency in data reporting, as detailed in this editorial (http://www.nature.com/nsmb/journal/v22/n10/full/nsmb.3110.html). Spreadsheets can be submitted in excel format. Only one (1) file per figure is permitted; thus, for multi-paneled figures, the source data for each panel should be clearly labeled in the Excel file; alternately the data can be provided as multiple, clearly labeled sheets in an Excel file. When submitting files, the title field should indicate which figure the source data pertains to. We encourage our authors to provide source data at the revision stage, so that they are part of the peer-review process.

Data availability: this journal strongly supports public availability of data. All data used in accepted papers should be available via a public data repository, or alternatively, as Supplementary Information. If data can only be shared on request, please explain why in your Data Availability Statement, and also in the correspondence with your editor. Please note that for some data types, deposition in a public repository is mandatory - more information on our data deposition policies and available repositories can be found below:
https://www.nature.com/nature-research/editorial-policies/reporting-standards#availability-of-data

We require deposition of coordinates (and, in the case of crystal structures, structure factors) into the Protein Data Bank with the designation of immediate release upon publication (HPUB). Electron microscopy-derived density maps and coordinate data must be deposited in EMDB and released upon publication. Deposition and immediate release of NMR chemical shift

assignments are highly encouraged. Deposition of deep sequencing and microarray data is mandatory, and the datasets must be released prior to or upon publication. To avoid delays in publication, dataset accession numbers must be supplied with the final accepted manuscript and appropriate release dates must be indicated at the galley proof stage.

While we encourage the use of color in preparing figures, please note that this will incur a charge to partially defray the cost of printing. Information about color charges can be found at http://www.nature.com/nsmb/authors/submit/index.html#costs

Nature Structural & Molecular Biology is committed to improving transparency in authorship. As part of our efforts in this direction, we are now requesting that all authors identified as 'corresponding author' on published papers create and link their Open Researcher and Contributor Identifier (ORCID) with their account on the Manuscript Tracking System (MTS), prior to acceptance. This applies to primary research papers only. ORCID helps the scientific community achieve unambiguous attribution of all scholarly contributions. You can create and link your ORCID from the home page of the MTS by clicking on 'Modify my Springer Nature account'. For more information please visit please visit www.springernature.com/orcid.

Please use the link below to submit your revised manuscript and related files:

Link Redacted

Note: This URL links to your confidential home page and associated information about manuscripts you may have submitted, or that you are reviewing for us. If you wish to forward this email to co-authors, please delete the link to your homepage.

We look forward to seeing the revised manuscript and thank you for the opportunity to review your work.

Sincerely,
Sara

Sara Osman, Ph.D.
Senior Editor
Nature Structural & Molecular Biology

Reviewers' Comments:

Reviewer #1 (Remarks to the Author):

The revised paper has been significantly improved, and my specific comments have generally been suitably addressed.

I am, however, surprised by the new FISH results shown in the response to referees and Ext. Fig. 6D-F. The 5.8S and 28S rRNAs are expected to be stably base-paired in the pre-rRNA prior to separation, and would not be expected to show differential localization. In bacteria and archaea, they are both regions of the 23S rRNA. Accumulation of 5.8S in the absence 28S would appear to require substantial 28S degradation, which is inconsistent with the other conclusions of the paper. It seems likely that there is some artefact here and this issue should at least be mentioned in the text.

Reviewer #2 (Remarks to the Author):

The authors have provided an extensively revised version of their original manuscript with substantial text modifications and numerous additional data. They have in my view appropriately answered all the issues raised by the reviewers.

Reviewer #3 (Remarks to the Author):

The authors have addressed all concerns and suggestions that this reviewer had. The revised version of manuscript is substantially improved. Of note, the main conclusion of this work is consistent with the recently published results on the protein composition of human RNase MRP that have been obtained using a different approach (Alexandrov group, Cell Rep. 44(6) 115752).

Version 2:

Decision Letter:

Our ref: NSMB-A50464B

31st Jul 2025

Dear Dr. Cheeseman,

Thank you for submitting your revised manuscript "C18orf21/RMP24 and Nepro/RMP64 are RNase MRP-specific subunits required for 40S ribosome biogenesis" (NSMB-A50464B). It has now been seen by the original referees and their comments are below. The reviewers find that the paper has improved in revision, and therefore we'll be happy in principle to publish it in Nature Structural & Molecular Biology, pending minor revisions to satisfy the referees' final requests and to comply with our editorial and formatting guidelines. Particularly, data in Extended Figure 6 needs to be called out in the text and 1-2 sentences should be added to discuss/clarify the observed inconsistencies. Please note that addressing this adequately by textual clarification (rather than ommission of mention) will be critical for the final acceptance of the manuscript.

We are now performing detailed checks on your paper and will send you a checklist detailing our additional editorial and formatting requirements in the next few weeks. Please do not upload the final materials and make any revisions until you receive this additional information from us.

Thank you again for your interest in Nature Structural & Molecular Biology Please do not hesitate to contact me if you have any questions.

Sincerely,
Sara

Sara Osman, Ph.D.
Senior Editor
Nature Structural & Molecular Biology

Reviewer #1 (Remarks to the Author):

I am not convinced that the most appropriate way to deal with a question about the data presented is to omit mention of the issue. Ext. Fig. 6F is not now referenced in the text, and should at least be called. However, the point is fairly minor, and I would not wish to block publication on these grounds.

Version 3:

Decision Letter:

15th Sep 2025

Dear Dr. Cheeseman,

We are now happy to accept your revised paper "RNase MRP subunit composition and role in 40S ribosome biogenesis" for publication as an Article in Nature Structural & Molecular Biology.

Acceptance is conditional on the manuscript's not being published elsewhere and on there being no announcement of this work to the newspapers, magazines, radio or television until the publication date in Nature Structural & Molecular Biology.

Over the next few weeks, your paper will be copyedited to ensure that it conforms to Nature Structural & Molecular Biology style. Once your paper is typeset, you will receive an email with a link to choose the appropriate publishing options for your paper and our Author Services team will be in touch regarding any additional information that may be required.

After the grant of rights is completed, you will receive a link to your electronic proof via email with a request to make any corrections within 48 hours. If, when you receive your proof, you cannot meet this deadline, please inform us at rjsproduction@springernature.com immediately.

You will not receive your proofs until the publishing agreement has been received through our system.

Due to the importance of these deadlines, we ask that you please let us know now whether you will be difficult to contact over the next month. If this is the case, we ask you provide us with the contact information (email, phone and fax) of someone who will be able to check the proofs on your behalf, and who will be available to address any last-minute problems.

To assist our authors in disseminating their research to the broader community, our SharedIt initiative provides all co-authors with the ability to generate a unique shareable link that will allow anyone (with or without a subscription) to read the published article. Recipients of the link with a subscription will also be able to download and print the PDF.

As soon as your article is published, you can generate your shareable link by entering the DOI of your article here: http://authors.springernature.com/share. Corresponding authors will also

receive an automated email with the shareable link

Note the policy of the journal on data deposition: http://www.nature.com/authors/policies/availability.html.

Your paper will be published online soon after we receive proof corrections and will appear in print in the next available issue. You can find out your date of online publication by contacting the production team shortly after sending your proof corrections.

You may wish to make your media relations office aware of your accepted publication, in case they consider it appropriate to organize some internal or external publicity. Once your paper has been scheduled you will receive an email confirming the publication details. This is normally 3-4 working days in advance of publication. If you need additional notice of the date and time of publication, please let the production team know when you receive the proof of your article to ensure there is sufficient time to coordinate. Further information on our embargo policies can be found here: https://www.nature.com/authors/policies/embargo.html

You can now use a single sign-on for all your accounts, view the status of all your manuscript submissions and reviews, access usage statistics for your published articles and download a record of your refereeing activity for the Nature journals.

If you have not already done so, we strongly recommend that you upload the step-by-step protocols used in this manuscript to the Protocol Exchange. Protocol Exchange is an open online resource that allows researchers to share their detailed experimental know-how. All uploaded protocols are made freely available, assigned DOIs for ease of citation and fully searchable through nature.com. Protocols can be linked to any publications in which they are used and will be linked to from your article. You can also establish a dedicated page to collect all your lab Protocols. By uploading your Protocols to Protocol Exchange, you are enabling researchers to more readily reproduce or adapt the methodology you use, as well as increasing the visibility of your protocols and papers. Upload your Protocols at www.nature.com/protocolexchange/. Further information can be found at www.nature.com/protocolexchange/about.

An online order form for reprints of your paper is available at https://www.nature.com/reprints/author-reprints.html. Please let your coauthors and your institutions' public affairs office know that they are also welcome to order reprints by this method.

Authors may need to take specific actions to achieve compliance with funder and institutional open access mandates. If your research is supported by a funder that requires immediate open access (e.g. according to Plan S principles or the NIH public access policy) then you should select the gold OA route, and we will direct you to the compliant route where possible. Because authors warrant under our subscription licensing terms that they haven't committed to licensing any version of their article under a licence inconsistent with the terms of our agreement – including the applicable embargo period – publication under the subscription model isn't suitable for authors whose funders require no embargo.

In approximately 10 business days you will receive an email with a link to choose the appropriate publishing options for your paper and our Author Services team will be in touch regarding any additional information that may be required.

You will not receive your proofs until the publishing agreement has been received through our system.

If you have any questions about our publishing options, costs, Open Access requirements, or our legal forms, please contact ASJournals@springernature.com

Sincerely,
Sara

Sara Osman, Ph.D.
Senior Editor
Nature Structural & Molecular Biology

Click here if you would like to recommend Nature Structural & Molecular Biology to your librarian: http://www.nature.com/subscriptions/recommend.html#forms

Open Access This Peer Review File is licensed under a Creative Commons Attribution 4.0 International License, which permits use, sharing, adaptation, distribution and reproduction in any medium or format, as long as you give appropriate credit to the original author(s) and the source, provide a link to the Creative Commons license, and indicate if changes were made.
In cases where reviewers are anonymous, credit should be given to 'Anonymous Referee' and the source.
The images or other third party material in this Peer Review File are included in the article's Creative Commons license, unless indicated otherwise in a credit line to the material. If material is not included in the article's Creative Commons license and your intended use is not permitted by statutory regulation or exceeds the permitted use, you will need to obtain permission directly from the copyright holder.
To view a copy of this license, visit https://creativecommons.org/licenses/by/4.0/

We thank the reviewers for their constructive comments and suggestions. For the revised manuscript, we have made a number of changes to the text and figures in response to these suggestions. In particular, we have added multiple new experiments and analyses that we believe provide additional insights into the mechanisms of RNase MRP function and the consequences of C18orf21 knockouts. These include:

1. ***Detailed analysis of RNase MRP function in 40S vs. 60S ribosome biogenesis***. Prior studies suggested that RNase MRP cleaved rRNA within ITS1, which should affect 40S and 60S ribosome biogenesis similarly. For the previous version of our paper, we identified a clear role for C18orf21 in 40S ribosome biogenesis, but did not observe a similar change in 60S levels. For the revised paper, we performed RNA FISH to analyze 18S, 5.8S, and 28S rRNA levels in RNase MRP defective cells. Consistent with our prior data, we observed a decrease in the cytosolic levels of 18S but not 5.8S or 28S rRNA in RNase MRP knockout cells. However, we observed nucleolar accumulation of 5.8S in RNase MRP defective cells, suggesting some role of RNase MRP in 5.8S rRNA biogenesis. Additionally, we have now conducted an extensive analysis of sequencing reads that span rRNA cleavage site junctions. Based on this work, we find that indeed, RNase MRP substantially affects 40S ribosome biogenesis. We additionally show a minor effect on 60S ribosome biogenesis. Therefore, we conclude that RNase MRP promotes both 40S and 60S biogenesis but *preferentially* affects 40S maturation.
2. ***Additional rare disease mutations in NEPRO affect their RNase MRP association.*** In our previous manuscript, we identified several NEPRO mutations that may impair their association with the RNase MRP complex. We tested a single mutation in NEPRO and found that this mutation reduced its association with RNase MRP. For the revised version, we use quantitative proteomics to systematically test multiple of the identified rare disease mutations in NEPRO. Consistent with our prior work, this data demonstrates that these mutations can weaken RNase MRP binding, strengthening our model of a mutational "hotspot" underlying RNase MRP-associated diseases.
3. ***RNase MRP RNA levels and complex assembly in C18orf21 knockouts.*** Our prior data suggested that C18orf21 is required for RNase MRP activity. To investigate the underlying molecular mechanisms, for the revised paper we examined RNase MRP RNA levels and complex assembly. Using RNA-seq and RT-qPCR, we measured MRP RNA levels in knockout cells and now show that C18orf21 is essential for MRP RNA stability. Additionally, our analysis of RNase MRP subunit localization in C18orf21-deficient cells reveals that RPP14 fails to localize to the nucleolus in the absence of C18orf21. Together, these findings suggest that C18orf21 promotes RNase MRP activity at least in part by stabilizing the MRP RNA and facilitating complex association with the nucleolus.
4. ***Biochemical analysis of C18orf21-RPP29 interface***. In our previous manuscript, we reported a direct interaction between C18orf21-RPP29 through bacterial co-purifications. For the revised paper, we leveraged our structural model and deleted the predicted RPP29 interaction surface in C18orf21. We now show that deletion of this surface on C18orf21 does indeed eliminate its interactions with RPP29.

5. ***Analysis of RPP14 knockout phenocopies C18orf21.*** Our previous manuscript found that loss of C18orf21 leads to the accumulation of unprocessed rRNA species. To test whether this defect arises from impaired RNase MRP activity, we have now additionally knocked out RPP14, a known RNase MRP component. Our results demonstrate that C18orf21 and RPP14 knockouts display highly similar rRNA processing defects. In addition to the similar rRNA processing defects in RPP14 and C18orf21 knockouts, we performed both total RNA and mRNA sequencing and saw that these two knockout lines have very similar transcriptome phenotypes. The major difference observed was the loss of H1 RNA in RPP14 knockouts compared to C18orf21, consistent with the dual-function of RPP14 and single function of C18orf21.
6. ***Additional evidence that C18orf21 is specifically required for RNase MRP but not RNase P activity.*** One of reviewers asked whether C18orf21 may also bind to H1 RNA such that it plays a role in RNase P function. By analyzing RNase P activity through MALAT1 cleavage, we now show that C18orf21 is dispensable for RNase P activity.
7. ***C18orf21 depletion induces apoptosis***. Our existing data based competitive growth assays suggested that C18orf21 depletion decreased cellular fitness. Prior literature had conflicting data on whether RNase MRP defects resulted in slower growth rates or was required for viability. For the revised paper, we now show that C18orf21 knockout induces apoptosis and cell death in addition to the decreased growth rates. Our analysis indicates that C18orf21 is an essential gene and that some of the prior analyses of RNase MRP were likely partial loss of function alleles.
8. ***Analysis of Cas9-depletion efficiencies and phenotypic rescue***. For the revised paper, we have now analyzed the efficiency of C18orf21 knockout on the protein, RNA, and DNA level. Cas9-mediated depletion of C18orf21 efficiently eliminates >90% of the protein.
9. ***Clarification and quantification of RNase P foci.*** Our prior data demonstrated that RNase P was present in nuclear foci. We have now clarified our definition of RPP21 foci and quantified the number of foci in each cell.
10. ***Quantification and additional replicates of RNA-immunoprecipitation experiments.*** We have now performed additional replicates and quantified the relative ratio of RMRP and H1 RNA in the C18orf21-RPP21 chimeric constructs.
11. ***Clarification of NEPRO knockout cells.*** We now show that NEPRO knockout results in a decrease of the monosome/polysome ratio and a depletion of 40S ribosomes, consistent with the C18orf21 knockout.
12. ***Gene names and nomenclature.*** Based on recent discussions with the HUGO Gene Nomenclature Committee, we have updated the gene names for the RNase MRP subunits to conform with agreed upon guidelines. We refer to C18orf21 as RMP24 and additionally refer to NEPRO as RMP64.

Below, we have included detailed point-by-point responses to each reviewer comment.

Reviewer #1:

1: *Both C18ORF21 and C3ORF17 were previously identified as RNase MRP interacting proteins based on pulldown of the RMRP RNA. See Figures 1B and 1C in: [https://www.nature.com/articles/s41586-018-0453-z.](https://www.nature.com/articles/s41586-018-0453-z)*
MRP interactions were also predicted from bioinformatics: https://bmcbioinformatics.biomedcentral.com/articles/10.1186/s12859-023-05425-7
These findings were apparently not pursued, and this does not substantially detract from novelty of the current report. They should, however, be acknowledged.

We thank the reviewer for pointing out these studies. We have now cited and acknowledged both of these papers. We have also cited a recent large-scale study (PMID: 40205054) that suggested C18orf21 as possibly associating and functioning with RNase MRP/P.

2: *Knockout cells models: it might have been anticipated that C18ORF21knockout cells would not be viable. The assays were reportedly done on a puromycin selected pool of cells. Could validation data be shown to give an indication of % expression of C18ORF21 mRNA and protein in these pools?*

We thank the reviewer for these suggestions. To validate the loss of C18orf21 in our knockout cells, we have now performed western blots using an anti-C18orf21 antibody (compare lane 1 and 3 below). This data has been added to Extended Data Figure 5.

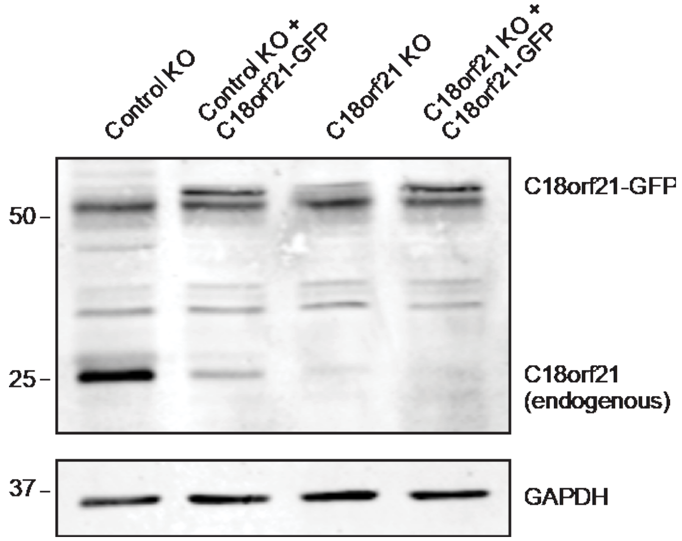

To assess knockout efficiency, we also performed sanger sequencing-based ICE (Synthego) analysis to monitor the Cas9 cleavage efficiency for C18orf21 knockouts. This analysis revealed that the knockout efficiency of our system is 80% (Extended Data Figure 5B and shown below).

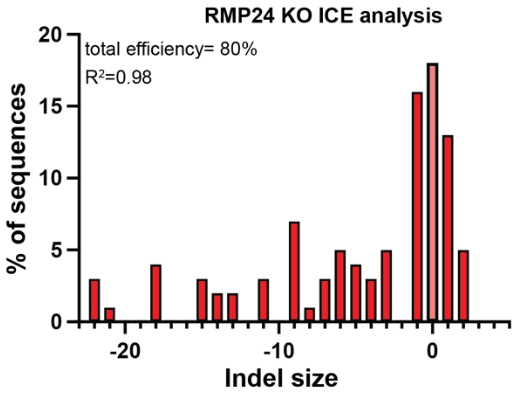

To assess the levels of the target mRNA, we compared the normalized read counts in control and C18orf21 KO cells. We found that there is no difference in mRNA levels between these two populations of cells consistent with Cas9 acting to generate mutations, but not affect mRNA expression. We also performed a similar analysis of RNA-sequencing data from RPP14 and RPP21 knockout cells and did not find changes in the RNA levels for the Cas9 targeted gene. This suggests that the indels inserted by Cas9-mediated cleavage and repair do not induce nonsense mediated decay.

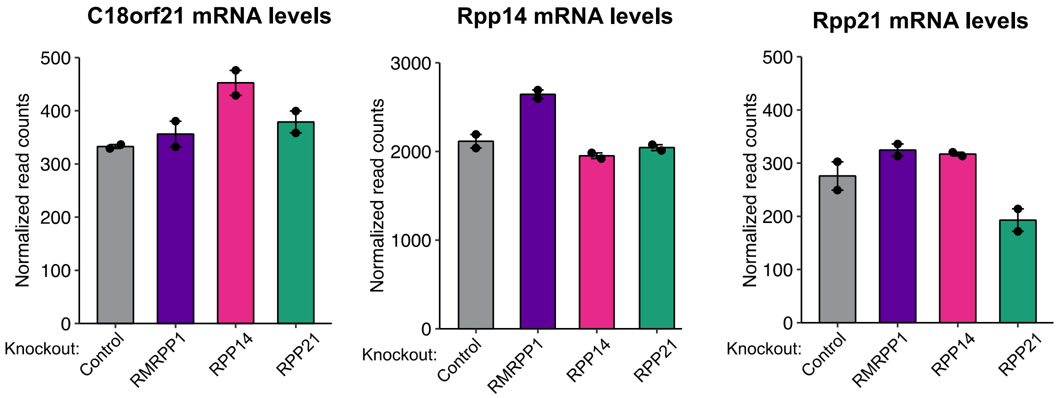

Finally, to validate that our C18orf21 knockout system is specific, we performed rescue experiments for our polysome analysis. We ectopically expressed C18orf21-GFP in C18orf21 knockout cells (western blot shown above) and were able to rescue the defect in 40/60S levels along with the polysome/monosome defects.

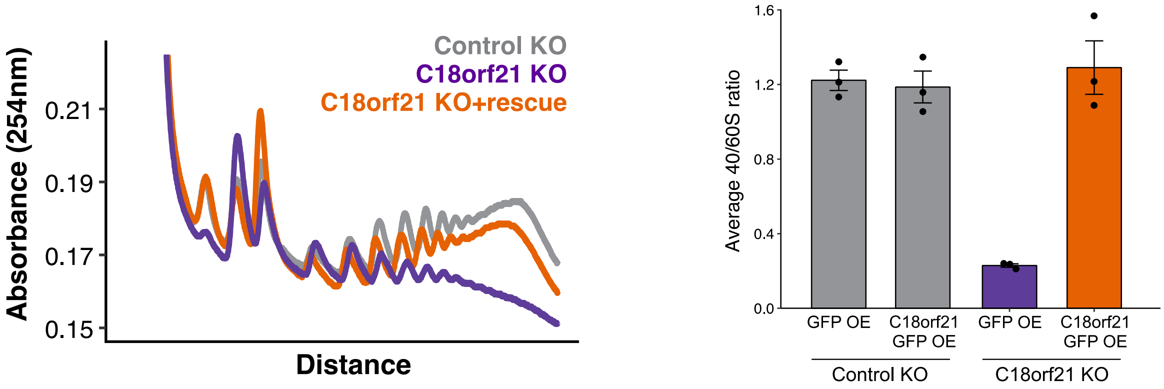

3: *For the growth competition assay, it would be helpful to have data to understand if the RMPP1 "KO" cells are dying (as reported for knockout of RMRP eg in Goldfarb and Cech. 2017) or alive, but growing more slowly (as seen in patient mutations in RMRP).*

We agree with the reviewer that it would be informative to understand the basis for the fitness disadvantage observed in our competitive growth assays. To determine whether C18orf21 knockout cells are dying or have an impaired growth rate, we stained C18orf21 knockout cells with a marker for apoptosis, annexin V, and a marker for cell death, propidium iodide. After staining, we performed FACS to determine the populations of live, apoptotic, and dead cells (see figure below). We found that ~20% of C18orf21 knockout cells displayed evidence of apoptosis or cell death after five days of knockout induction. This cell death is likely stochastic with knockout cells dying at various time points, but then being lost from the population. Together, this data strongly suggests that the fitness defect observed in our growth competition assay results from a combination of cell death and decreased cell growth, matching more closely to the phenotype observed in the *RMRP* knockout.

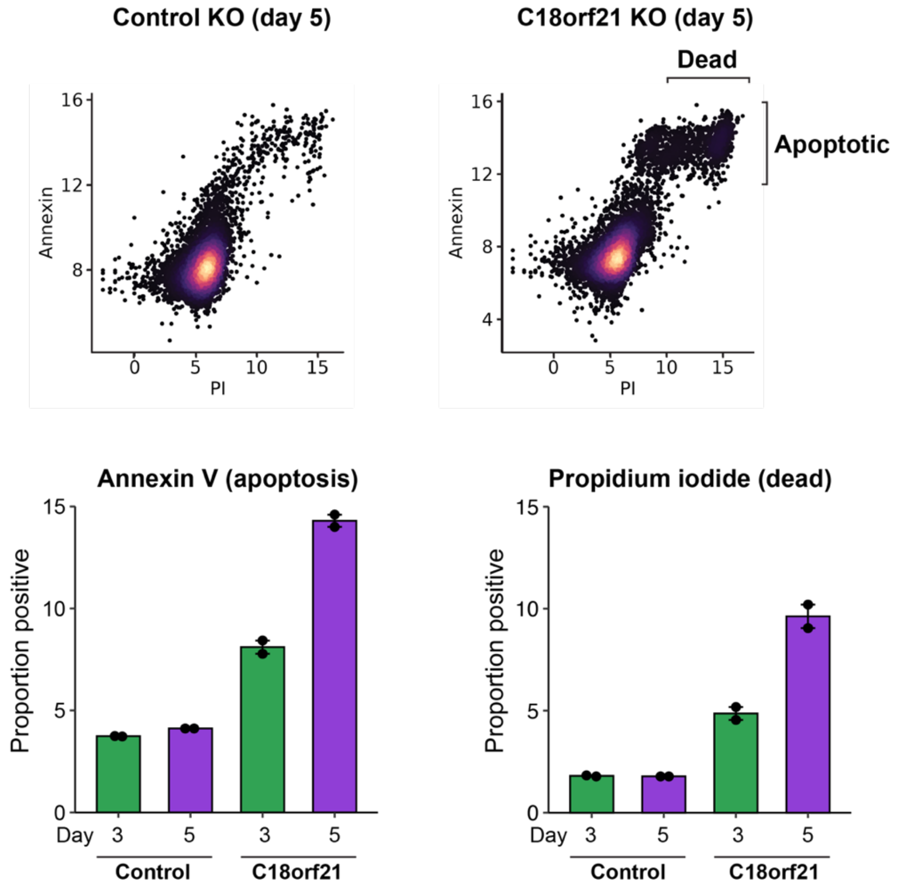

4: *The conclusion in abstract (line 19) and elsewhere in text that "...we reveal that RNase MRP is required for 40S, but not 60S, ribosome biogenesis, uncovering an alternative pathway for ribosome assembly" is stronger than the presented data can support.*

The data support some reduction in 40S:60S ratios the KO cell lines, but no data is shown on what this pathway would. Moroeover, the data presented show ratios of 60S to 40S, making it unclear that 60S production is unaffected, only that 40S is preferentially affected. These difficulties in interpretation are compounded by the uncertainty whether the defects are being studies in viable or dying cells.

This conclusion would also contrast with a previous report from cells with disease-associated mutations in the RMRP RNA where 18S and 28S rRNA were approximately equally reduced (Robertson et al, 2022). Also of note are previous findings that RMRP knockout in mammalian cells causes the appearance of undefined rRNA processing intermediates ("X" bands in Fig 1E in that paper, and also in (Goldfarb and Cech 2017). It might be more balanced to conclude, for example, that RMRPP1 knockdown preferentially reduce 40S to 60S rRNA, highlighting that human pre-rRNA processing pathways are incompletely understood.

We thank the reviewer for this comment. To strengthen our conclusions about RNase MRP in 40S and 60S ribosome biogenesis, we have now performed RNA fluorescence in situ hybridization (FISH) on 18S, 5.8S, and 28S rRNA to analyze changes in the subcellular abundances. We observed a striking decrease in cytoplasmic 18S rRNA in C18orf21 knockout cells, along with an accumulation of nucleolar 18S rRNA. Furthermore, we did not observe any changes in cytoplasmic levels of 5.8S and 28S rRNA (Extended Data Fig. 7D-F and below). This is consistent with our prior observation of decreased 40S ribosome with only minor effects on the 60S ribosome in C18orf21 knockout cells. However, we did observe a nucleolar accumulation of the 5.8S rRNA, consistent with a decrease in ITS1 cleavage. These results are consistent with our model that 40S ribosome biogenesis is preferentially affected over 60S in RNase MRP defective cells. As the reviewer correctly hypothesized, our analysis suggests that the 60S biogenesis is modestly affected. However, 40S biogenesis is much more substantially affected. We have added this data to Figure 4 and we have updated the text to conclude that C18orf21 knockdown preferentially reduces the 40S to 60S ribosome ratio.

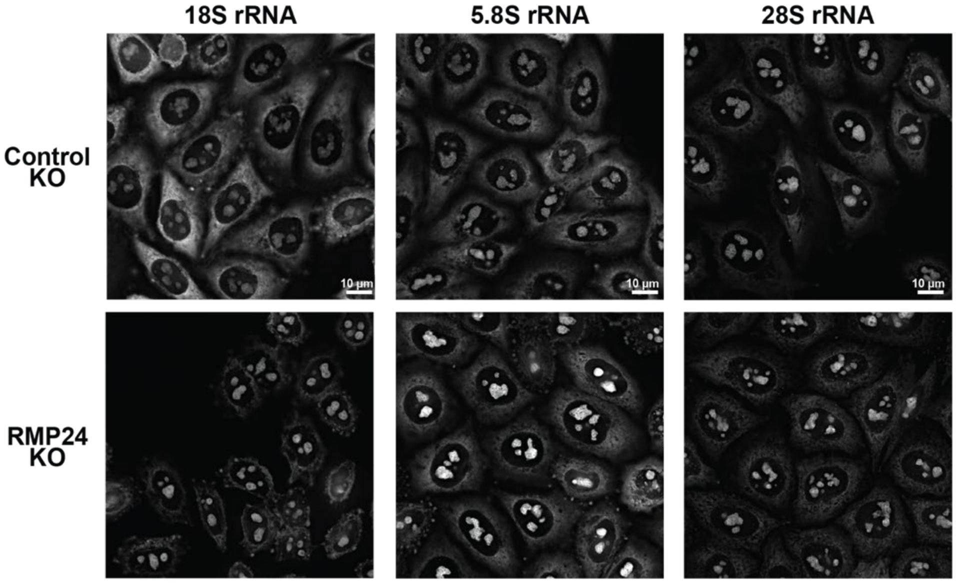

5: *Fig. 5 Panels C and D: Polysome analyses greatly enhance differences in subunit abundance, since most subunits are in the form of 80S ribosomes. Given that the authors stress the 40S;60S imbalance, it would be helpful to better quantify the actual relative abundances. This is addressed with bioanalyzer traces – but largest differences visible in Extended Data Figure 5 appear to be for a small rRNA, presumably 5.8S or a related species. Displaying the results on a gel might be informative.*

We thank the reviewer for this suggestion. Based on this comment, we ran RNA from control and C18orf21 knockout cells on an agarose gel (see figure below). We found an increased ratio of 28S rRNA to 18S rRNA in C18orf21 knockout cells compared to control cells matching the trends observed in the bioanalyzer traces in Extended Data Figure 6.

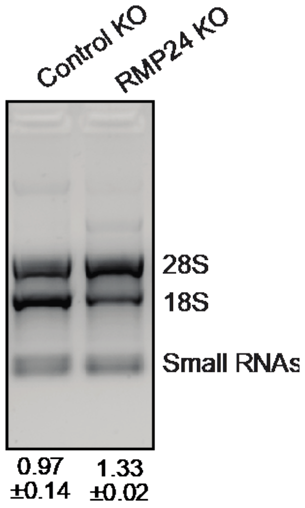

We have now included a quantitative analysis of bioanalyzer traces to more clearly illustrate the observed differences. Additionally, to further validate this modest effect, we assessed 28S/18S rRNA ratios using a second C18orf21-targeting guide RNA, which similarly increased the 28S/18S ratio as detected by bioanalyzer.

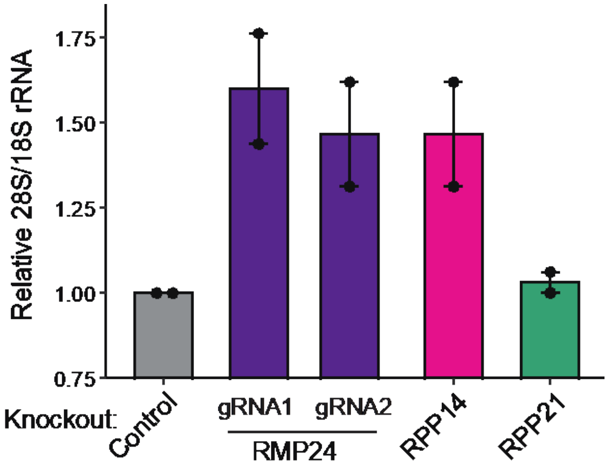

6: *Fig. 5 Panels E and F: The difficulty in assessing the effects on pre-rRNA processing from the short read sequencing is that each spacer region is present in multiple pre-rRNA species, and in some cases may also be present as an excised fragment. The effects on pre-rRNA processing is a major conclusion and would have been better assessed by northern hybridization.*

If the authors are unable to perfume these analyses, the conclusions, including the abstract should be suitable modified.

We agree with the reviewer that an orthologous approach to test the effects of knocking out C18orf21 on rRNA processing would help strengthen a major conclusion of the manuscript. We have now performed an additional analysis to define the rRNA processing defects that occur following the knockout of RNase MRP components. To address whether the ITS1 reads represent stable excised fragments rather than precursor RNA, we analyzed reads that spanned junctions (20 nucleotides flanking the junctions) of rRNA. Importantly, our sequencing read length was 150x150 bp such that we were able to identify thousands of reads that spanned junctions. We found that the 18S-ITS1 junction reads were enriched in C18orf21 knockout cells, consistent with our prior analyses and conclusion. To further validate this, we performed rRNA junction analysis of RPP14 (RNase MRP/P) and RPP21 (RNase P specific) knockout cells. Consistently, knockout of RPP14, but not the RNase P subunit RPP21, similarly showed an increase in 18S-ITS1 junction reads. This approach is analogous to classical splice isoform analysis to look for exon-exon junction reads. We performed this analysis across all regions of rRNA to analyze differences across all steps of rRNA processing. Given the modest changes in 28S rRNA, we have adjusted our wording appropriately.

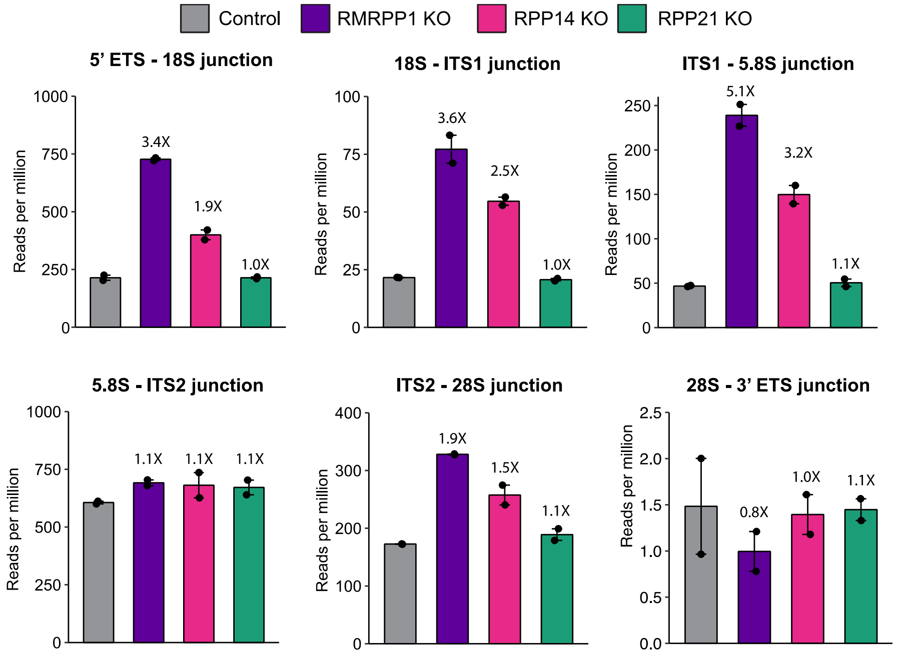

In addition to the analysis of our RNA-sequencing data, we now performed a RT-qPCR-based assay with primers that flank the RNase MRP cleavage site in ITS1. We found an increase in the abundance of uncleaved ITS1 in cells where C18orf21 or RPP14 were knocked out compared to control cells and RPP21 knockout cells. We believe that these data strongly suggests that C18orf21 and RPP14 are required for removal of ITS1, which is consistent with the prior literature on the activity of RNase MRP. This data is shown below and now included in Figure 5.

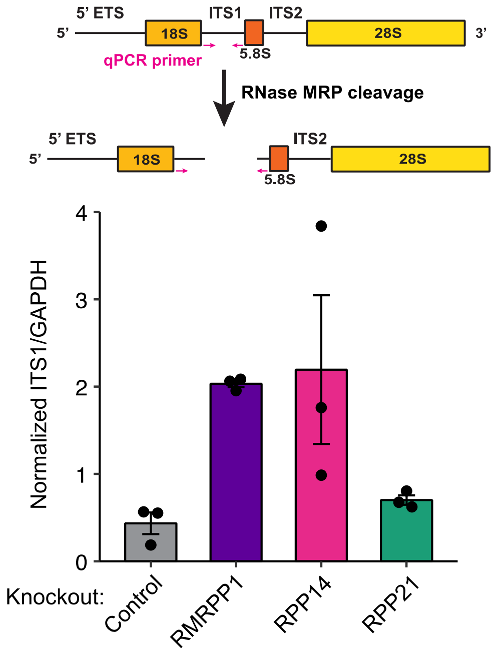

Beyond the next generation sequencing-based analyses, we recognize the value of other approaches such as northern blotting. As our lab was, until recently, primarily a cell division laboratory, we are simply not equipped for these experiments, and this set up also does not exist in neighboring labs. We do note that, in addition to our deep sequencing approach, a preprint that appeared online during the revision process (PMID: 40027791) analyzed rRNA intermediates by Northern blotting. Consistent with our results and the results from the Cech lab MRP RNA knockout (PMID: 28115465), this preprint observed an accumulation of precursor RNAs consistent with cleavage in ITS1. We have now cited this preprint.

7: *Introduction, line 38: "The importance of RNase MRP .... result in a variety of human diseases including cartilage hair hypoplasia, metaphyseal dysplasia without hypotrichosis, anauxetic dysplasia, kyphomelic dysplasia and Omenn syndrome".*

This sentence could be clarified. Knockout of RMRP is embryonic lethal in mice (and all other systems reported), but human disease associated mutations have not yet been reported in a mouse model. Regarding the associated human diseases, "Omenn syndrome" occurs secondarily to severe combined immune deficiency (SCID) due to T cell deficiency, which in the context of RMRP mutations would normally be classified as part of the CHH spectrum. It might be clearer to say that RMRP mutations are associated

with a spectrum of disorders characterised by growth impairment, skeletal dysplasia and immune deficiency.

We thank the reviewer for this suggestion and have updated the text to clarify these points.

8: *Fig. 2 Panel A: The example microscopy images showing localisation of Rpp14, Rpp21 and C18ORF21 look convincing, but it was not clear how nuclear / nucleolar foci were defined? Could markers for these locations be included and multiple cell images quantified?*

Previous studies (Jarrous et al. JCB 1999, Jarrous et al. RNA 2001) suggested that RNase P localized to Cajal bodies. However, when we performed immunofluorescence with the Cajal body marker Coilin, we found that the nuclear puncta associated with RPP21 and RPP14 did not colocalize with Coilin (see figure below). Additionally, in our immunoprecipitation experiments, we did not detect substantial amounts of Cajal body-associated proteins such as Coilin or TCAB1 in IPs for either RPP21 or RPP14 (see figure below). These IP-mass spec experiments also did not identify interaction partners that would be suitable to use as a localization marker for immunofluorescence for RNase P foci.

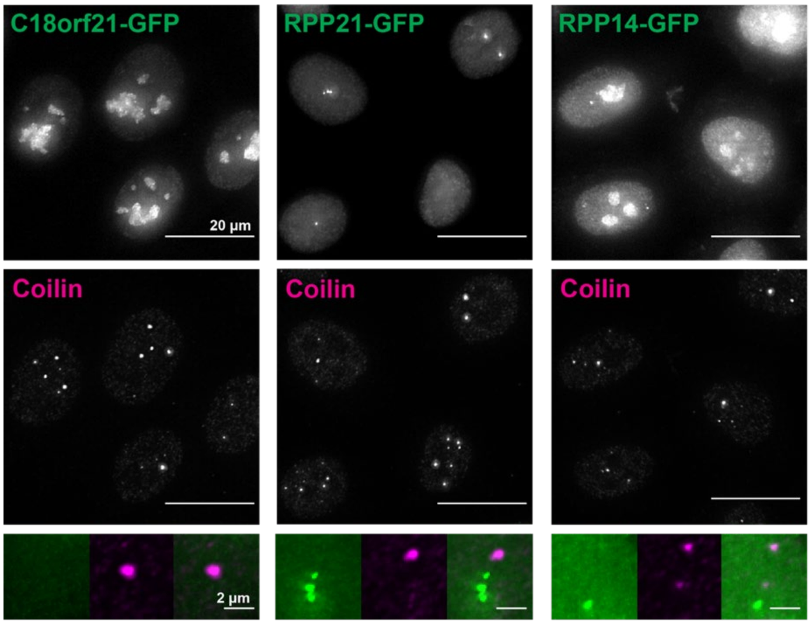

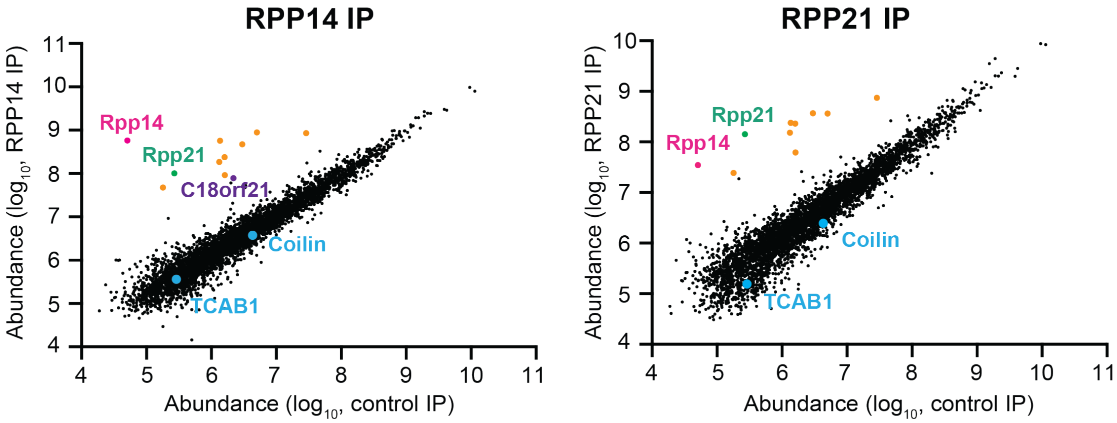

In place of a distinct marker for the specific nuclear foci to which RPP21 and RPP14 localize, we took an alternative approach to analyze its localization using Hoechst to determine the nucleolar vs. nuclear localization of each construct. We evaluated localization to the nucleolus by merging the Hoechst and GFP channels and looking for an enriched GFP signal in the characteristic "holes" in the nuclear staining created by the enriched Hoechst staining around the rim of the nucleoli. We defined nuclear puncta as foci in the GFP channel greater than 0.25 μm in size and with an integrated pixel intensity above background. To quantify the localization of each protein, we counted the number of cells that had nuclear puncta and/or enriched signal in the nucleolus. This data has been added to Extended data Figure 2.

***9:** Fig. 2 Panel C: This would benefit from also showing RPPH1 RNA reads to assess specificity.*

We thank the reviewer for pointing this out. We agree and have now moved that data from Extended Data Figure 2 into the main figure.

***10:** Fig. 2 Panel D: The use of "mean pixel intensity" seems unusual assay for this experiment but seems valid.*

To address this reviewer's comment, we have now quantified the raw integrated pixel density. We found that the results were consistent with our measurement of mean pixel intensity. We have updated the figure to include this quantitation method and have quantified the replicates for Figure 4 (also see point 11) in the same manner.

***11:** Fig 4: Please show quantification of replicates of the RT-PCR results (similar to that shown in Fig 2).*

We have now added the quantification from three replicates to Figure 4.

***12:** What are the vertical lines in red/green in the PDF of Ext. Fig. 5D?*

We thank the reviewer for pointing this out. The red and green vertical lines correspond to mutations identified in the sequencing reads compared to the genome annotations. We have now updated the figure legend to clarify this point.

Reviewer #2:

1: *Figure 2D/E: RT-PCR experiments are probably not the best approach to study the interactions between RMRPP1 and H1 or MRP RNAs, due to the amplification steps. A standard northern analysis of the precipitated RNAs would have been much cleaner. In the experiment shown, there seems to be a substantial interaction of RMRPP1 with H1 RNA.*

We agree with the reviewer that additional strategies to detect interactions of the RNase MRP and RNase P proteins with the RNase MRP and H1 RNAs would be useful. However, as orthologous approaches we have used RNA-sequencing methods and RT-PCR to analyze the abundance of RNase MRP and H1 RNAs. Although we recognize that RT-PCR may amplify low amounts of RNA, we believe RNA-sequencing has advantages over northern blotting. RNA-seq provides the power of unbiased and quantitative transcriptome analysis, allowing us to detect all RNA species pulled down by the C18orf21. For this paper, we systematically analyzed RNase MRP-bound RNAs followed by validation with RT-PCR. In the updated manuscript, we have tried to better emphasize that we have used multiple methods to quantitatively measure RNA levels.

As an alternate approach to evaluate any role for C18orf21 in RNase P function, we have now tested whether the modest amount of H1 RNA detected in C18orf21 IPs is functional or background. To do this, we analyzed the cleavage of MALAT1, a known target of RNase P. Our work show that C18orf21 is dispensable for MALAT1 cleavage. This is in contrast to RPP21 (RNase P specific) and RPP14 (RNase MRP/P), where knockout of these proteins results in a dramatic accumulation of uncleaved MALAT1.

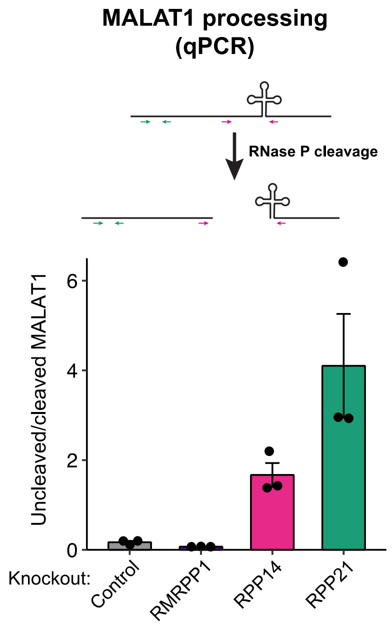

***2:** Figure 3C: A specificity control is missing here. The authors could have used a RMRPP1 mutant lacking the two N-terminal helices proposed to interact with Rpp29.*

We thank the reviewer for this suggestion. We have now co-expressed and purified RPP29 with a construct of C18orf21 that lacks the two N-terminal helices (33-220) in *E. coli*. We found that this construct of C18orf21 did not express well and was degraded (see figure below). Additionally, RPP29 was not soluble when co-expressed with this C18orf21 construct, suggesting that the two proteins no longer interact. We passed the proteins that were eluted off of glutathione beads over an S200 column. The resulting chromatogram showed three major peaks that eluted at later volumes than the C18orf21-RPP29 dimer (see figure below). Although we would expect a dimer of RPP29 and C18orf21 (33-220) to elute later than the complex with full length C18orf21, comparison of these two chromatograms shows a lack of any peak that elutes around at the expected volume. This data suggests that the two N-terminal helices are required for the co-purification of C18orf21 and RPP29. This data has been added to Extended Data Figure 3.

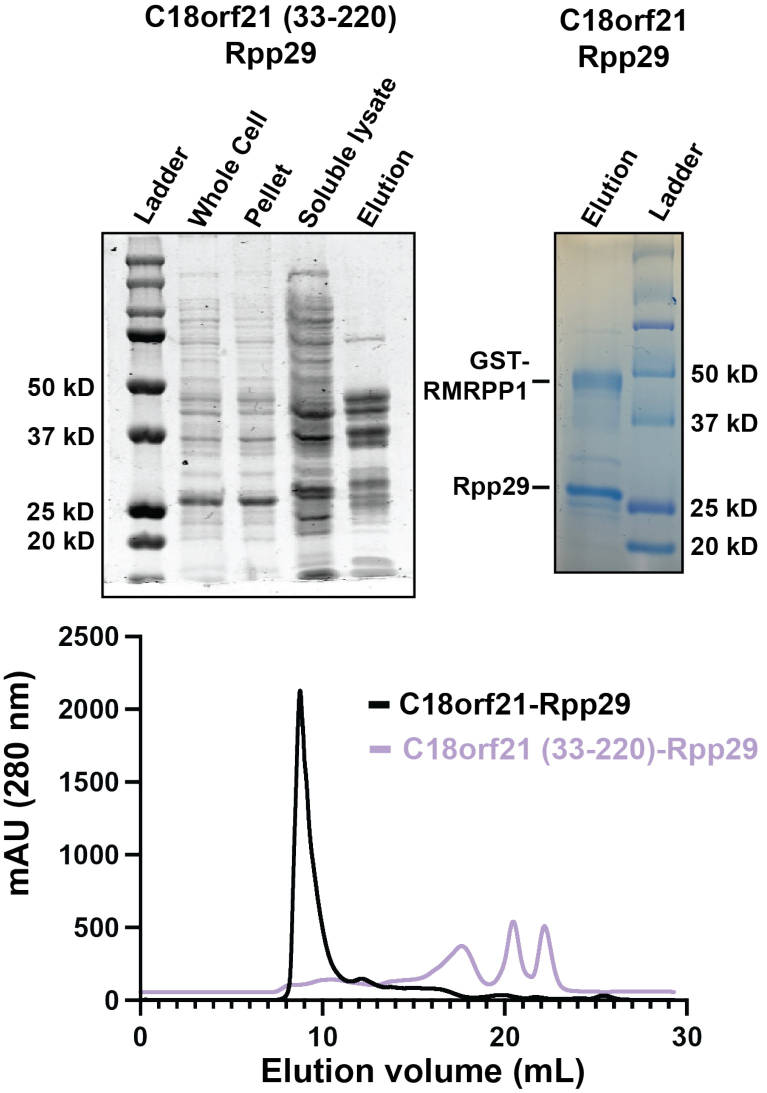

***3:** Figure 4B: the MRP RNA signal obtained with RMRPP1N-term+Rpp21C-term seems only slightly higher than background. Quantification of the data might have helped. Wild-type Rpp21 and RMRPP1 control samples should have been included in the same experiment and shown on the same gel to ascertain whether the chimeras have the same affinity for the RNAs tested as the wild-type proteins. Again, northern experiments would have been far more appropriate (same comment for Figure 4E).*

We understand that northern experiments may provide a visually cleaner result than the RT-PCR experiment because even a small amount of RNA immunoprecipitated can be amplified by the PCR step. However, for several reasons, including technical limitations, we believe that the RT-PCR experiments are sufficient to support our conclusions with the chimeras. First, we think the important comparison is provided by the ratiometric information regarding the relative MRP:H1 RNA levels provided by the assay. We tested each chimera to determine if substituting the predicted structural features of C18orf21 onto RPP21 can change RPP21 from being predominantly an H1 RNA-interacting protein to an RNase MRP-interacting protein. The constructs with ratios that switch from being predominantly H1 RNA to predominantly MRP RNA are the most informative. Second, we validated the results for several of our RT-PCR experiments using RNA immunoprecipitation-RNA sequencing (RIP-Seq) experiments. Third, we now have performed and quantified three replicate experiments for each of the chimeras. This includes an experiment that includes all the chimeras and wild type proteins on a single gel. This data is included below and in an updated version of Figure 4.

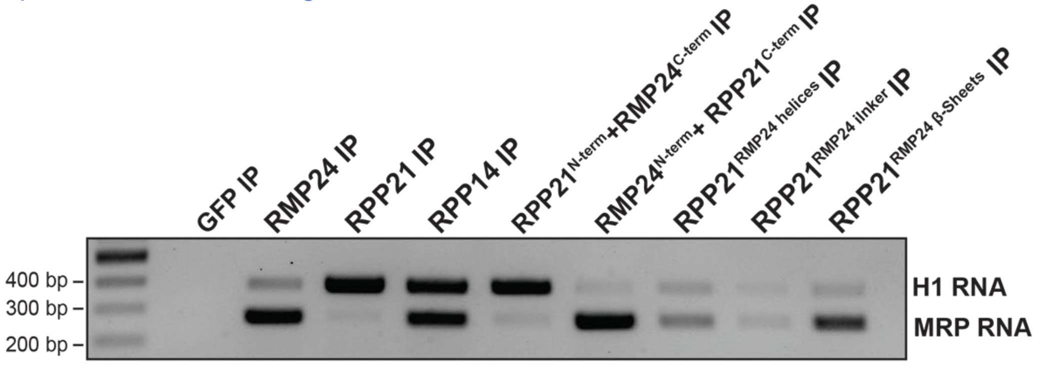

***4:** Figure 4E: It would have been better to have all the samples run on the same gel with negative as well as wild-type Rpp21 and RMRPP1 IP samples included. Based on the data presented on this figure, it seems that wild-type RMRPP1 pulls down a significant amount of H1 RNA compared to the GFP control, which contradicts the claim of the authors that RMRPP1 is specific to RNase MRP. The authors should comment. Moreover, contrary to the authors' conclusion, it is not clear that the Rpp21 RMRPP1 linker chimera pulls down MRP RNA to a significant extent above background.*

We agree with the reviewer and have replaced the gel in Figure 4E with an experiment with all of the samples. We agree that there is a band that is above background in our C18orf21 RIP-PCR experiments. However, we think this a technical artifact. First, our

RNA-sequencing experiments show that C18orf21 only significantly pulls down MRP RNA. Additionally, as mentioned in response to point 1, we have tested how knockout of C18orf21 effects cleavage of MALAT1 using a qPCR-based assay and found that loss of C18orf21 does not affect MALAT1 processing. Additionally, after performing replicates we agree that the linker construct is inefficient at pulling down MRP RNA. We have updated our conclusion about this construct in the text to reflect this.

***5:** Figure 5: Unless I am mistaken, nowhere have the levels of RMRPP1 been analysed following CRISPR/Cas9 targeting of RMRPP1. Was there any residual expression of the protein?*

To validate the loss of C18orf21 in our knockout cells, we have now performed western blots using an anti-C18orf21 antibody. We find that C18orf21 is reduced by >90%. This data has been added to Extended Data Figure 5. In addition to analyzing protein levels, we have now analyzed RNA levels and Cas9 cleavage efficiency (80%). Finally, to further strengthen our results with our C18orf21 knockout cells, we rescued the polysome defects in knockout cells through ectopic expression of C18orf21-GFP. The data corresponding to each of these points can be found in the response to Reviewer 1, point 2.

***6:** Moreover, and this is probably my main criticism of the work, the authors systematically equate loss of RMRPP1 with total loss of RNase MRP activity. It would be necessary to determine whether loss of RMRPP1 impacts (i) MRP RNA levels (ii) RNase MRP assembly (iii) site 2 cleavage in the pre-rRNA. It is entirely possible that without RMRPP1, RNase MRP retains some activities that could explain why 60S biogenesis is not impacted.*

We thank the reviewer for this comment. To understand how loss of C18orf21 impacts MRP RNA levels, we have now performed both RNA sequencing and qPCR on RNA extracted from control cells, C18orf21 knockout cells, RPP14 knockout cells, and RPP21 knockout cells. We found that both C18orf21 and RPP14 knockout cells had lower levels of RNase MRP RNA than control or RPP21 knockout cells. These results suggest that C18orf21 is required for the expression or stability of MRP RNA.

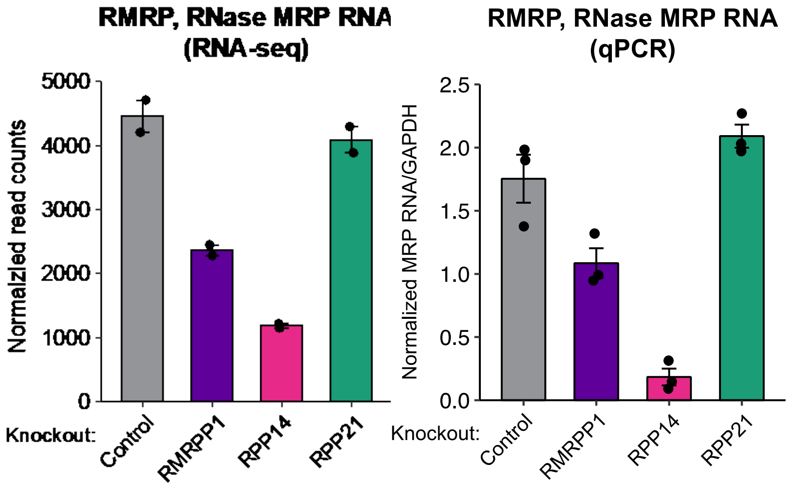

To monitor RNase MRP assembly in C18orf21 knockout cells, we assessed RNase MRP subunit localization. Ribosomal RNA is transcribed and processed in the nucleolus. Therefore, RNase MRP must be in the nucleolus for rRNA processing. By analyzing RPP14 localization in C18orf21 knockout cells, we found that eliminating C18orf21 prevents RPP14 from localizing to the nucleolus where rRNA is processed. Combined with the decrease in levels of RNase MRP RNA observed in our qPCR and RNA-seq assays, we propose that lower MRP RNA abundance and loss of some subunits of the complex both contribute to the changes in 40S ribosome biogenesis we observed. We agree with the reviewer that RNase MRP could retain some of its activities in this scenario and have updated the text to mention this possibility.

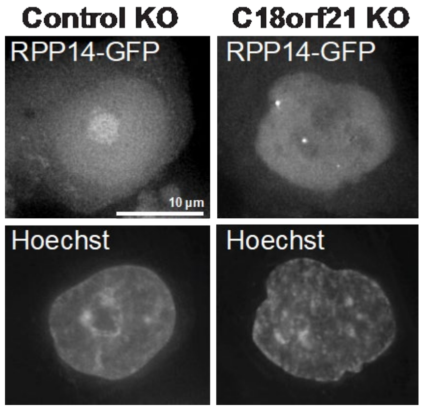

To address how loss of C18orf21 effects RNase MRP cleavage of site 2 in ITS1, we performed qPCR with primers that flank site 2 on RNA from control cells, C18orf21 knockout cells, RPP14 knockout cells, and RPP21 knockout cells. We found an increase in intact site 2 in C18orf21 knockout cells and RPP14 knockout cells, but not in control or RPP21 knockout cells. This suggests that loss of both C18orf21 and RPP14 perturb ITS1 cleavage by RNase MRP.

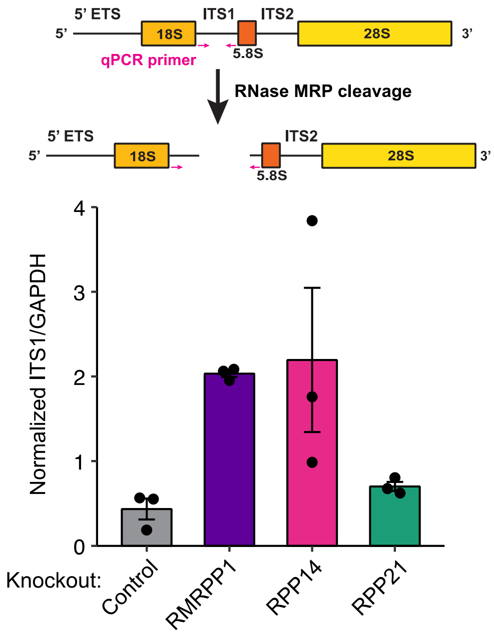

Lastly, we have now analyzed the transcriptome of C18orf21 and RPP14 knockout cells. These results show a very striking similarity of these two knockouts with the exception of H1 RNA, which seems to be destabilized in RPP14 knockout but not C18orf21 knockout. This is consistent with these two proteins being required for RNase MRP activity but RPP14 having an additional role in RNase P.

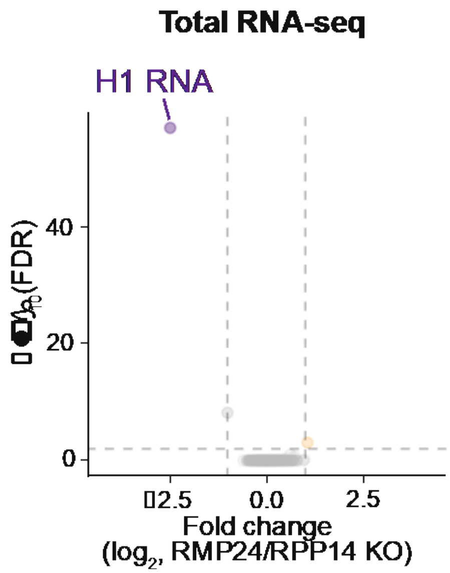

***7:** Figure 6F: Nepro knock down does not seem to have a very significant effect on polysome levels and also leads to a huge increase in the levels of 80S ribosomes. The authors should comment.*

We thank the reviewer for this comment. To address this point, we have now generated NEPRO knockouts using two new guide RNAs and performed polysome profiling on these two pools of NEPRO knockout cells. Consistent with our C18orf21 knockout cells, we found that targeting NEPRO with either guide led to a substantial decrease in the ratio of 40S/60S ribosomes and a decrease in both 80S and polysome levels. Although the change in 40S/60S ratio was observed with all 3 guide RNAs tested, the two additional guides results contrast with our initial guide which showed an increase in the levels of 80S ribosomes. The cells targeted with our initial guide were not viable 2 days post knockout compared to cells being viable for >3 days our two new guides. We now think that our prior guide RNA had a potential off-target effect that complicates the interpretation of the polysome profiling and have removed it from the manuscript as we believe the two new guide RNAs are more consistent and representative.

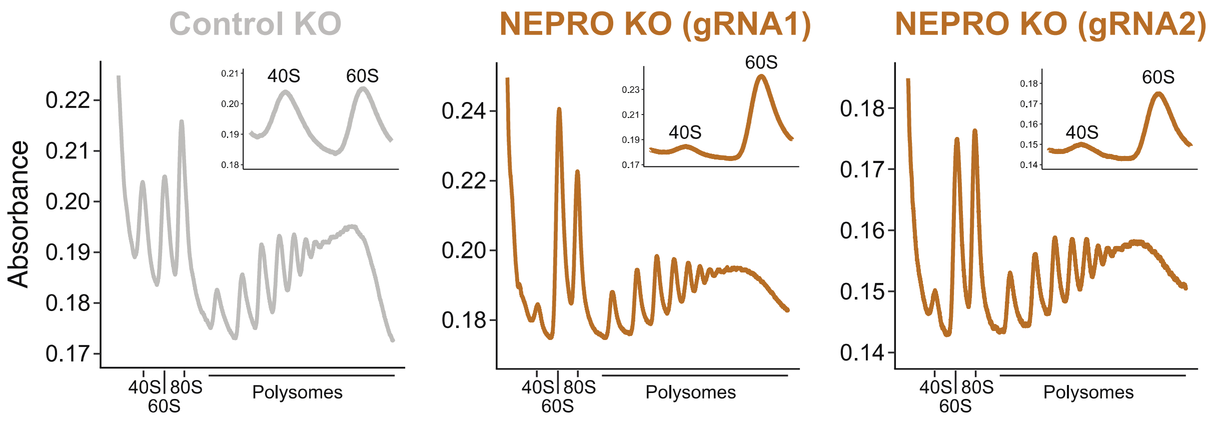

Figure 4A, bottom chimera: it should be 'Rpp21 C-term'
Figure 4E, last lane 'linker IP' not 'ilnker IP'
Line 240: remove second 'that'

We thank the reviewer for pointing out these typos. They have now been fixed.

Reviewer #3:

1: *General comment: the title of the manuscript significantly overstates the actual reach and significance of the reported results; it would be advisable to change it to something more factual.*

We have changed the title to "C18orf21/RMP24 and Nepro/RMP64 are RNase MRP-specific subunits required for 40S ribosome biogenesis"

2: *Ln 39. Protein and even promoter regions mutations are also associated with dysplasias.*

We thank the reviewer for mentioning this. We have now added this information and the corresponding citations.

3: *Ln 42. The role of RNase MRP in 5.8S rRNA processing is not essential, at least in yeast: the formation of the longer form of 5.8S rRNA does not appear to depend on RNase MRP.*

We thank the reviewer for pointing this out. We have removed this portion of the sentence.

4: *Ln 47. PRORPs?*

We appreciate that PRORPs are also important and essential tRNA processing enzymes. However, in the context of the introduction, we believe that the inclusion of PRORPs may be confusing for the reader.

5: *Ln 49. H1 RNA/RMRP are the name reserved for the human RNAs specifically; these are not appropriate names for the duplicated genes. Please rephrase.*

We have removed the gene names from this sentence.

6: *Ln 58-60. Not a really meaningful statement.*

We agree with the reviewer and have removed the statement from the manuscript.

7: *Ln 62,63. Same as above*

We agree with the reviewer and have removed the cited lines from the manuscript.

8: *Ln 65. Role for this protein or for the whole RNase MRP complex that requires it?*

We agree with the reviewer and have rephrased the sentence.

9: *Ln 66. Consider rephrasing*

We agree with the reviewer and have rephrased the sentence.

10: *Ln 69,70. This is an overstatement*

We agree with the reviewer and have rephrased the sentence.

11: *Ln 74,75 Consider rephrasing*

We agree with the reviewer and have rephrased the sentence.

12: *Ln 163. Suggesting a direct interaction in the context of the RNase MRP holoenzyme : this is a strong indication, but not a real evidence or "illustration".*

We apologize that this sentence was not clear. To address the reviewer's point, we have updated this sentence to clarify that we are only speaking about the interaction between C18orf21 and RPP29.

13: *Ln 247,248. How does this compare to what is known for yeast? See also Lindahl et al. 2009 and Li et al. 2021.*

We thank the reviewer for pointing out these studies. We rephrased the sentence and added these citations.

14: *Ln 305. The authors may want to be a bit more cautious with this statement as potential alternative roles for RMRPP1 cannot be completely excluded at this point.*

We agree with the reviewer and have rephrased the sentence.

15: *Ln 309. See comment to ln 39.*

This information has been added to the sentence and citations identifying the promoter mutations have been added.

16: *Ln 339. …is likely to have an altered interaction…*

We thank the reviewer for the suggestion and have updated the text.

17: *Ln 346-348. The authors may want to revisit this sentence.*

We thank the reviewer for this comment and have revised this sentence.

18: *Ln 359. Preserving ALL other roles? Please rephrase as it is an overstatement as it is: not ALL Nepro roles have been analyzed.*

We agree with the reviewer and have removed this phrase.

19: *Ln 367. Substrate recognition by yeast RNase MRP has been previously studied biochemically: see Esakova et al. 2011 and 2013.*

We thank the reviewer for pointing out these studies. We have now cited and described their findings and have rephrased the sentence.

20: *Ln 424 and below. Was the DNA/protein sequence altered?*

We thank the reviewer for pointing this out. We have updated the text to describe these in more detail. The protein sequence was not altered outside of the indicated disease-associated mutations. In certain situations, the constructs were codon optimized. For example, C18orf21 and RPP29 were codon optimized for expression in *E. coli*. We have now added this information to the text.

21: *Ln 498. Indicating acceleration (as opposed to rpms) would be more useful.*

We have changed the text to describe the acceleration used.

22: *Ln 503. Precision protease? Please correct.*

We thank the reviewer for catching this typo. It has now been corrected.

We again thank the reviewers for their constructive comments and suggestions. For the revised manuscript, we have addressed the remaining comment raised by reviewer #1.

Reviewer #1

The revised paper has been significantly improved, and my specific comments have generally been suitably addressed.

I am, however, surprised by the new FISH results shown in the response to referees and Ext. Fig. 6D-F. The 5.8S and 28S rRNAs are expected to be stably base-paired in the pre-rRNA prior to separation, and would not be expected to show differential localization. In bacteria and archaea, they are both regions of the 23S rRNA. Accumulation of 5.8S in the absence 28S would appear to require substantial 28S degradation, which is inconsistent with the other conclusions of the paper. It seems likely that there is some artefact here and this issue should at least be mentioned in the text.

> We thank the reviewer for this comment. We agree that there are multiple interpretations of this data. As this point is not central to our conclusions, we have chosen to remove this sentence from the manuscript.

Reviewer #2

The authors have provided an extensively revised version of their original manuscript with substantial text modifications and numerous additional data. They have in my view appropriately answered all the issues raised by the reviewers.

> We thank the reviewer for their positive feedback.

Reviewer #3

The authors have addressed all concerns and suggestions that this reviewer had. The revised version of manuscript is substantially improved. Of note, the main conclusion of this work is consistent with the recently published results on the protein composition of human RNase MRP that have been obtained using a different approach (Alexandrov group, Cell Rep. 44(6) 115752).

> We thank the reviewer for their positive feedback. We have updated our manuscript to include this citation.

We again thank the reviewers for their constructive comments and suggestions. For the revised manuscript, we addressed the remaining outstanding concern raised by reviewer #1 as described below.

Reviewer #1 (Remarks to the Author):

I am not convinced that the most appropriate way to deal with a question about the data presented is to omit mention of the issue. Ext. Fig. 6F is not now referenced in the text, and should at least be called. However, the point is fairly minor, and I would not wish to block publication on these grounds.

A core finding of our paper is the identification of C18orf21/RMP24 as a subunit of the RNase MRP complex that is required for the enzyme to process ribosomal RNA precursors. For the first version of the manuscript, we found that this role was specifically related to the biogenesis of the 40S ribosome subunit based on difference that we observe in the ratio of 40S:60S ribosome subunits in on our polysome profiling experiments. During the first round of reviews, one of the reviewers commented on this interesting behavior such that we chose to conduct additional complementary experiments to verify this point. For the fluorescence in situ hybridization (FISH) experiment included as Extended Figure 6 as part of the revised manuscript, our goal was to compare the levels of 18S and 28S rRNA present in the cytoplasm following the depletion of C18orf21/RMP24.

For this experiment, we used several different FISH probes designed to detect the different ribosomal RNAs. We used probes that individually hybridize to the 18S or 28S rRNAs. Additionally, we included a 5.8S probe designed to detect the localization of all rRNA subunits derived from the 45S rRNA pre-cursor. In each case, we chose these probes based on prior published studies with validated experimentation (Gai et al. *Cell Research* 2022; Wei et al. *bioRxiv* 2024). Due to the nature of the processing pathways and the fact that we are visualizing intact cells, these probes will hybridize to both mature (cytosolic) and pre-cursor (nucleolar) species.

Consistent with the observed decrease in 40S ribosomes in our polysome profiling experiments following the depletion of RMP24/C18orf21, we found that RMP24 knockdown led to a substantial decrease in the abundance of 18S rRNA in the cytoplasm based in FISH experiments. Additionally, we found that both the 28S and 5.8S rRNA species were still present at levels comparable to control cells in the cytoplasm in agreement with our polysome profiling results. We have now quantified this staining for the 5.8S rRNA probe, and find that there is no distinguishable difference for its levels in the cytoplasm. These results demonstrate that there is a specific reduction in the mature 18S rRNA in RMP24 knockout cells, consistent with our polysome profiling, as well as additional experiments conducted using the bioanalyzer analysis and RNA-sequencing results. Thus, there is a substantial reproducibility in this core discovery across experimental modalities.

In addition to the well-behaved localization for these rRNA species, we also observed that there was an accumulation of the 5.8S, but not 28S rRNA in the nucleolus in RMP24 knockdown cells. This is the discrepancy that was highlighted by the reviewer as 28S and 5.8S biogenesis are thought to be closely coupled. This result is not central

to the main point in our paper, especially given the large redundancy of approaches that we used to evaluate this point, but we recognize that the staining behavior for the 5.8S probe was unexpected.

We believe that there is either a technical or biological explanation for this observed FISH localization for the 5.8S probe. First, as a technical explanation, some of the aberrant rRNA intermediates created in the C18orf21 knockout could have a structure that allows the probe to bind whereas intermediates formed in the control cells do not. Similarly, it is possible an unrelated RNA aberrantly generated in C18orf21 knockout cells may be able to bind to the 5.8S rRNA probe.

Second, as a biological explanation, incomplete processing of the 5.8S rRNA may lead to the accumulation of some precursors that contain 5.8S rRNA sequences, but not the 28S rRNA in the nucleolus. Indeed, previous work found that knock out of the RNase MRP RNA resulted in the formation of rRNA intermediates termed “X” and “21SL3’ ” (see Figure 5D-E from Goldfarb and Cech, *Genes and Dev* 2017). These intermediates contain 18S, ITS1, and 5.8S rRNA, but not the 28S rRNA. In these MRP RNA knockouts, the authors did not observe a decrease in abundance of the mature 5.8S rRNA species matching our observation of no change of 5.8S rRNA abundance in the cytoplasm. Additionally, in our RNA sequencing analyses, we observed an increase in reads at the ITS1/5.8S junction, but not the 5.8S/ITS2 junction, suggesting that there may be similar intermediate precursors being formed in our C18orf21 knockout cells. Indeed, Goldfarb and Cech also observed an increase in nucleolar 5.8S staining [text and image redacted]. Overall, the findings from the Cech lab are consistent with the accumulation that we observed for 5.8S, but not 28S rRNA in the nucleolus. Thus, this unexpected behavior is not unique to our work. For the revised version, we specifically mentioned this in the text.

Since our primary objective was to monitor the localization of 18S and 28S rRNA subunits as an alternate approach to evaluate the observed difference in abundance of the 40S and 60S ribosomal subunits observed in polysome gradients, we believe these inconsistencies in the behavior of 5.8S rRNA FISH probe specifically for the nucleolar staining are best suited for follow up studies on the precise mechanisms of 5.8S processing.

To address the reviewer's comment, we have updated the text to succinctly address our interpretation and to acknowledge previous work that shows for many organisms, including yeast, that base pairing of the 5.8S and 28S is necessary for their maturation, suggesting the behavior of the 5.8S and 28S should be identical. Finally, each panel of Extended Figure 6 is now called out in the text as well.
